# Integrated Biorefineries for Food Waste and Food-Processing Side Streams Through Feedstock-Oriented Design and Sustainable Process Integration

**DOI:** 10.3390/foods15203692

**Published:** 2026-10-16

**Authors:** Ersilia Daniela Lazăr Coșbuc, Paula Sânziana Oprea Blanaru, Elena-Diana Ungureanu-Comăniță, Carmen Teodosiu, Maria Gavrilescu

**Affiliations:** 1 Department of Environmental Engineering and Management, “Cristofor Simionescu” Faculty of Chemical Engineering and Environmental Protection, “Gheorghe Asachi” Technical University of Iasi, 73 Mangeron Blvd., 700050 Iasi, Romania; ersilia_cosbuc@yahoo.com (E.D.L.C.); paula-sanziana.oprea@student.tuiasi.ro (P.S.O.B.); elena-diana.comanita@staff.tuiasi.ro (E.-D.U.-C.); 2 Academy of Romanian Scientists, 3 Ilfov Street, 050094 Bucharest, Romania; 3 Academy of Technical Sciences of Romania, 26 Dacia Blvd., 030167 Bucharest, Romania

**Keywords:** cascade valorization, circular bioeconomy, food waste, integrated biorefinery, process integration, technology readiness level

## Abstract

Food waste represents a renewable carbon resource with considerable potential for the production of bioenergy, platform chemicals, biomaterials, and other high-value products within integrated biorefinery systems. However, the heterogeneous composition of food waste, together with the diversity of available conversion technologies, complicates the selection of efficient and economically viable valorization strategies. Successful food-waste valorization requires a systems-oriented approach that integrates individual technologies with feedstock characteristics, pretreatment, conversion pathways, and resource recovery into coherent biorefinery configurations. This structured critical review synthesizes literature identified through Scopus, Web of Science Core Collection, and ScienceDirect for the period January 2015–September 2026, and critically examines the evolution of food-waste biorefinery concepts from conventional processing to integrated, cascade, circular, and near-zero-waste systems, highlighting their engineering principles, advantages, and limitations. Current pretreatment technologies and biological, chemical and thermochemical conversion pathways are comparatively evaluated with respect to process performance, technology readiness and industrial applicability. Particular attention is given to integrated biorefinery design, emphasizing feedstock-oriented process selection, cascade valorization, process integration and resource recovery. The complementary roles of life-cycle assessment, techno-economic analysis, circularity metrics and technology readiness are discussed as key tools for supporting decision-making and industrial implementation. Future progress will depend on combining mature conversion technologies with digitalization, adaptive process management and integrated system design to maximize resource efficiency while advancing the transition toward a sustainable circular bioeconomy.

## 1. Introduction

Food loss and food waste represent related but distinct sources of inefficiency within modern food systems. Food loss generally occurs upstream during production, post-harvest handling, storage, and processing, whereas food waste refers primarily to food discarded at the retail, food-service, and household stages. Both involve losses of resources already invested in food production, including land, water, energy, fertilizers, labour, infrastructure, and capital, and therefore generate environmental burdens beyond those associated with their subsequent management. According to the United Nations Environment Programme, approximately 1.05 billion tonnes of food waste were generated at the household, food-service, and retail stages in 2022, equivalent to 132 kg per capita and nearly 19% of the food available to consumers. Households accounted for almost 60% of this food waste, followed by food services (28%) and retail (12%) [1]. Food loss and food waste together also contribute substantially to global greenhouse-gas emissions and increase pressures on agricultural land, freshwater resources, biodiversity, and food security [2,3]. Although these broader impacts reinforce the importance of prevention across the food supply chain, the present review focuses specifically on food waste and defined food-processing residual streams as potential biorefinery feedstocks.

Food-waste prevention and food-waste valorization represent distinct levels of the food-use and waste hierarchy. The first priority is prevention, including avoidance of surplus generation and reduction in avoidable food loss and waste. Where edible surplus nevertheless arises and remains safe and suitable for human consumption, redistribution or other forms of reuse as food should take precedence over non-food applications. Where human consumption is no longer appropriate, use as animal feed may represent a higher-value option for eligible streams, subject to applicable safety and regulatory requirements. Only residual streams that cannot appropriately remain within food or feed uses should be considered for material recovery and biorefinery valorization, including the recovery of functional compounds, ingredients, chemicals, biomaterials, and recyclable nutrients. Biochemical conversion to fuels and other products and, subsequently, energy-recovery routes provide further options for fractions that are unsuitable for higher-value uses. Accordingly, biorefinery conversion should complement rather than compete with prevention, redistribution, food/feed reuse, or established higher-value material uses [4,5]. The appropriate route must therefore be determined according to feedstock characteristics, safety and regulatory constraints, existing uses, and the environmental and economic consequences of diverting a stream from its current application.

Within this framework, avoidable food waste refers to food whose generation or disposal could reasonably have been prevented, whereas unavoidable food waste comprises inedible fractions or residual materials that arise despite appropriate prevention measures. Edible surplus refers to food that remains suitable for human consumption and should therefore be prioritized for redistribution rather than biorefinery processing. Food-processing residues, by-products, and side streams require separate consideration because they may already serve established food, feed, ingredient, fertilizer, or industrial markets. Their diversion to a biorefinery should therefore not be assumed to provide an environmental benefit; rather, the existing use should form part of the reference scenario against which an alternative valorization route is evaluated.

The feedstocks considered in this review comprise several legally and technologically distinct categories, including household and food-service food waste, retail residual streams, former foodstuffs, food-processing residues, by-products, and other industrial side streams. For clarity, the term “food waste” is used specifically for materials that have become waste under the applicable regulatory framework, whereas “food-processing residues” and “side streams” are used as broader technical terms for residual material flows generated during food production and processing. The term “by-product” is reserved for streams that meet the applicable legal criteria for by-product status and therefore are not classified as waste. Former foodstuffs constitute a further distinct category comprising food products originally produced for human consumption that are no longer intended for that purpose and whose subsequent legal status and permitted uses depend on the applicable regulatory framework. Regulatory classification is jurisdiction-specific and may depend on the origin, intended use, safety characteristics, processing history, and applicable waste, food, feed, and animal-by-product legislation. Consequently, materials such as whey, fruit pomace, brewer’s spent grain, and other food-processing streams should not automatically be classified as waste. Heterogeneous municipal and post-consumer food waste generally requires more extensive sorting, stabilization, and pretreatment, whereas many industrial side streams are generated under more controlled conditions and may have a more consistent composition; these differences affect pretreatment requirements, process control, product quality, and economic feasibility [6,7]. Throughout this review, the broader expression “food-derived residual streams” is therefore used when collectively referring to food waste together with food-processing residues, by-products, former foodstuffs, and other side streams whose legal status may differ among jurisdictions. These distinctions are important because legal classification may affect collection and transport requirements, permitted recovery routes, use in food or feed applications, processing obligations, and, where applicable, end-of-waste requirements.

A biorefinery is often compared with a petroleum refinery because both systems convert raw materials into several products through integrated processing. However, food waste presents specific challenges that make its processing considerably more complex. Its composition can vary widely according to source, season and collection system, while its high moisture content and rapid biodegradation can create additional difficulties during transportation, storage and processing. Contamination and the presence of unwanted materials may further increase the need for sorting, pretreatment and quality control [6,8]. The feasibility of a food-waste biorefinery depends not only on the amount of carbon and nutrients that can be recovered but also on the efficiency with which these resources can be converted into products of sufficient quality and market value. Energy requirements, separation and purification costs, feedstock logistics and process integration must therefore be considered together when evaluating the technical and economic feasibility of these systems [5].

Numerous studies have demonstrated the potential of food-derived residues for producing biofuels, platform chemicals and a wide range of bio-based products, including microbial lipids, enzymes, biosurfactants, polyhydroxyalkanoates, pigments and single-cell proteins [6,8,9,10,11,12]. Several reviews published during the last five years have advanced the field substantially. Tsegaye et al. (2021) presented food-waste biorefineries as an enabling pathway for producing fuels, platform chemicals, and bio-based materials within a circular bioeconomy [13]. Mohanty et al. (2022) critically summarized biological and thermochemical routes for bioenergy generation, while emphasizing the suitability of highly biodegradable food waste for microbial conversion [14]. Awasthi et al. (2022) examined fungal biorefinery strategies, demonstrating the potential of fungi to generate enzymes, organic acids, proteins, and other products under comparatively mild conditions [15]. Roy et al. (2023) broadened the discussion by analysing environmental and economic choices in food-waste valorization and concluded that sustainability and social considerations remain less developed than technical conversion studies [16]. Sarangi et al. (2023) reviewed emerging bioconversion technologies for food-processing residues from a circular-bioeconomy perspective [7]. More recent syntheses have addressed biotechnological valorization of agri-food residues, integrated biological processing of urban food waste, life-cycle performance, and multi-criteria selection of waste-management pathways [17,18,19].

Despite these advances, recent reviews have predominantly focused on individual conversion technologies, specific feedstocks or product classes, while the relationships among feedstock characteristics, pretreatment and fractionation, conversion-pathway selection, product hierarchy, process integration, technology maturity, and system-level sustainability have received less integrated treatment [7,13,14,15,16,17,18,19]. The specific contribution of the present review is therefore to organize these elements within a feedstock-oriented, system-level framework that links the characteristics and existing uses of food-derived residual streams to appropriate pretreatment and conversion pathways, cascade valorization, resource and energy integration, and complementary assessment through TRL, LCA, TEA, and circularity indicators. Rather than identifying a universally optimal configuration, the review critically examines how these interacting factors can inform context-specific biorefinery design and industrial implementation.

In spite of this growing body of literature, several limitations continue to restrict its value for biorefinery design. First, research remains fragmented across feedstocks, pretreatment methods and conversion pathways, with many studies optimizing individual unit operations without evaluating their effects on downstream processing, resource integration or residue management [8,20,21]. The concept of an integrated biorefinery is often used inconsistently, ranging from the simple co-location of processes to fully integrated systems with coordinated mass, energy and resource flows.

Second, comparisons among published studies remain difficult because experimental conditions and performance metrics vary considerably. Differences in feedstock composition, pretreatment strategies, inocula, process conditions and yield definitions frequently prevent direct comparison, while laboratory-scale experiments performed under highly controlled conditions may not reflect industrial performance. Consequently, reported yields alone provide only limited guidance for technology selection [22,23,24,25].

Third, claims regarding circularity and sustainability are frequently based on the use of waste-derived feedstocks without comprehensive evaluation of the overall system. However, environmental performance is shaped by pretreatment, energy demand, transport, downstream processing, allocation procedures, and the selected reference scenario. Likewise, technological feasibility does not necessarily indicate industrial applicability, since laboratory studies rarely account for feedstock heterogeneity, contamination, scale-up constraints, regulatory requirements or market conditions [16,18,26,27,28]. These limitations often result in optimistic assessments of emerging technologies while underestimating the advantages of mature industrial processes.

These limitations underscore the need for a system-oriented assessment of food-waste biorefineries that moves beyond individual conversion technologies to account for interactions among feedstock characteristics, pretreatment, conversion pathways, product recovery, energy integration, and residual-resource management [29,30,31]. Biorefinery performance should therefore be evaluated in terms of its capacity to combine complementary conversion routes and generate a balanced product portfolio, with the yield of individual products considered within the broader context of overall system performance. In this context, coordinated production does not necessarily imply the concurrent formation of multiple products within a single process, but rather their coordinated generation across a biorefinery in which feedstock fractions, intermediates, utilities, and residual streams are strategically exchanged and valorized. This configuration can create functional synergies: bioenergy pathways may enhance process self-sufficiency and provide an effective outlet for residual organic matter, whereas biochemical pathways can preserve greater molecular value and increase the economic value recovered from the feedstock [32,33]. Such complementarity, however, should not be assumed solely from the coexistence of multiple conversion routes. Its benefits must be demonstrated through integrated material and energy balances, techno-economic analysis (TEA), life-cycle assessment (LCA), and realistic consideration of feedstock variability, process scale, and market conditions [34,35,36].

Against this background, this review critically examines food-waste biorefineries designed for the coordinated production of bioenergy and value-added bioproducts from source-separated household, retail, food-service, and industrial residues. Particular attention is given to biological conversion pathways and to the ways in which pretreatment, fractionation, resource recovery, and energy integration can enhance overall system performance. Thermochemical technologies are considered where they support these functions, particularly through residue valorization, energy recovery, and biochar production. Waste prevention, redistribution, composting, and conventional disposal are retained as reference strategies within the waste hierarchy, thereby positioning biorefinery-based valorization within the broader food-waste management framework while keeping these alternatives outside the primary scope of the review.

The review pursues four main objectives: (i) to establish a clear conceptual framework distinguishing waste treatment, single-product valorization, cascade processing, and integrated food-waste biorefineries; (ii) to evaluate how feedstock composition, heterogeneity, and quality influence pretreatment, conversion pathways, and product selection; (iii) to critically assess the technical, environmental, and economic performance of major bioenergy and biochemical production routes, including process integration, downstream processing, and technology readiness; and (iv) to identify the principal barriers, knowledge gaps, and future priorities for developing flexible, economically viable, and sustainable food-waste biorefineries.

**Figure 1:**
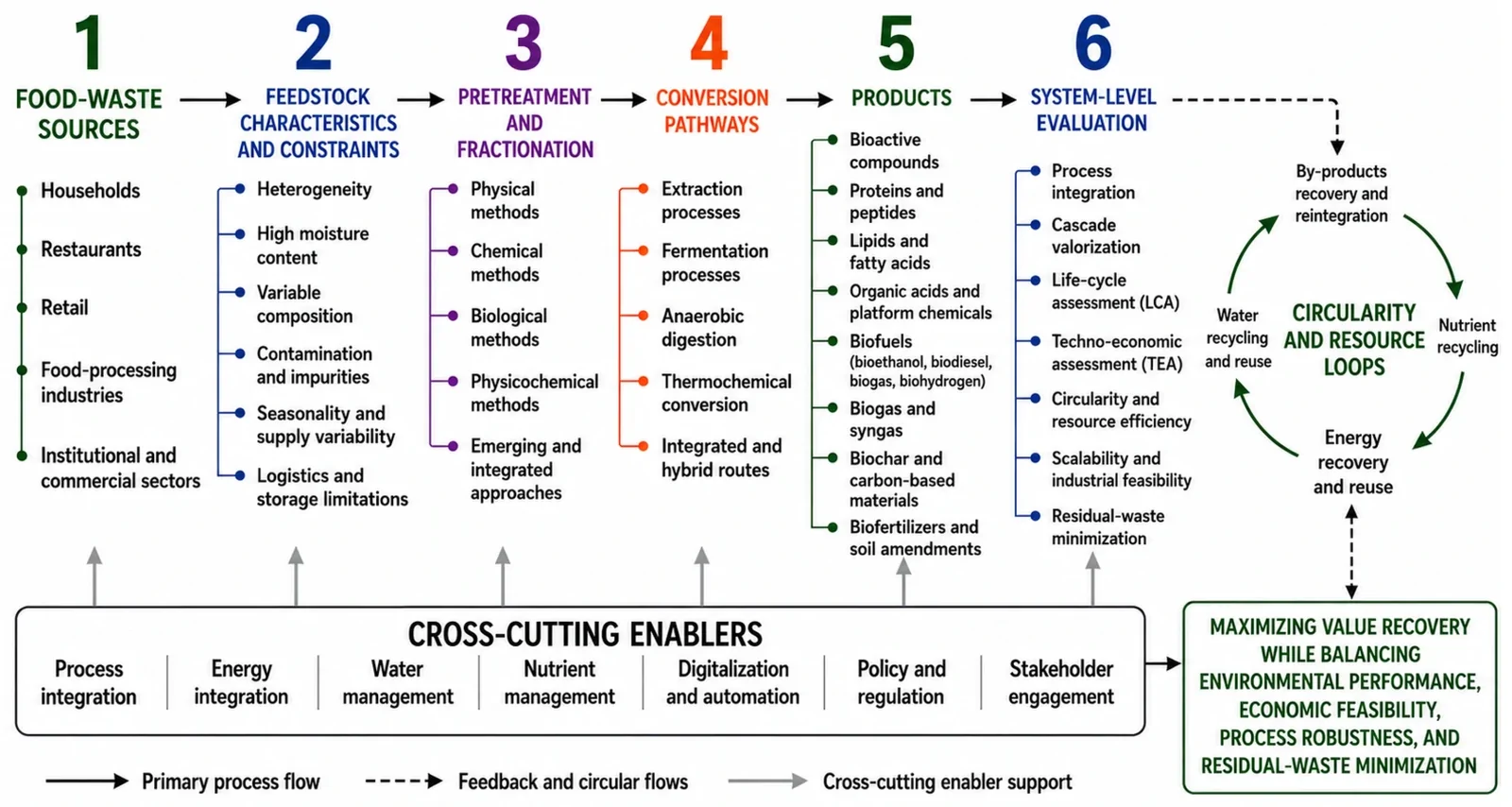
Scope and analytical framework of the review on integrated food-waste biorefineries. The framework links major food-waste sources and feedstock-related constraints with pretreatment and fractionation strategies, biological and thermochemical conversion pathways, and the coordinated production of high-value biochemicals, bioenergy, materials, and nutrient-rich products. The lower analytical layer highlights the system-level criteria required to evaluate integrated biorefineries, including cascade valorization, process integration, environmental life-cycle assessment, techno-economic feasibility, circularity, scalability, and residual-waste minimization.

Figure 1 summarizes the analytical framework adopted throughout this review, linking feedstock characteristics with pretreatment, biological and thermochemical conversion pathways, product recovery, process integration, and system-level evaluation. This framework reflects the central premise of the review: the performance of a food-waste biorefinery should be assessed at the system level, integrating the yield and productivity of individual conversion processes with their contribution to overall system performance.

## 2. Review Methodology

This study was conducted as a structured critical literature review to identify, organize, and critically assess the available evidence on integrated food-waste biorefineries. The literature search was conducted using Scopus, Web of Science Core Collection, and ScienceDirect, selected for their broad coverage of environmental engineering, biotechnology, waste management, bioenergy, and biorefinery research. The search covered the period from January 2015 to September 2026, with the final database search performed on between May-September 2026. The search was restricted to publications in English. Peer-reviewed research articles and review articles were considered as the principal sources of scientific evidence, while relevant institutional reports and policy documents from recognized international and governmental organizations were additionally consulted where necessary to support definitions, regulatory context, food-waste statistics, and established policy frameworks.

Search terms were organized into four thematic groups: (i) feedstock-related terms, including “food waste”, “food-processing waste”, “agri-food waste”, “food residues”, and “municipal food waste”; (ii) processing-related terms, including “pretreatment”, “fractionation”, “fermentation”, “anaerobic digestion”, and “thermochemical conversion”; (iii) valorization- and product-related terms, including “biorefinery”, “biofuels”, “biochemicals”, “bioproducts”, “resource recovery”, and “cascade valorization”; and (iv) system-assessment terms, including “process integration”, “circular biorefinery”, “life-cycle assessment”, “techno-economic assessment”, and “technology readiness level”. Terms from these groups were combined using database-specific Boolean operators and search-field syntax to identify studies relevant to integrated food-waste valorization. The complete database-specific search strategies, including the exact search strings, search fields, search dates, restrictions applied, and the number of records retrieved from each database, are provided in Supplementary Table S1. Reference lists of key review articles and highly relevant primary studies were additionally screened to identify relevant publications not captured by the database searches.

The retrieved literature was evaluated through successive screening and eligibility stages. After removing duplicate and clearly unrelated records, titles and abstracts were assessed for relevance to the scope of the review. Publications were retained for full-text assessment when they addressed food-derived residual streams and at least one relevant component of the biorefinery chain, including feedstock characterization, pretreatment or fractionation, biological or thermochemical conversion, product recovery, process integration, resource recovery, technology readiness, or environmental and economic performance. For eligibility assessment, a study was considered to provide sufficient feedstock characterization when the food-derived residual stream was clearly identified by source and/or type (e.g., household or catering food waste, fruit and vegetable residues, dairy by-products, cereal-processing residues, spent coffee grounds, or other defined food-processing side streams) and could be linked unambiguously to the conversion or recovery pathway investigated. Studies were eligible when they reported experimental, pilot-scale, demonstration-scale, modelling, life-cycle, techno-economic, or process-integration evidence relevant to at least one component of the food-waste biorefinery chain. For experimental studies, sufficient methodological information on the feedstock and process conditions was required to allow interpretation of the reported conversion, recovery, yield, or product-performance data. Studies referring only generically to “biomass”, “organic waste”, or “municipal solid waste” without distinguishing a food-derived fraction were not used as primary evidence for feedstock-specific comparisons.

Peer-reviewed research articles and review articles constituted the principal scientific evidence base. Conference abstracts/proceedings, theses and dissertations, and patents were not included as primary evidence in the comparative synthesis because of differences in peer-review status, methodological detail, and data accessibility. Grey literature was considered selectively when necessary to document regulatory definitions, policy frameworks, sector-level statistics, technology deployment, technology readiness, or commercial implementation that could not be adequately characterized from peer-reviewed publications alone. Such sources included reports and technical documents issued by governmental bodies, international organizations, research institutions, and other identifiable organizations. Information concerning industrial or commercial technologies was considered when traceable to an identifiable organization or facility and when sufficiently specific to establish the technology, feedstock, process configuration, scale, or implementation status. However, commercial or organizational claims unsupported by sufficient technical information were not treated as equivalent to peer-reviewed evidence of process performance. Earlier publications were retained when they represented foundational studies, benchmark technologies, or relevant evidence of industrial implementation; otherwise, emphasis was placed on the most recent evidence available within the defined search period.

The study-selection process comprised database identification, duplicate removal, title and abstract screening, full-text eligibility assessment, and final inclusion in the qualitative synthesis. The numerical progression of records through these stages, together with the principal reasons for exclusion at the full-text stage, is summarized in Supplementary Figure S1. Screening and eligibility assessment were conducted by three authors (E.-D.U.-C., C.T., and M.G.). Potentially eligible or uncertain records were discussed among these authors, and final decisions regarding inclusion or exclusion were reached by consensus.

Following selection, publications were classified and organized using a hierarchical analytical framework that considered multiple study characteristics beyond publication date or reported product yield. Studies were first differentiated according to the origin, composition, heterogeneity, and dominant fractions of the food-waste feedstock, with particular attention to carbohydrate-rich, protein-rich, lipid-rich, and heterogeneous/mixed waste streams. They were subsequently classified according to pretreatment or fractionation strategy, conversion pathway, target products, downstream recovery requirements, and degree of process integration. At the system level, the reported configurations were interpreted in terms of conventional valorization, integrated biorefinery, cascade processing, circular biorefinery, and near-zero-waste approaches. This organization follows the conceptual framework of the review, in which feedstock characteristics are linked sequentially to pretreatment/fractionation, conversion pathways, product recovery, process integration, and ultimately system-level performance. The selected evidence was then subjected to a comparative multi-criteria critical synthesis.

Following study selection and classification, the evidence was synthesized using a structured narrative comparative approach. For each technological route, information was extracted, where reported, on feedstock compatibility and characteristics, conversion or recovery performance, pretreatment requirements, energy and chemical inputs, downstream-processing needs, product type and potential value, technology readiness level (TRL), scalability, opportunities for process integration, residual-waste minimization, and resource recovery. Where available, environmental and economic evidence was additionally considered through life-cycle assessment (LCA) and techno-economic assessment (TEA). These criteria were used as a common qualitative framework for organizing and comparing the heterogeneous evidence base; no numerical scores, weighting factors, composite sustainability indices, or formal multi-criteria ranking procedure were applied.

The comparative assessment was therefore qualitative rather than a standardized multi-criteria decision analysis. Statements concerning industrial applicability, scalability, integration potential, or sustainability were interpreted in the context of feedstock characteristics, process configuration, operating conditions, study scale, and the type of evidence available. Laboratory-scale studies were primarily considered as evidence of technical feasibility and emerging opportunities, whereas pilot-, demonstration-, and commercial-scale evidence was given greater relevance when discussing scalability and industrial implementation. Where studies reported divergent outcomes, these differences were retained in the synthesis and interpreted in relation to differences in feedstock composition, operating conditions, system boundaries, technological scale, and assessment assumptions rather than being combined into a single score or overall ranking.

Particular care was taken not to treat individual performance indicators as sufficient evidence of overall sustainability or industrial applicability. Accordingly, TRL, LCA, TEA, process integration, residual-waste minimization, and resource recovery were considered complementary dimensions of the narrative assessment rather than components of a formal composite score. Comparative conclusions were therefore formulated in terms of reported advantages, limitations, relative tendencies, and context-dependent performance rather than universal superiority of one technological route over another.

A quantitative meta-analysis was not performed because direct numerical comparison among the reviewed studies would be affected by substantial differences in feedstock composition, pretreatment conditions, inocula or microorganisms, operating conditions, process scale, analytical procedures, and definitions of yield and efficiency. Moreover, laboratory-scale yields do not necessarily represent industrial performance. Therefore, reported performance values were interpreted within their original experimental and system context, and greater emphasis was placed on consistent trends and converging evidence across studies. The final synthesis focused on the relationships between feedstock characteristics, technology selection, product hierarchy, process integration, technological maturity, and system-level sustainability, while assessing individual technologies within this broader analytical context instead of relying solely on maximum reported yields.

Numerical performance values and ranges reported throughout the review were used primarily to characterize the order of magnitude and variability of performance reported for individual technologies rather than as pooled quantitative estimates. Where several studies reported comparable outcomes for sufficiently similar feedstocks, process configurations, and performance metrics, the reported values were considered collectively to define an indicative range. Where substantial differences existed in feedstock composition, operating conditions, analytical basis, process scale, or outcome definition, values were not statistically pooled; instead, representative values or study-specific ranges were reported together with the relevant experimental or technological context. Accordingly, the numerical ranges presented in this review should not be interpreted as exhaustive minimum-to-maximum intervals across all publications identified during the literature search unless explicitly stated otherwise.

Extreme values were not excluded solely because they were numerically high or low. However, values obtained under substantially different experimental conditions, expressed using non-comparable definitions or units, or insufficiently documented to permit meaningful interpretation were not combined within the same indicative range. When unusually high or low values were considered relevant, they were retained as study-specific observations and interpreted in relation to the conditions under which they were obtained. Technology readiness levels (TRLs) were similarly interpreted from the scale and maturity information reported in the cited sources and were used to indicate approximate technological maturity rather than as statistically aggregated values.

Technology readiness levels were interpreted using the European Commission TRL framework, in which maturity is assessed from proof of concept and laboratory validation through demonstration in relevant or operational environments to commercially proven operation. TRL was assigned only with reference to the specific feedstock–conversion pathway–product configuration and scale of implementation described in the supporting literature. Accordingly, the TRL values reported in this review refer, wherever supporting evidence was available, to technologies demonstrated using food waste or defined food-processing residual streams rather than to the maturity of the same products when manufactured from conventional feedstocks. Evidence of laboratory validation, pilot operation, demonstration under industrially relevant conditions, and full-scale or commercial deployment was mapped to the corresponding TRL categories. Where the reviewed literature covered several feedstocks, process configurations, or implementation scales within the same broad conversion pathway, a range is reported to reflect the maturity levels represented by those specific applications. Such ranges are therefore indicative evidence-based intervals and should not be interpreted as a generic TRL assignment to an entire product class. Where application-specific evidence was insufficient to distinguish maturity reliably, no precise generic TRL should be inferred.

For broad product categories such as polyhydroxyalkanoates, single-cell proteins, nutraceuticals, natural pigments, and industrial enzymes, technological maturity is particularly dependent on the specific recovery or production route, feedstock quality, downstream-processing requirements, product specification, regulatory context, and intended application. Consequently, the TRL ranges summarized later are used only as indicative summaries of the food-derived residual-stream applications represented by the cited evidence and not as maturity ratings for the corresponding product classes in general. This approach was adopted to distinguish illustrative or context-specific performance data from broader trends supported by multiple studies.

## 3. Food Waste as a Biorefinery Feedstock

### 3.1. Food Waste Sources and Biorefinery Implications

The performance of a food-waste biorefinery depends as much on feedstock characteristics as on conversion technology. Although food waste is generally regarded as an attractive substrate because of its high content of biodegradable carbohydrates, proteins, lipids, minerals and bioactive compounds, this description alone provides limited guidance for process design. Unlike dedicated energy crops or standardized industrial substrates, food waste comprises heterogeneous residual streams whose composition varies with their origin, processing history, collection system, seasonality and consumer behaviour. Average compositional values are useful for preliminary assessments but insufficient for predicting process performance or selecting appropriate valorization pathways [1,19,37].

In the following discussion, feedstock terminology reflects both material origin and regulatory status. Household, catering, and post-consumer streams that have been discarded are referred to as food waste, whereas process-specific industrial streams are described preferentially as food-processing residues, side streams, or by-products, as appropriate. The latter terminology does not imply a universal legal classification: the same material may be classified as waste, a by-product, a former foodstuff, or another regulated material category depending on the jurisdiction, production context, intended subsequent use, and compliance with applicable legal criteria. Accordingly, the technological classifications used in this review should not be interpreted as assigning legal waste status to all food-derived residual streams considered.

To ensure terminological consistency throughout the review, Table 1 summarizes the principal terms used to distinguish food-derived residual streams according to their origin, material status, and role within the food supply chain. Because regulatory classification may vary among jurisdictions and according to intended use, the terminology used here should not be interpreted as assigning a universal legal status to a particular material stream.

These distinctions are important for biorefinery design because household and post-consumer food waste, mixed municipal biowaste, and food-processing residues, side streams, and legally recognized by-products differ substantially in composition, homogeneity, traceability, contamination risk, regulatory status, and recoverable value. Conversion efficiencies reported for relatively homogeneous industrial streams therefore cannot be directly extrapolated to heterogeneous post-consumer or mixed municipal feedstocks. Feedstock classification should precede technology selection because substrate characteristics strongly influence pretreatment requirements, feasible conversion pathways, product quality, and overall process economics [38,39,40]. Table 2 subsequently summarizes the principal categories of food-derived residual streams considered in this review, emphasizing their material characteristics, operational advantages, processing constraints, and the valorization pathways most consistent with their properties. This classification provides the framework for the subsequent discussion of feedstock-specific conversion strategies.

#### 3.1.1. Household Food Waste

Households generate the largest proportion of consumer food waste worldwide, accounting for approximately 631 million tonnes in 2022 (about 60% of food waste generated at the household, food-service and retail stages) [1]. In the European Union, households represented approximately 53% of measured food waste in 2023 [2]. Although these quantities suggest a substantial resource base, feedstock availability should not be equated with feedstock suitability.

The principal challenge is not insufficient organic content but poor compositional predictability. Household food waste combines carbohydrates, proteins and lipids with inedible materials and varying levels of physical contamination, while its composition changes with dietary habits, socioeconomic factors, seasonality and collection practices. Even source-separated waste may contain plastics, glass, metals and cleaning residues that complicate downstream processing [3]. Laboratory results obtained using homogenized household waste or synthetic mixtures often underestimate the variability encountered under practical operating conditions.

These characteristics favour robust conversion platforms, particularly anaerobic digestion and mixed-culture fermentation, which tolerate feedstock heterogeneity better than highly selective bioprocesses. Higher-value applications remain feasible, but only after effective sorting, de-packaging, homogenization and quality control. Such pretreatment requirements should therefore be considered integral components of the biorefinery when evaluating process feasibility and economics, as they directly influence overall system performance and operational requirements.

#### 3.1.2. Restaurants and Food-Service Establishments

Food-service waste may differ from household food waste not only in collection logistics but also in composition, preparation practices, traceability, contamination profile, and the relative abundance of components such as lipids, proteins, and salt. Restaurants, hotels, hospitals and institutional kitchens generate relatively concentrated waste streams that facilitate separate collection and improve feedstock availability for biorefineries [16,57]. However, predictable collection volumes do not necessarily translate into predictable feedstock quality.

The suitability of these residues depends largely on their origin within the food-service chain. Preparation residues are generally cleaner and more appropriate for targeted valorization, whereas post-consumer plate waste contains higher levels of physical contamination and hygienic risks. Many studies nevertheless combine both fractions into a single “restaurant waste” category, limiting meaningful comparison among reported conversion performances [58,59]. In addition, high concentrations of lipids, proteins, salt, detergents and preservatives may inhibit biological processes through long-chain fatty acids, ammonia formation or increased salinity.

Characterization of food-service waste should extend beyond its chemical composition to include the point of generation, collection practices and the relative proportions of preparation and plate waste. These operational factors frequently determine process performance as much as the substrate itself and should be reported consistently in future biorefinery studies.

#### 3.1.3. Supermarkets and Retail Systems

Retail food waste occupies an intermediate position between post-consumer waste and industrial by-products. It includes unsold fruit and vegetables, bakery products, dairy, meat, ready meals and packaged foods discarded because of visual defects, damaged packaging, overstocking, interrupted refrigeration or expiry of date labels. Unlike household waste, product identity and composition are often known, offering greater opportunities for selective valorization. However, edible surplus that remains safe and suitable for human consumption should be prioritized for redistribution or other food uses and should not be considered a biorefinery feedstock solely because it is unsold. Biorefinery valorization becomes relevant only for fractions that are no longer suitable or available for higher-priority food uses, subject to the applicable safety and regulatory requirements.

The principal limitation of retail waste is not compositional variability but packaging. Although de-packaging technologies recover organic fractions suitable for biological conversion, they may also introduce plastics, metals, inks and other contaminants that restrict the production of food-, feed- or pharmaceutical-grade products [60,61]. The most appropriate valorization pathway depends not only on substrate composition but also on the efficiency of contaminant removal.

Fruit and vegetable residues are particularly attractive because of their high carbohydrate content and rapid biodegradability, supporting sugar recovery, organic-acid production and anaerobic digestion. Nevertheless, these streams should not be considered uniform substrates, as compounds such as limonene in citrus residues or antimicrobial constituents in onion and garlic can inhibit microbial conversion. Feedstock characterization should therefore include inhibitory compounds in addition to bulk composition.

#### 3.1.4. Food-Processing Residues, By-Products, and Side Streams

Food-processing industries generate a wide range of residual streams that may constitute waste, by-products, co-products, or other side streams depending on their production context, subsequent use, and applicable regulatory framework. Technologically attractive examples include fruit pomace, cereal bran, brewer’s spent grain, dairy whey, starch-rich effluents, spent coffee grounds, oilseed cakes, and animal-processing residual streams. Compared with heterogeneous post-consumer food waste, many of these streams provide greater process reproducibility and facilitate the targeted recovery of sugars, proteins, lipids, and other valuable constituents [7,9].

Their industrial origin, however, does not automatically justify diversion to new biorefinery applications. Many processing residues, including whey, brewer’s spent grain, oilseed cakes, and other side streams, may already be utilized as animal feed, food ingredients, fertilizers, or industrial raw materials. Consequently, feedstock-oriented biorefinery design should consider not only chemical composition and conversion potential but also the existing or counterfactual use of the feedstock. Diverting a side stream from an established application may require substitute products or materials to fulfil the displaced function, thereby generating substitution effects that can materially alter both environmental and economic outcomes. The sustainability of a proposed valorization pathway should therefore be evaluated against the relevant reference scenario, including the impacts and economic value associated with the existing use and, where applicable, its substitution [16,36]. A technically feasible route yielding a higher-value product cannot consequently be assumed to represent the environmentally or economically preferable option without such a comparison. Accordingly, an existing food, feed, ingredient, fertilizer, or industrial application should not be treated as having zero value or zero environmental benefit when evaluating an alternative biorefinery route. Diversion to biochemical or energy-oriented conversion is not inherently preferable and should be justified by comparison with the established use, considering substitution effects, processing requirements, product value, and relevant environmental and economic trade-offs.

Industrial residues also present feedstock-specific constraints. Dilute dairy wastewaters increase separation costs, fruit-processing residues may contain inhibitory phenolics or essential oils, while protein-rich animal residues can promote ammonia accumulation and face stricter regulatory requirements. Therefore, although industrial side streams reduce compositional uncertainty, detailed physicochemical characterization remains essential for selecting appropriate conversion pathways and designing robust biorefinery processes [9].

Importantly, the terms “residue”, “side stream”, and “by-product” are not interchangeable legal categories. Classification must be established under the legislation applicable in the jurisdiction where the material is generated and used. Consequently, the inclusion of an industrial food-processing stream in this review reflects its technological relevance as a biorefinery feedstock and does not imply that the material necessarily has legal waste status.

### 3.2. Chemical Composition and Conversion Potential

The chemical composition of a food-derived residual stream is a major determinant of its technical conversion potential, but it is not sufficient on its own to identify the most appropriate valorization pathway. Feedstock-oriented pathway selection should also consider the stream’s existing use, opportunity cost, market function, and the potential substitution effects associated with diverting it to an alternative biorefinery application. The relative proportions of carbohydrates, proteins, lipids and minor constituents govern product selection, pretreatment requirements, process stability and downstream recovery [62,63]. Carbohydrate-rich residues, including bakery waste, cereals, potatoes, fruits and vegetables, are readily converted into ethanol, organic acids and other fermentation products following relatively mild hydrolysis. In contrast, fibrous residues require more intensive pretreatment, while excessive carbohydrate availability may destabilize anaerobic digestion through rapid acidification [64].

Protein-rich feedstocks support the production of enzymes, peptides, amino acids and single-cell proteins, but protein degradation releases ammonia that may inhibit microbial activity depending on process conditions. Protein content alone is an inadequate indicator of conversion performance [65]. Lipids offer high theoretical energy yields and provide substrates for biodiesel, microbial lipids and biosurfactants [63]. However, their hydrophobic nature and the formation of long-chain fatty acids can impair mass transfer and inhibit microbial consortia, reducing process stability despite their high energy potential [65,66]. Minor constituents, although present in lower concentrations, may strongly influence bioconversion. Salts, phenolics, essential oils, cleaning chemicals and trace metals can inhibit microbial activity or affect product quality, indicating that feedstock characterization should extend beyond proximate composition when high-value products are targeted.

### 3.3. Moisture Content

Post-consumer food waste typically contains 74–90% moisture, although individual streams may fall outside this range [10]. High moisture content facilitates pumping, mixing, hydrolysis and wet anaerobic digestion while reducing the need for process water. Conversely, it lowers energy density, increases transport costs, accelerates microbial deterioration and makes thermal drying highly energy intensive.

The impact of moisture depends on the selected conversion pathway. It favours wet fermentation and anaerobic digestion but limits combustion, pyrolysis, oil extraction and processes requiring concentrated sugar solutions [63,67]. Reporting yields only on a dry-matter basis may therefore overlook these practical constraints. Biorefinery performance should instead be evaluated on both dry and wet feedstock bases, together with the energy required for concentration or drying.

### 3.4. Carbon-to-Nitrogen Ratio

The carbon-to-nitrogen (C/N) ratio is widely used to assess the suitability of food waste for biological processes, particularly anaerobic digestion and composting. Reported values generally range from 14 to 37, although considerable variation exists among post-consumer waste streams [10,11]. However, an apparently favourable C/N ratio does not necessarily indicate stable process performance.

The C/N ratio provides no information on carbon bioavailability, nitrogen mineralization, pH, buffering capacity, lipid content, trace elements or inhibitory compounds. Substrates with similar C/N ratios may exhibit markedly different biodegradation behaviour depending on their chemical composition. The C/N ratio should therefore be regarded as a preliminary screening parameter whose interpretation requires consideration of the broader compositional characteristics of the substrate [68,69]. Protein-rich, low-C/N feedstocks may promote ammonia accumulation, whereas fruit, bakery and lignocellulosic residues with high C/N ratios may require nitrogen supplementation. Although co-processing can improve nutrient balance, effective process design also requires consideration of biodegradation kinetics, alkalinity, organic loading, and inhibitory compounds, alongside appropriate C/N values [68,69].

### 3.5. Variability and Seasonal Effects

Food waste is inherently heterogeneous, with composition varying across countries, regions, supply chains, seasons, collection systems and even individual batches. This variability affects moisture, nutrient content, biomethane potential, fermentable substrates, inhibitor concentrations and, ultimately, process performance. Moreover, compositional components are closely interrelated; for example, increasing proportions of fruit and vegetables generally raise moisture and soluble carbohydrate contents while reducing protein and lipid fractions [6].

Seasonal variation is particularly relevant for fresh produce and food-processing industries, where harvest cycles and consumer behaviour alter both feedstock availability and composition. Although these effects are widely acknowledged, they are rarely incorporated into experimental design. Trujillo-Reyes et al. (2024) reported methane yields ranging from 298 to 465 mL CH_4_ g^−1^ VS for seasonal fruit and vegetable mixtures, where VS refers to the volatile-solids basis used for substrate addition in the biochemical methane potential tests, rather than to VS destroyed [12].

Most laboratory studies reduce variability by homogenizing and storing a single feedstock batch, improving experimental reproducibility but overlooking the fluctuations encountered under industrial conditions. Feedstock characterization should therefore incorporate repeated sampling and quantitative measures of variability to capture the full range of compositional fluctuations. From a biorefinery perspective, feedstock flexibility is often more important than maximizing yields under optimized laboratory conditions. Robust process design therefore requires technologies capable of accommodating compositional variation and maintaining stable performance across heterogeneous feedstocks.

## 4. Biorefinery Configurations for Food Waste Valorization

Food waste can be valorized through numerous biological, chemical and thermochemical pathways, yet the organization of these processes within a biorefinery remains inconsistently defined. Terms such as conventional, integrated, cascade, circular and zero-waste biorefineries are frequently used interchangeably, despite representing different levels of process integration and resource recovery. This conceptual inconsistency complicates comparisons among studies and the evaluation of alternative biorefinery designs [70,71].

Although considerable progress has been achieved in developing individual conversion technologies, much less attention has been given to how these technologies should be combined to maximize resource efficiency and overall biorefinery performance. Many studies assess isolated conversion pathways without considering their contribution to an integrated food-waste valorization system [71,72].

In the absence of a universally standardized taxonomy, the terms conventional, integrated, cascade, circular, and near-zero-waste are used in this review as an authors’ analytical framework for comparing alternative biorefinery configurations according to their degree and mode of process integration, resource recovery, and internal resource recirculation. These categories should therefore be interpreted as descriptive and potentially overlapping design configurations rather than as formally standardized or necessarily successive stages of biorefinery development. Their comparative use provides a structured basis for examining differences in resource recovery, technological complexity, and system-level performance [70,71].

For terminological clarity, this review distinguishes between “zero waste” as an aspirational design concept and “near-zero-waste” or “zero-waste-oriented” biorefineries as practical system configurations. The term “zero waste” refers to the theoretical objective of eliminating residual material and energy streams through complete recovery, reuse, or valorization. Because thermodynamic limitations, unavoidable process losses, contamination, product-quality requirements, and economic constraints prevent complete recovery under practical conditions, implemented or realistically proposed systems are referred to here as “near-zero-waste” or “zero-waste-oriented” biorefineries. Accordingly, “zero waste” is used only when discussing the aspirational design principle itself.

### 4.1. Increasing Levels of Biorefinery Integration

Despite the widespread adoption of the term biorefinery, no universally accepted criteria define the degree of process integration required for a biomass conversion system to be considered a genuine biorefinery. Existing definitions emphasize the sustainable production of multiple products and energy through integrated conversion technologies [38,40], yet they provide little guidance on how integration should be achieved or evaluated. Similar processing schemes are frequently described using different terminology, whereas substantially different systems are often grouped under the same concept.

**Figure 2:**
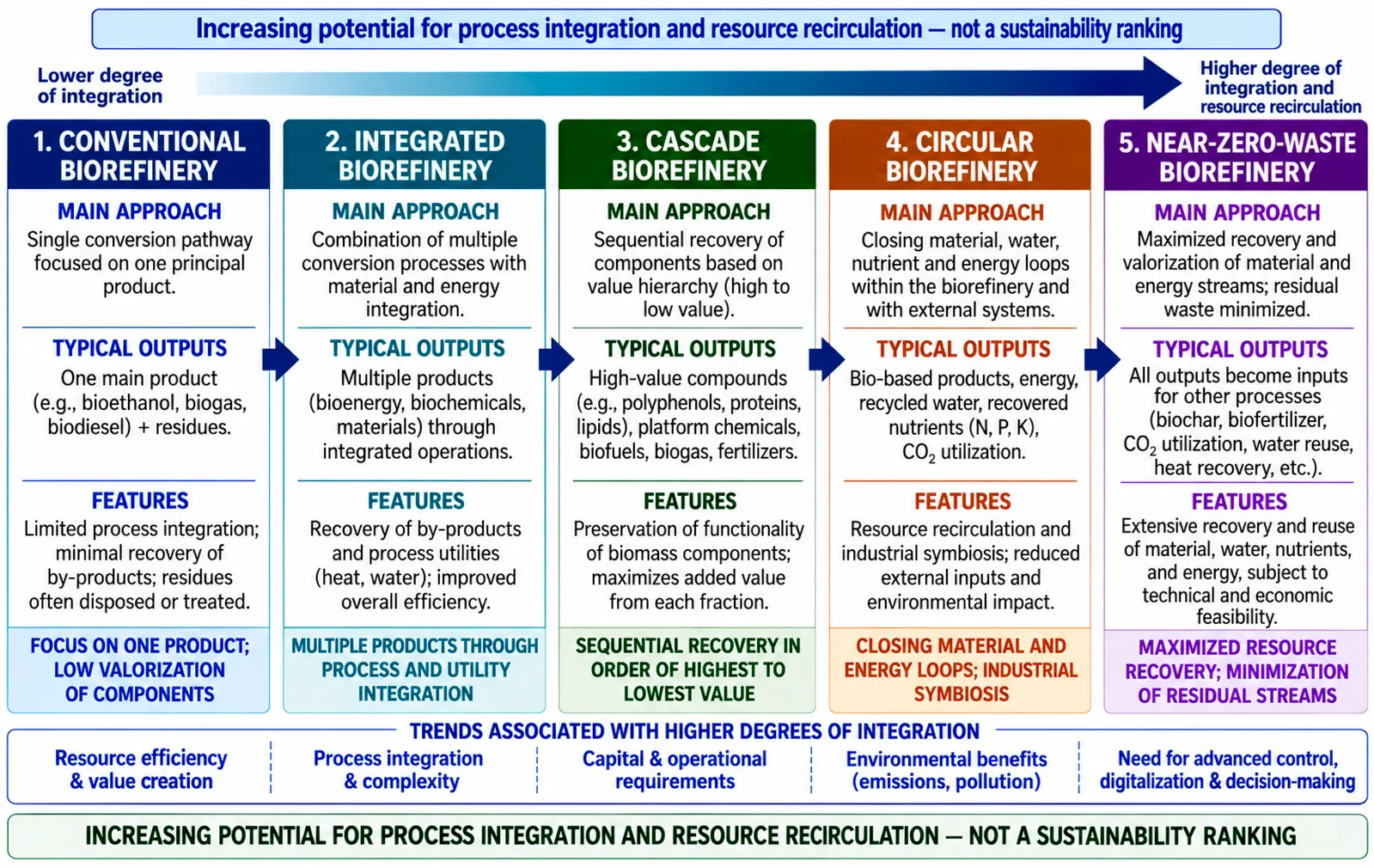
Conceptual comparison of conventional, integrated, cascade, circular, and near-zero-waste biorefinery configurations. The configurations illustrate different degrees and forms of process integration, product diversification, resource recovery, and internal recycling of carbon, nutrients, water, and energy. Their arrangement reflects increasing potential for process integration and resource recirculation but should not be interpreted as a sustainability ranking or as evidence that a more highly integrated configuration is inherently environmentally or economically superior. Additional integration may improve resource utilization while simultaneously increasing energy and water demand, capital investment, separation requirements, and operational complexity. The environmental and economic performance of each configuration must therefore be established through system-level assessment, including LCA and TEA. Zero waste is used here only as an aspirational design principle aimed at eliminating residual streams, whereas practical systems approaching this objective are referred to as near-zero-waste or zero-waste-oriented biorefineries.

Conventional, integrated, cascade, circular, and near-zero-waste biorefineries represent distinct design configurations characterized by different degrees and forms of process integration and resource recovery. As illustrated in Figure 2, these configurations can involve increasing coordination of material and energy flows, product diversification, internal recycling, and recovery of carbon and nutrients. However, increasing integration should not be interpreted as a monotonic improvement in environmental or economic sustainability. Additional fractionation, conversion, separation, and recycling steps may increase capital requirements, energy and water consumption, chemical inputs, downstream-processing demands, and operational complexity [73,74]. Consequently, the sustainability performance of a more highly integrated configuration must be demonstrated through system-level assessment, particularly LCA and TEA, rather than inferred from its degree of integration or resource recovery alone.

Therefore, the distinction among biorefinery configurations should be descriptive rather than hierarchical and should not be based solely on the number of products generated or the nominal degree of resource recovery. Instead, the suitability of each configuration should be evaluated according to the extent to which feedstock fractionation, process integration, and resource recirculation provide demonstrable technical, environmental, and economic benefits under the specific system boundaries and operating conditions considered. This framework provides the basis for the critical comparison developed in the following subsections.

### 4.2. Conventional Biorefineries

Conventional biorefineries are based on a single dominant conversion pathway designed to maximize one principal product, while the remaining biomass fractions are treated as by-products or residual streams. Typical examples include anaerobic digestion, bioethanol production, dark fermentation and biodiesel production from waste cooking oil [13,14,15,39]. Although these technologies are technically mature and have significantly advanced biomass conversion, they typically use food waste as a substrate for producing a single target product, leaving much of its potential for broader resource recovery underutilized.

This approach simplifies process design and facilitates industrial implementation but underutilizes the compositional diversity of food waste. Carbohydrates, proteins, lipids and bioactive compounds are frequently converted into a single energy carrier, limiting both carbon recovery and the economic value extracted from the feedstock. Furthermore, dependence on a single product increases vulnerability to fluctuations in market demand, energy prices and policy incentives, reducing overall process resilience.

### 4.3. Integrated Biorefineries

Integrated biorefineries were developed to overcome the limitations of single-product processing by promoting more comprehensive resource utilization and the generation of diversified product streams. Their defining feature is the functional integration of feedstocks, conversion processes, utilities, and products, allowing material and energy streams to be exchanged among unit operations instead of being managed independently [75]. Despite widespread use of the term, genuine integration remains relatively uncommon. Sequential combinations of fermentation, anaerobic digestion or other conversion processes are frequently described as integrated biorefineries even when material, energy and nutrient exchanges are minimal. Simply transferring residual streams between unit operations does not necessarily improve resource efficiency or constitute effective process integration. Such exchanges provide a net benefit only when the recovered material, energy, or nutrient value outweighs the additional treatment, separation, transport, energy, water, and chemical requirements associated with their reuse; carbon allocation, resource recovery, and utility consumption should therefore be evaluated at the system level [75,76].

Well-integrated systems create synergies by recirculating carbon dioxide, waste heat, water and nutrient-rich residues between processes, thereby improving overall resource efficiency. However, increasing integration also raises capital costs, operational complexity and downstream processing requirements [77,78]. System-level indicators, including carbon recovery, energy efficiency, life-cycle impacts, and techno-economic performance, provide a comprehensive basis for assessing integrated biorefineries and their overall performance beyond product diversity alone.

### 4.4. Cascade Biorefineries

Cascade biorefineries extend process integration by prioritizing biomass utilization according to the value of its individual components, enabling sequential recovery of multiple resource fractions. High-value compounds, such as bioactive molecules, proteins, lipids, or structural polysaccharides, are recovered before the remaining biomass is converted into platform chemicals, biofuels, or biogas, with residual streams further valorized as biofertilizers or soil amendments [30,79]. This strategy generally improves resource efficiency by preserving the functionality of biomass components that would otherwise be lost during direct energy conversion. However, the benefits of sequential fractionation should not be assumed a priori.

Additional extraction and purification steps increase process complexity, energy and water consumption, capital investment and downstream processing costs, while modifying the composition of the remaining biomass and influencing subsequent conversion pathways. For example, protein extraction may reduce ammonia inhibition during anaerobic digestion but also decrease methane yields, whereas sugar recovery enhances biochemical production at the expense of residual energy potential [30,80].

The performance of cascade biorefineries reflects the balance among product value, recovery efficiency, process complexity, and environmental performance, together with the contribution of product diversification to overall system efficiency.

### 4.5. Circular Biorefineries

Circular biorefineries extend cascade processing by aiming not only to recover valuable products but also to recirculate carbon, nutrients, water and energy within the biorefinery and, where feasible, across interconnected industrial systems. Resource recovery may include nutrient recycling through digestate, water reuse, carbon dioxide utilization and integration with renewable energy or industrial symbiosis.

Despite its widespread use, circularity is frequently treated as a qualitative concept without being quantified as a system property. The use of food waste as a feedstock does not inherently ensure circular performance, particularly when conversion relies on large inputs of fossil-derived energy, freshwater, or chemicals. Recent life-cycle assessments demonstrate that circularity is influenced by system boundaries, allocation methods, electricity sources, transport requirements, and assumptions regarding avoided products [7,16,72]. Circular biorefineries should therefore be evaluated using harmonized environmental and resource-efficiency indicators that capture the overall performance of the system beyond the origin of the feedstock.

### 4.6. Near-Zero-Waste and Zero-Waste-Oriented Biorefineries

Near-zero-waste or zero-waste-oriented biorefineries are practical configurations designed to approach the aspirational zero-waste objective through extensive resource integration and the minimization of residual material and energy streams. In food-derived residual-stream biorefineries, this may include converting lignocellulosic residues into carbon-based products, recycling digestate as biofertilizer, recovering nutrients and water from wastewater, utilizing carbon dioxide, and integrating excess heat into other process operations. These interactions resemble industrial ecosystems in which outputs from one process can serve as inputs to another.

In this review, “zero waste” is reserved for the aspirational design concept of eliminating residual streams through complete recovery, reuse, or valorization. Complete zero waste remains practically unattainable because thermodynamic limitations, unavoidable process losses, contamination, product-quality requirements, and economic constraints prevent complete recovery of all material and energy flows. Moreover, maximizing resource recovery may increase energy consumption and processing costs beyond the environmental or economic value of the additional products obtained. Practical systems pursuing this objective are therefore described as near-zero-waste or zero-waste-oriented biorefineries. Their design should seek progressive residual-stream minimization, with the extent of further recovery determined by technical, environmental, and economic feasibility [17,75,80,81,82].

Conventional, integrated, cascade, circular, and near-zero-waste biorefineries differ in their degree and form of process integration, resource recovery, and operational complexity. These configurations should be regarded as alternative and potentially complementary design strategies rather than successive levels of sustainability. Their principal characteristics, potential advantages, and limitations are summarized in Table 3.

### 4.7. Synthesis and Future Perspectives

**Figure 3:**
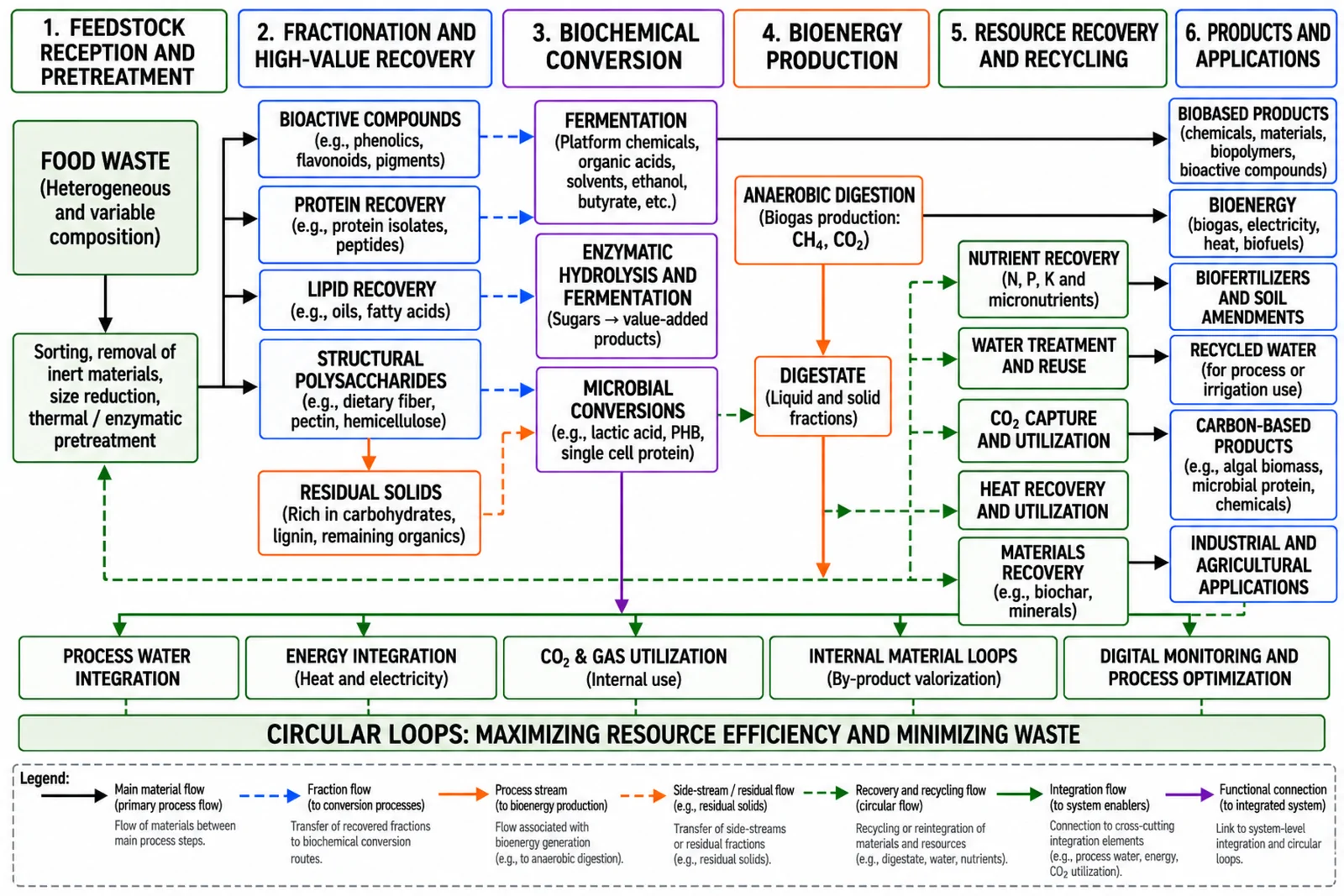
Conceptual framework of an integrated circular food-waste biorefinery. The schematic illustrates a systems-based approach for the comprehensive valorization of food waste through sequential fractionation, biochemical conversion, bioenergy production, and resource recovery. Following pretreatment, high-value compounds are selectively recovered prior to the conversion of the remaining biomass into platform chemicals, biofuels, and biogas. Residual streams are further utilized for nutrient recovery, water recycling, carbon dioxide capture and utilization, heat integration, and material recovery, enabling the production of biofertilizers, recycled water, carbon-based products, and other bio-based materials. Internal recycling of material and energy streams promotes process integration, enhances resource efficiency, and minimizes waste generation, supporting the transition from conventional processing toward circular and near-zero-waste biorefinery systems.

The comparison of conventional, integrated, cascade, circular, and near-zero-waste biorefineries supports considering these configurations as alternative and potentially complementary design strategies, each reflecting different degrees and forms of process integration, resource recovery, and system complexity. Greater integration may increase carbon utilization, product diversification, and internal recycling, but these potential benefits may be offset by additional energy and water consumption, separation requirements, capital investment, and operational complexity. Accordingly, Figure 2 and Figure 3 and Table 3 should be interpreted as conceptual comparisons of system configuration rather than as a hierarchy of sustainability performance. A cascade, circular, or near-zero-waste configuration should be considered environmentally or economically advantageous only when such benefits are demonstrated through appropriate system-level LCA and TEA.

A major limitation of current research is the absence of harmonized criteria for defining and evaluating integrated, circular, and near-zero-waste or zero-waste-oriented biorefineries. As illustrated in Figure 3, future food-waste biorefineries will depend less on individual conversion technologies than on their effective integration within flexible processing systems that combine fractionation, biochemical conversion, bioenergy production and resource recovery. Their performance will rely on advanced process monitoring, modelling, and digital control systems capable of adapting to feedstock variability while optimizing environmental and economic outcomes.

To illustrate the extent to which the system-level indicators proposed above are currently reported in the literature, Table 4 provides a comparative synthesis of representative food-waste and food-processing-side-stream biorefinery studies. The comparison focuses on carbon recovery, resource or energy efficiency, greenhouse-gas performance, water use or recovery, and techno-economic performance. Because these studies differ substantially in feedstock, process configuration, scale, functional unit, system boundaries, allocation procedures, and economic assumptions, the reported values are retained in their original study-specific form and are not normalized or ranked. Missing entries are shown explicitly as not reported (NR).

As shown in Table 4, system-level quantitative reporting remains incomplete and highly heterogeneous across the available literature. Individual studies report selected indicators, including process-level yields or efficiencies, greenhouse-gas performance, carbon recovery, and techno-economic metrics, but these indicators are rarely reported together for the same integrated system. Carbon recovery is explicitly quantified in only a limited subset of the representative studies, while quantitative water-use or water-recovery indicators are absent from the studies summarized here. Resource and energy performance is also expressed using heterogeneous metrics, including extraction efficiency, solubilization, product yield, biogas yield, production rate, and energy-related process requirements. Moreover, differences in functional units, system boundaries, reference scenarios, process scale, and economic assumptions limit direct numerical comparison even when nominally similar indicators are available. The comparison therefore demonstrates both the incomplete reporting and the lack of harmonization of system-level performance indicators across current integrated-biorefinery studies.

Future studies should therefore report, where applicable, a harmonized minimum set of system-level indicators, including carbon recovery, nutrient recovery, net energy or resource efficiency, greenhouse-gas emissions, net water consumption and recovery, and key techno-economic indicators, using clearly defined functional units, system boundaries, reference scenarios, and assessment assumptions. Reporting both the numerical value and its methodological basis would facilitate comparison among alternative configurations and allow the environmental and economic consequences of increasing process integration to be evaluated more consistently.

## 5. Pretreatment Technologies: Unlocking the Valorization Potential of Food Waste

Pretreatment is a critical step in food-waste biorefineries because it determines the accessibility of biomass components, the efficiency of downstream conversion processes and, ultimately, overall resource recovery. Although food waste generally contains readily biodegradable carbohydrates, proteins and lipids, its heterogeneous composition, variable moisture content, structural complexity and the presence of contaminants frequently limit direct conversion. Pretreatment should be regarded not as an isolated unit operation but as an integral component of biorefinery design [94,95].

Recent research has shifted from maximizing biomass deconstruction toward optimizing pretreatment within the context of the entire biorefinery. Severe pretreatments may increase substrate accessibility but also promote inhibitor formation, consume large amounts of energy or chemicals, degrade valuable compounds and increase downstream processing requirements. Pretreatment performance should be evaluated not only by improvements in hydrolysis or conversion yields but also by resource efficiency, environmental impacts, techno-economic feasibility and compatibility with downstream processing [95,96].

No universal pretreatment is suitable for all food wastes. The optimal strategy depends on feedstock characteristics, target products and the intended conversion pathway. Readily degradable substrates may require only mild conditioning, whereas lignocellulosic or highly heterogeneous residues often require more intensive or combined pretreatment strategies to improve fractionation, enzymatic accessibility and overall process efficiency.

### 5.1. Physical Pretreatment Technologies

Physical pretreatments are among the most mature biomass-conditioning strategies used in food-waste biorefineries because they enhance substrate accessibility without introducing chemical reagents [95,97]. No physical pretreatment is universally applicable, as its effectiveness is influenced by feedstock composition, moisture content, downstream conversion requirements, and overall process economics [95,98]. Technology selection should therefore reflect the contribution of pretreatment to overall biorefinery performance, including hydrolysis efficiency, energy demand, scalability, and compatibility with subsequent conversion steps. The principal characteristics of the most widely applied technologies are summarized in Table 5. Particular attention should also be given to scale-up feasibility and energy efficiency, since favorable laboratory performance does not necessarily translate into industrial applicability.

Among commercially available technologies, mechanical size reduction remains the reference pretreatment because of its operational simplicity, scalability and compatibility with anaerobic digestion, fermentation and enzymatic hydrolysis. However, Gallego-García et al. (2023) reported that the benefits of progressive particle-size reduction rapidly diminish below an optimum threshold, whereas electricity consumption continues to increase, making excessive milling difficult to justify at industrial scale [108].

Similar conclusions have been reached in recent food-waste valorization reviews, which indicate that moderate comminution generally provides sufficient improvements in substrate accessibility, while further energy input produces only marginal gains in bioconversion efficiency [65,108]. Current research has shifted from maximizing particle-size reduction toward optimizing particle size according to feedstock properties and the intended conversion process [99,108].

Drying illustrates a different trade-off between process performance and sustainability. Although indispensable for storage, thermochemical conversion, and the extraction of high-value compounds, conventional thermal drying remains one of the most energy-intensive pretreatment operations for wet food waste. Recent reviews therefore question its routine application unless low-cost thermal energy is available through waste-heat recovery or renewable sources. At the same time, hybrid drying technologies and process integration have attracted increasing attention because they can reduce operating costs and greenhouse-gas emissions while preserving product quality. These findings indicate that drying conditions and technologies should be tailored to the requirements of the target product and their role within the overall processing scheme [97].

Greater disagreement exists regarding advanced physical pretreatments. Steam explosion is consistently recognized as one of the most effective technologies for lignocellulosic food-processing residues because it improves fibre disruption and enzymatic accessibility. However, both recent reviews and experimental studies indicate that its advantages decrease considerably for readily biodegradable food waste, where severe treatment may generate furfural, 5-hydroxymethylfurfural and phenolic inhibitors without proportional improvements in conversion efficiency. Likewise, ultrasonication has repeatedly demonstrated enhanced solubilization and extraction of intracellular compounds under laboratory conditions, yet Gallego-García et al. (2023) identified energy demand and reactor scale-up as the principal barriers to broader industrial implementation [108]. Current developments therefore favor combining steam explosion or ultrasonication with biological or mild chemical pretreatments to improve overall process efficiency and facilitate their integration into biorefinery systems [99,108].

Overall, the available evidence indicates that the objective of physical pretreatment is no longer to maximize biomass disruption but to achieve the minimum treatment severity required for efficient downstream conversion. This shift explains the growing preference for feedstock-specific and hybrid pretreatment strategies that balance substrate accessibility, energy consumption, environmental impacts and techno-economic performance instead of optimizing a single processing parameter.

### 5.2. Chemical Pretreatment Technologies

Chemical pretreatments remain the most effective option for processing lignocellulosic food-processing residues because they directly modify biomass structure, increasing cellulose accessibility and facilitating the recovery of fermentable sugars and high-value compounds [95,109]. However, the recent literature has shifted the discussion from pretreatment efficiency alone to the broader balance between biomass deconstruction, chemical consumption, solvent recovery and overall biorefinery sustainability [109,110]. As summarized in Table 6, chemical pretreatments differ markedly in their mechanisms, environmental burdens, technology readiness and suitability for different food-waste streams, indicating that no single approach is universally applicable. Acid and alkali pretreatments continue to represent the industrial benchmark because they combine relatively high technological maturity (TRL 6–8) with consistent improvements in biomass digestibility [95,97].

Acid hydrolysis remains particularly effective for hemicellulose-rich residues, including fruit peels, cereal by-products and brewer’s spent grain, whereas alkaline pretreatment is generally preferred for lignin-rich substrates because it selectively removes lignin while preserving cellulose [97,112]. Nevertheless, recent reviews consistently emphasize that these technologies achieve high conversion efficiencies at the expense of increased reagent consumption, corrosion, wastewater generation and downstream neutralization [95,119]. Moreover, severe acidic conditions promote the formation of furfural, 5-hydroxymethylfurfural and phenolic compounds, whereas alkaline pretreatment requires extensive washing and chemical recovery to control operating costs and environmental impacts.

Accordingly, recent research no longer aims to maximize biomass deconstruction but to reduce chemical loading through milder operating conditions, lower catalyst dosages and integration with enzymatic hydrolysis or biological conversion. Recent reviews on chemical pretreatment similarly conclude that low-liquid and low-chemical strategies reduce inhibitor formation, corrosion and energy demand while maintaining satisfactory conversion efficiencies [119].

The development of ionic liquids (ILs) and deep eutectic solvents (DESs) reflects the transition toward more selective biomass fractionation [120,121]. Both solvent systems exhibit excellent capacity to disrupt lignocellulosic structures under relatively mild conditions, enabling efficient cellulose accessibility while facilitating lignin recovery and the preservation of thermolabile compounds. However, despite outstanding laboratory performance, their industrial deployment remains limited (TRL 4–5). Recent reviews identify solvent cost, viscosity, recycling efficiency, product separation, and long-term solvent stability as the principal barriers to commercialization, while environmental assessments demonstrate that claims of “green” pretreatment cannot be based solely on negligible solvent volatility. Instead, solvent synthesis, recovery efficiency, repeated reuse and end-of-life management frequently dominate both environmental and economic performance [117,120]. Current research has shifted from developing new solvent formulations toward improving solvent recyclability, reducing energy demand during regeneration and integrating pretreatment with downstream fractionation processes.

A common conclusion emerging from the recent literature is that chemical pretreatments should no longer be evaluated as isolated unit operations but as integral components of an integrated biorefinery. Studies published during the last few years consistently show that the highest sugar release does not necessarily produce the best environmental or economic performance once solvent recovery, wastewater treatment, energy consumption and downstream processing are considered. Thus, hybrid strategies combining mild chemical pretreatment with physical or biological methods, together with closed-loop solvent recycling and process integration, are regarded as a promising direction for the development of food-waste biorefineries. Future progress is therefore expected to depend less on developing more aggressive pretreatment chemistries than on improving resource efficiency, solvent circularity and system-level process integration.

### 5.3. Biological Pretreatment Technologies

Biological pretreatments have emerged as an environmentally sustainable alternative to conventional physical and chemical methods because they enhance biomass accessibility under mild operating conditions while minimizing chemical consumption and inhibitor formation. However, their effectiveness is influenced by feedstock characteristics, reaction kinetics, and compatibility with downstream conversion processes. Biological approaches are therefore commonly integrated with thermochemical methods as complementary pretreatment strategies within food-waste biorefineries [122]. Table 7 compares the principal biological pretreatment strategies according to their preferred substrates, advantages, limitations, technology readiness, and current research priorities.

Recent literature indicates that no biological pretreatment is universally superior for food-waste valorization. Instead, fungal, enzymatic and microbial approaches exhibit complementary advantages that reflect differences in substrate composition, reaction selectivity, process duration and industrial maturity [123]. Among biological approaches, fungal pretreatment remains the preferred option for lignocellulosic food-processing residues because white-rot fungi selectively degrade lignin while largely preserving structural carbohydrates. Numerous studies have reported improved enzymatic digestibility and methane production following fungal pretreatment of cereal by-products, fruit residues, and vegetable wastes [123,124]. Nevertheless, recent critical reviews identify prolonged residence time, sensitivity to operating conditions and the risk of carbohydrate losses as the principal limitations preventing large-scale implementation.

Current research focuses on strain selection, microbial consortia, and hybrid pretreatment strategies that combine fungal delignification with mild physical or chemical disruption to shorten processing time while maintaining high selectivity. Enzymatic pretreatment offers greater operational control because hydrolysis is achieved without microbial growth and under well-defined reaction conditions. Recent studies consistently demonstrate that enzyme cocktails outperform individual enzymes for heterogeneous food waste, improving hydrolysis efficiency while preserving thermolabile compounds required for the production of biofuels and high-value biochemicals. However, commercial enzyme formulations remain one of the major contributors to processing costs, and enzyme activity is strongly influenced by substrate composition and the presence of inhibitory compounds. Recent research has shifted toward on-site enzyme production, enzyme immobilization, recycling technologies and enzyme mixtures tailored to specific feedstocks in order to improve economic feasibility [130].

Microbial pretreatment represents the most dynamic area of current research because mixed microbial communities simultaneously hydrolyse complex organic matter and generate valuable intermediates such as volatile fatty acids, hydrogen and soluble carbon sources. Recent investigations demonstrate that controlled microbial pretreatment can substantially accelerate the hydrolysis stage of anaerobic digestion and increase volatile fatty acid production, thereby supporting cascade biorefinery concepts. Nevertheless, maintaining stable microbial communities under variable feedstock conditions remains a significant operational challenge. As a result, synthetic microbial consortia, microbiome engineering and real-time process monitoring have become major research priorities for improving process stability and industrial scalability [122].

Recent evidence indicates that biological pretreatments should not be evaluated solely by improvements in biomass hydrolysis or product yield. Their principal value lies in reducing chemical consumption, operating under mild conditions with low chemical consumption, reducing inhibitor formation, and facilitating integration with downstream biological conversion [95,123]. Hybrid pretreatment strategies that combine mild physical or chemical conditioning with targeted biological processing offer a promising route for food-waste biorefineries by balancing conversion efficiency, operating costs, and environmental sustainability more effectively than individual pretreatment technologies.

### 5.4. Decision Framework for Pretreatment Selection

Selecting an appropriate pretreatment strategy remains one of the most critical decisions in food-waste biorefinery design because no single technology is universally suitable for all feedstocks or product portfolios [95,97]. The comparative analysis presented in Section 4.1, Section 4.2 and Section 4.3 demonstrates that pretreatment performance depends on balancing biomass accessibility with energy demand, chemical consumption, inhibitor formation, environmental impacts and downstream processing requirements. Pretreatment is now considered an enabling technology whose optimization should occur at the biorefinery level, with full consideration of its interactions with downstream processes [95,131].

**Figure 4:**
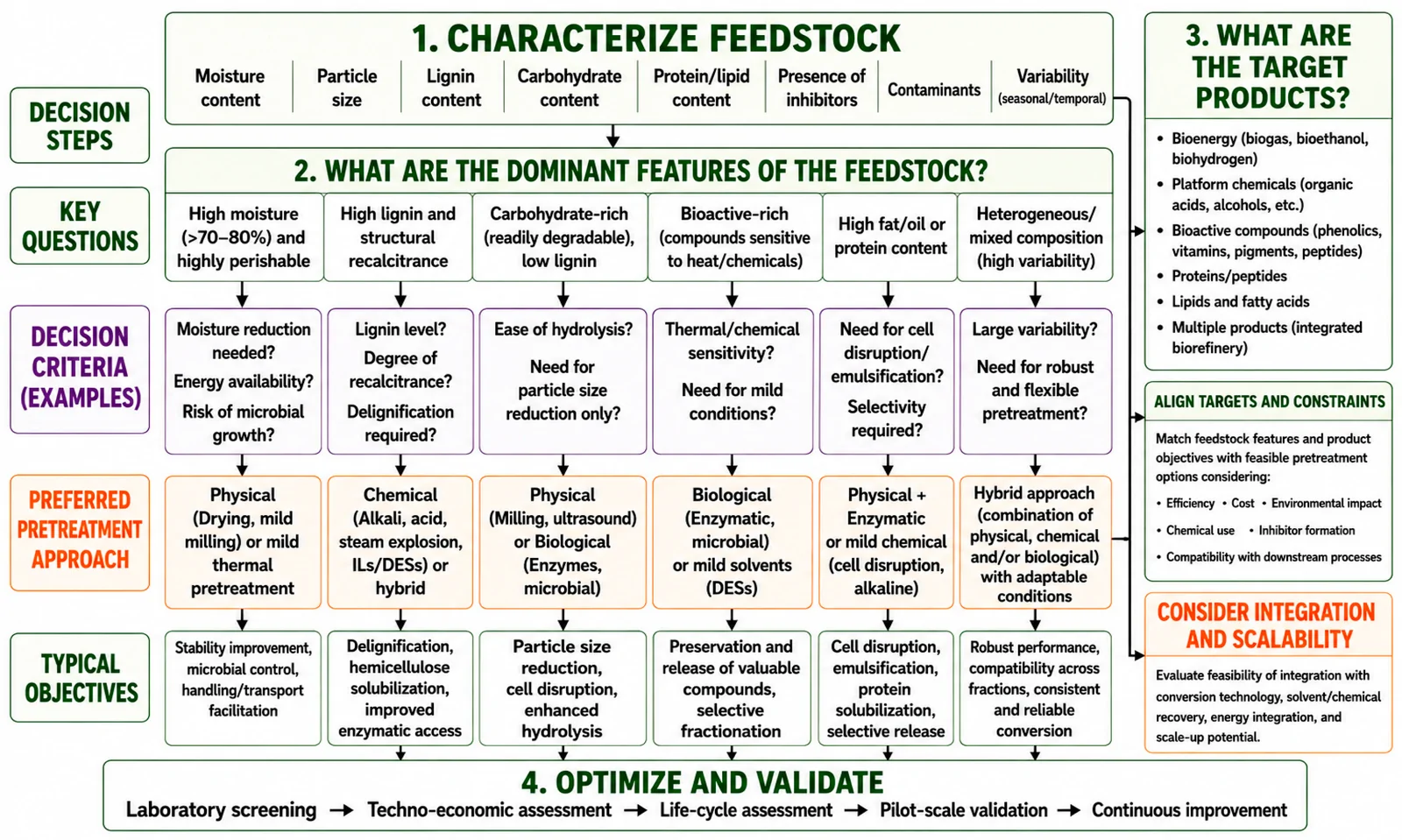
Decision framework for selecting pretreatment technologies in integrated food-waste biorefineries. The framework illustrates a systems-based approach for selecting physical, chemical, biological, or hybrid pretreatment strategies according to feedstock characteristics, including moisture content, lignin concentration, carbohydrate composition, bioactive compound content, and compositional heterogeneity, together with the intended product portfolio. The decision process integrates technical, economic, and environmental considerations, including pretreatment efficiency, energy and chemical requirements, inhibitor formation, process integration, solvent recovery, industrial scalability, and life-cycle sustainability. The framework emphasizes that pretreatment selection should be guided by the optimization of overall biorefinery performance, with biomass deconstruction and product yield considered within the broader context of process efficiency and integration.

Figure 4 proposes a decision framework in which pretreatment selection follows a hierarchical approach guided by feedstock and process requirements. Feedstock characterization constitutes the first decision step because moisture content, lignin concentration, carbohydrate composition, lipid and protein content, inhibitory compounds and compositional variability largely determine the feasibility of subsequent processing. These characteristics should then be evaluated together with the intended product portfolio, since pretreatment requirements differ substantially for bioenergy production, platform chemicals, bioactive compounds or integrated multiproduct biorefineries. This approach recognizes that the same food waste may require different pretreatment strategies depending on the desired valorization pathway.

Recent literature consistently demonstrate a transition from technology-oriented to feedstock-oriented pretreatment selection. Current research focuses on determining the minimum pretreatment severity required to ensure efficient downstream conversion, while avoiding unnecessary biomass disruption [95,97]. This shift explains the growing interest in hybrid pretreatment strategies, where physical, chemical and biological methods are combined to exploit complementary mechanisms while reducing energy consumption, chemical requirements and inhibitor formation.

At the same time, TEA and LCA have become indispensable tools for pretreatment selection, as laboratory-scale improvements frequently fail to translate into industrial benefits when solvent recovery, wastewater treatment, electricity demand and process integration are considered [87,132]. These findings indicate that pretreatment efficiency should be assessed using system-level performance indicators that account for biomass conversion within the broader context of overall process performance.

Future food-waste biorefineries are expected to adopt adaptive pretreatment strategies capable of responding to continuous variations in feedstock composition and product demand. Advances in sensor technologies, process modelling, digital twins, and artificial intelligence are being explored to support real-time optimization of pretreatment conditions, while pilot-scale validation and technology readiness assessment have become essential for bridging the gap between laboratory research and industrial implementation. The future development of pretreatment technologies is likely to depend less on the discovery of new processing methods than on their intelligent integration within flexible, resource-efficient and industrially scalable biorefinery systems [87,97].

## 6. Food Waste Conversion Pathways

Food waste is recognized as a renewable carbon resource with substantial valorization potential. Its heterogeneous composition, including carbohydrates, proteins, lipids, fibers, minerals, and bioactive compounds, enables the production of fuels, platform chemicals, biomaterials, food ingredients, and feed products [6,70]. So, the objective of modern food-waste biorefineries has shifted from waste treatment to maximizing resource valorization through integrated conversion pathways [71,95].

The diversity of food waste also complicates technology selection. Variations in composition, moisture content, biodegradability, and contamination mean that no single conversion pathway is universally applicable. Instead, technology selection should balance feedstock characteristics with target products, technology readiness, market demand, environmental performance, and process economics [6,10,19].

Current valorization strategies can be broadly classified into three product groups: biofuels, platform chemicals, and high-value bioproducts. While biofuels remain the most technologically mature option, platform chemicals and specialty bioproducts generally provide greater added value by preserving biomass functionality [7,32,62]. However, higher product value is frequently accompanied by lower technology readiness, more demanding downstream processing, and greater commercialization challenges.

This chapter critically compares the major food-waste conversion pathways, emphasizing their technological maturity, advantages, limitations, and suitability for integration within biorefinery systems. Particular attention is given to the trade-offs between product value, process complexity, scalability, and industrial implementation.

### 6.1. Biofuels

Biofuels remain the most technologically mature products obtained from food waste and continue to play a key role in reducing fossil fuel dependence while diverting biodegradable waste from landfill [7,10]. Current evidence indicates that energy recovery is no longer considered the primary objective of food-waste valorization, with greater emphasis placed on higher-value resource recovery pathways [70,71]. Instead, within modern biorefinery concepts, biofuels are preferentially produced after recovering higher-value compounds, allowing residual carbon to be converted into renewable energy through cascade processing [62,70,71]. This transition reflects a broader shift from maximizing biomass conversion toward maximizing resource value and overall carbon utilization. The principal characteristics of the major biofuel pathways are summarized in Table 8.

Recent comparative studies demonstrate that bioethanol and biogas are currently the only food-waste biofuel pathways with high technological maturity and established industrial implementation, although they differ substantially in feedstock requirements and process flexibility [7,64,65]. Bioethanol is best suited to homogeneous carbohydrate-rich residues, including bakery waste, confectionery by-products and starchy food-processing residues, where mild pretreatment and efficient fermentation enable ethanol yields approaching those obtained from conventional feedstocks. In contrast, anaerobic digestion tolerates highly heterogeneous mixtures containing carbohydrates, proteins and lipids, making it the preferred option for municipal food waste and residual biomass [65,70]. Several reviews emphasize that the main constraints affecting both technologies are primarily economic, despite their demonstrated technological feasibility. Bioethanol production remains constrained by pretreatment and distillation costs, whereas the relatively low value of biogas and the irreversible conversion of organic carbon into methane reduce its attractiveness when higher-value products can first be recovered [64,70,73]. Current investigations position anaerobic digestion primarily as the final stage of cascade biorefineries, where it complements upstream valorization processes and supports the recovery of residual organic matter [70,73].

Biohydrogen and biodiesel illustrate two contrasting approaches that remain less widely implemented despite considerable research activity. Biohydrogen has attracted growing interest because of its potential contribution to low-carbon energy systems and its integration with volatile fatty acid production or subsequent methane generation [29,135,137]. However, comparative reviews consistently identify low hydrogen productivity, process instability and costly downstream gas purification as the principal barriers preventing commercial deployment, explaining why most systems remain at laboratory or pilot scale. Biodiesel derived directly from waste cooking oil is considerably more mature and economically attractive because it exploits an existing lipid-rich waste stream and relies on well-established transesterification technology [138,139]. By contrast, microbial lipid production from food-waste hydrolysates remains technologically promising but commercially constrained by fermentation costs, biomass harvesting, lipid extraction and solvent recovery. These findings indicate that feedstock availability and downstream processing currently exert a greater influence on industrial feasibility than biological conversion efficiency alone [8,70,127].

Technology readiness varies markedly among the four pathways. Anaerobic digestion and biodiesel production from waste cooking oil have already reached commercial maturity (TRL 8–9), while food-waste bioethanol is approaching full industrial implementation for selected feedstocks [29,64,65]. In contrast, biohydrogen and biodiesel based on microbial lipids remain predominantly at laboratory or pilot scale (TRL 3–5), despite continuous improvements in metabolic engineering, reactor design and process optimization [29,127,137]. This disparity demonstrates that successful scale-up depends not only on biological performance but also on feedstock logistics, product purification, market demand and overall process economics. Recent techno-economic analyses therefore conclude that laboratory yields alone provide a poor predictor of commercial feasibility [34,77].

Current evidence indicates that the future role of biofuels within food-waste biorefineries will depend less on maximizing energy production than on their integration with higher-value conversion pathways. Biofuels complement platform chemicals and specialty bioproducts by enabling the conversion of residual biomass into renewable energy following the recovery of valuable compounds. This integrated approach improves carbon utilization, diversifies product portfolios, and enhances the economic resilience of food-waste biorefineries, reinforcing the transition from single-product biofuel facilities toward multiproduct biorefinery systems.

### 6.2. Platform Chemicals

Platform chemicals represent one of the most promising routes for increasing the economic value of food waste because they retain biogenic carbon within the production cycle while supplying renewable intermediates for the chemical, polymer and pharmaceutical industries [24,62,70]. Unlike biofuels, whose value is primarily associated with energy recovery, platform chemicals generate substantially greater added value from the same carbohydrate-rich feedstocks. Recent research has shifted from maximizing ethanol production toward developing flexible fermentation platforms capable of directing food-waste-derived sugars to higher-value organic acids [24,99,127]. The main characteristics of the principal platform chemicals produced from food waste are summarized in Table 9.

Comparative studies indicate that lactic acid currently represents the most advanced platform chemical for food-waste valorization because it combines high fermentation efficiency, expanding demand driven by polylactic acid (PLA) production and the highest technology readiness (TRL 8–9) [149]. Recent developments therefore focus less on improving microbial productivity than on reducing downstream purification costs through membrane separation, electrodialysis and integrated recovery systems [149]. In contrast, succinic acid remains one of the most versatile renewable platform molecules because of its broad chemical applications and the possibility of incorporating carbon dioxide during fermentation [145,150]. Nevertheless, despite significant advances in metabolic engineering and fermentation performance, its commercialization continues to be constrained by costly product recovery and purification, which frequently dominate overall production costs [145,151].

Citric acid illustrates a different stage of technological development. Unlike lactic and succinic acids, industrial fermentation is already highly optimized, and current research focuses on replacing refined sugars with food-waste-derived substrates while maintaining product quality and process efficiency [147,148]. Further improvements in competitiveness depend not only on fermentation performance but also on reducing feedstock handling costs, ensuring consistent substrate quality, and optimizing downstream processing. This distinguishes citric acid from less mature platform–chemical pathways, where process intensification and metabolic engineering remain particularly important for improving productivity and facilitating industrial scale-up [147,148].

Downstream processing represents a common bottleneck in the fermentative production of platform chemicals. Separation, purification and product recovery can account for a substantial fraction of total production costs and, for some organic acids, may outweigh further improvements in fermentation efficiency [145,146]. Current research integrates fermentation with membrane technologies, electrodialysis, reactive extraction, adsorption, and crystallization to improve product recovery and reduce downstream costs [149,152,153]. Continuous processing, in situ product recovery, and low-pH fermentation are also being investigated to reduce chemical consumption, salt formation, and wastewater generation [152].

Further improvements in the economic and environmental performance of organic-acid production are expected to rely on effective process integration, together with continued advances in fermentation yield and productivity [149,152]. The literature demonstrates that selecting a platform chemical should not be based solely on fermentation performance or product yield. Feedstock composition, downstream recovery requirements, market demand and technology readiness collectively determine industrial feasibility. Thus, future food-waste biorefineries are expected to employ flexible fermentation platforms capable of redirecting carbohydrate-rich substrates toward different organic acids according to economic conditions, reinforcing the transition from single-product fermentation processes to adaptive multiproduct biorefineries.

### 6.3. High-Value Bioproducts

High-value bioproducts represent an advanced route for food-waste valorization because they preserve specific functional properties of biomass while supplying products for the food, pharmaceutical, cosmetic, packaging, agricultural and biotechnology sectors [94,154]. Unlike biofuels, which primarily recover energy, or platform chemicals, which generate bulk intermediates, these products derive their value from specific biological and functional properties, including catalytic activity, biodegradability, nutritional quality and bioactivity [94,155]. High-value bioproducts can provide greater economic value while retaining biomass functionality, although their production generally requires more selective processing, efficient downstream recovery, high product purity and stringent quality control [154,155]. The principal characteristics of the major high-value bioproducts derived from food waste are summarized in Table 10.

Current research demonstrates two complementary valorization strategies. The first relies on microbial bioconversion, where food waste serves as a fermentation substrate for producing industrial enzymes, polyhydroxyalkanoates (PHAs), biosurfactants and single-cell proteins (SCPs). These products benefit from advances in metabolic engineering, synthetic biology and process optimization, enabling the conversion of heterogeneous food waste into materials and biochemicals with substantially higher market value than conventional biofuels [127,156]. However, despite continuous improvements in microbial productivity, downstream processing remains a major challenge to the commercial-scale implementation of microbial food-waste valorization processes. Product recovery, separation and purification can contribute substantially to overall production costs, particularly for products requiring high purity or intracellular recovery, such as PHAs, thereby limiting industrial competitiveness despite promising laboratory-scale results [127,159].

The second strategy focuses on the recovery of naturally occurring compounds, including nutraceuticals and natural pigments, which are extracted directly from food-processing residues before biological conversion. This approach is fully consistent with cascade biorefinery principles because it preserves structurally complex molecules that would otherwise be degraded during fermentation or energy recovery. Recent advances in green extraction technologies, enzyme-assisted extraction, natural deep eutectic solvents, membrane separation and encapsulation have improved extraction efficiency while reducing solvent consumption and environmental impacts [172,173,174]. Commercial feasibility is determined by extraction yield, compound stability, bioavailability, product standardization, and compliance with stringent regulatory requirements. Feedstock variability remains an additional challenge because fluctuations in raw-material composition directly influence extract quality and market value [174,175]. Food and feed applications provide a third important valorization route by enabling the recovery and utilization of the broader nutrient content present in food waste. Single-cell proteins and animal feed production efficiently convert carbohydrates and nitrogen into protein-rich biomass while supporting circular bioeconomy objectives through nutrient recycling [176,177]. Compared with conventional protein production, microbial biomass and insect-based systems require substantially fewer land and water resources and generate lower greenhouse-gas emissions [171,176]. However, their large-scale implementation is strongly influenced by feedstock safety, traceability, contaminant control and regulatory acceptance, in addition to process performance [171,177]. Industrial food-processing by-products currently offer considerably greater commercialization potential than heterogeneous post-consumer food waste, particularly for feed applications subject to strict legislative requirements [177,178].

Although high-value bioproducts may offer greater economic potential than biofuels, their industrial maturity differs considerably. Established fermentation-based processes, including the production of several industrial enzymes, have reached relatively high levels of technological maturity, whereas emerging food-waste valorization routes for PHAs, biosurfactants, single-cell proteins, nutraceuticals, and natural pigments remain at different stages of laboratory, pilot, demonstration, or early commercial development [179,180]. These differences demonstrate that market value alone is not a reliable indicator of industrial feasibility. Successful implementation depends equally on feedstock availability and consistency, process scalability, downstream recovery and purification, regulatory compliance, and market conditions, highlighting the importance of evaluating technology readiness alongside product performance [5,180].

Current evidence suggests that high-value bioproducts may become major economic drivers of future food-waste biorefineries. Integrated facilities are expected to adopt multiproduct configurations combining the recovery of bioactive compounds with microbial production of specialty biochemicals, followed by the conversion of residual biomass into platform chemicals or bioenergy. Such cascade strategies can maximize carbon utilization, diversify revenue streams, and improve process resilience, thereby supporting the transition from waste-treatment facilities to flexible multiproduct biorefineries.

### 6.4. Integrated Perspective on Product Selection: A Critical Synthesis

The diversity of products that can be obtained from food waste is one of the principal strengths of the biorefinery concept but also one of its greatest design challenges. Although numerous studies continue to optimize individual conversion technologies, industrial implementation is primarily determined by the selection of an appropriate product portfolio for each feedstock, together with the efficient integration of the corresponding conversion pathways. Thus, food-waste biorefineries are progressively evolving from technology-oriented systems toward product-oriented platforms that integrate feedstock characteristics, market demand, technology readiness, environmental performance and process economics into a unified decision-making framework [5,70].

This transition has reinforced the importance of cascade valorization, in which biomass can be sequentially allocated to products according to their economic value and structural complexity. Current biorefinery concepts integrate the recovery of valuable compounds and the production of biobased products with subsequent resource and energy recovery from residual streams [70]. Such integrated strategies can improve resource utilization, diversify product portfolios and enhance the economic potential of food-waste biorefineries. Thus, carbon allocation should be considered as a strategic design criterion within integrated multiproduct biorefinery systems.

Recent findings also demonstrate that product selection should be based on four complementary criteria: feedstock suitability, product value, technology readiness and system-level performance [5,70]. Feedstock composition determines the technical feasibility of individual conversion pathways, whereas product value alone rarely guarantees commercial success. Technology readiness defines the likelihood of industrial implementation, while TEA and LCA provide complementary information on profitability and environmental sustainability [70,181]. Collectively, these criteria demonstrate that maximizing a single performance indicator, such as product yield or market price, seldom leads to the optimal biorefinery configuration. Instead, successful industrial systems require balanced evaluation of technical, economic and environmental trade-offs [5,181].

**Figure 5:**
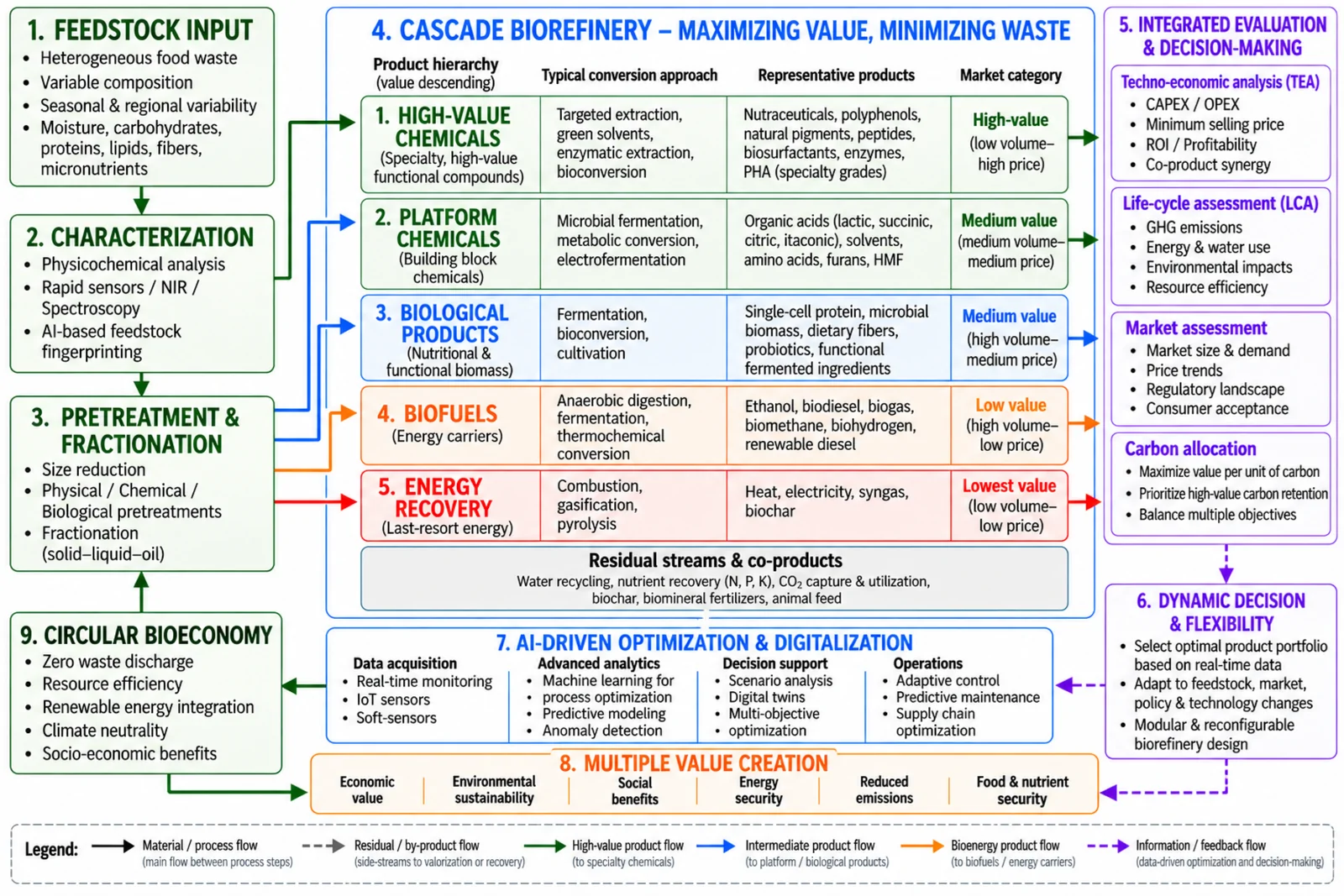
Conceptual framework for product-oriented cascade valorization in food waste biorefineries, illustrating the sequential allocation of biomass toward high-value bioproducts, platform chemicals, biofuels, and residual energy recovery, supported by TEA, LCA, artificial intelligence (AI), and circular bioeconomy principles.

Figure 5 summarizes this product-oriented decision framework by illustrating the sequential allocation of food waste toward high-value bioproducts, platform chemicals, biofuels, and residual energy recovery. The framework also highlights the increasing role of TEA, LCA, and circular bioeconomy principles in guiding technology selection. These approaches are integrated within a unified assessment framework to identify product portfolios that enhance resource efficiency while maintaining industrial feasibility. Future food-waste biorefineries are expected to operate as flexible manufacturing systems capable of adapting to seasonal variations in feedstock composition, fluctuations in product markets and evolving sustainability requirements [182,183]. Recent advances in artificial intelligence, digital twins, soft sensors and predictive process control offer new opportunities to optimize feedstock allocation, fermentation, extraction and downstream processing in real time [180,183]. Although their industrial implementation in biorefineries remains limited, these technologies are expected to play an important role in supporting adaptive and integrated biorefineries capable of responding dynamically to changing operating conditions [182,183].

There is no universally optimal conversion pathway exists for food waste. The most sustainable biorefinery is not necessarily the one producing the highest-value product or achieving the highest conversion yield, but the one that combines appropriate feedstock selection, product diversification, technology readiness and efficient process integration. This systems perspective represents the foundation of next-generation food-waste biorefineries and provides the rationale for evaluating the technological maturity of the major conversion pathways discussed throughout this chapter.

### 6.5. Technology Readiness of Food-Waste Conversion Pathways: Bridging Laboratory Concepts and Industrial Reality

The rapid expansion of food-waste valorization research has generated numerous technologies capable of producing biofuels, platform chemicals and high-value bioproducts under laboratory conditions. However, successful laboratory performance does not necessarily translate into industrial implementation. High product yields obtained under controlled experimental conditions often overlook critical aspects such as feedstock logistics, process robustness, downstream purification, regulatory compliance, market demand and production costs. Technology readiness has become an essential criterion for distinguishing promising laboratory concepts from technologies capable of supporting commercially viable food-waste biorefineries.

Technology readiness level (TRL) provides a standardized framework for assessing the technological maturity of a specific conversion technology from early laboratory research through validation, demonstration, and operational deployment. TRL should be distinguished from broader industrial feasibility: a high TRL indicates that a technology has reached an advanced stage of technical development and demonstration, but does not by itself establish economic viability, environmental superiority, resource efficiency, or suitability for a particular biorefinery context. These dimensions require complementary assessment through TEA, LCA, and other system-level indicators. The interpretation of TRL adopted throughout this review is summarized in Table 11.

Based on this framework, the food-waste conversion pathways discussed throughout this chapter exhibit markedly different levels of technological maturity (Table 12). Based on the application-specific evidence reviewed here, technological maturity varies substantially among food-derived residual-stream conversion pathways and should be assessed for the specific feedstock–process–product configuration rather than inferred from the general maturity of the underlying conversion technology.

Table 12 summarizes the indicative maturity levels represented by selected food-derived residual-stream applications reported in the reviewed literature. These values should be interpreted at the level of the specific feedstock–process–product configuration rather than as intrinsic maturity levels of the corresponding product classes. For example, anaerobic digestion of food waste and biodiesel production from waste cooking oil include applications demonstrated at commercial scale, whereas several food-waste-derived PHA, biosurfactant, and specialty-product pathways remain represented predominantly by laboratory, pilot, or early demonstration studies. Similarly, the maturity of bioethanol, organic-acid, enzyme, single-cell-protein, nutraceutical, and pigment production depends on the specific residual feedstock, process configuration, downstream-processing train, product specification, and operational environment documented in the supporting studies. The broad ranges shown in Table 12 therefore reflect heterogeneity among the cited applications and should not be interpreted as evidence that every technology producing the listed product has reached the upper end of the indicated range.

Across the application-specific evidence reviewed here, several higher-value bioproduct pathways remain at laboratory, pilot, or demonstration scale, whereas some established energy-recovery and fuel-production routes have been demonstrated at larger operational scales. This pattern should not be interpreted as an intrinsic relationship between product value and TRL, because technological maturity depends on the complete feedstock–process–product configuration and its operational environment.

High-value bioproducts generally offer substantially greater economic returns than biofuels, but they also require more complex processing, stricter quality control and more demanding regulatory approval. Selecting the optimal conversion pathway should not rely solely on product value or laboratory productivity but should simultaneously consider technology readiness, downstream processing requirements, market demand and overall process economics. Recent research has therefore shifted from optimizing individual conversion technologies toward accelerating their industrial deployment. Pilot-scale validation, modular biorefinery design, process intensification, continuous operation, advanced separation technologies, digital monitoring, and artificial intelligence are key factors in bridging the gap between laboratory innovation and commercial implementation [70,180,183,184,185,186].

**Table 12:** Application-specific technology-readiness evidence for selected food-derived residual-stream conversion pathways.

Specific Conversion Pathway/Application	Indicative EC-TRL Interpretation	Directly Documented Scale/Status	Evidence Basis	Major Barriers to Industrial Implementation	Application-Specific Evidence
Bioethanol from food waste	Not assigned from available application-specific evidence	Laboratory to developing scale; scale varies among reviewed configurations	The food-waste-specific literature documents technical development, but the cited evidence does not directly establish a defensible application-specific EC-TRL interval.	Feedstock variability; pretreatment; inhibitors; distillation and energy demand	[187]
Anaerobic digestion of food waste	High maturity; exact TRL not assigned from cited evidence	Full-scale/commercial implementation is reported for food-waste digestion systems	Maturity is substantially higher than for emerging biochemical routes, but the cited sources are not used to infer a precise EC-TRL solely from generic anaerobic-digestion maturity.	Digestate management; biomethane upgrading; feedstock logistics; contamination	[65,70,188]
Biohydrogen from food waste	Not assigned from available application-specific evidence	Predominantly laboratory–pilot development	Food-waste-specific studies/reviews document emerging hydrogen production and scale-up constraints, without directly supporting a precise EC-TRL interval.	Low productivity; gas purification; process stability; scale-up	[135,136,189]
Biodiesel from waste cooking oil	High maturity; exact TRL not assigned from cited evidence	Industrial/commercial processing is reported for waste-cooking-oil routes	Waste cooking oil is a specific residual feedstock with mature biodiesel processing, but a numerical EC-TRL is not inferred solely from review-level evidence.	Feedstock quality and availability; pretreatment; catalyst recovery; glycerol valorization	[141,190,191]
L-lactic acid from food waste	Not assigned from available application-specific evidence	Experimental process validation	Primary food-waste-specific evidence demonstrates optically pure L-lactic-acid production, but does not by itself establish pilot, demonstration, or commercial EC-TRL.	Downstream purification; optical purity; inhibitors; substrate variability	[192]
Succinic acid from municipal food waste/candy-factory waste	TRL 3–4	Laboratory-scale integrated process validation	Primary studies demonstrate integrated pretreatment/hydrolysis/fermentation and fermentation–recovery configurations using defined food-derived residual streams; industrial operation is not demonstrated.	Product recovery; purification; fermentation robustness; scale-up	[193,194]
Citric acid from food-processing residual streams	Not assigned from available application-specific evidence	Laboratory/fermentation studies represented in reviewed literature	Available cited evidence does not directly establish an application-specific EC-TRL for food-residual-derived citric acid.	Feedstock standardization; fermentation control; recovery; competition with conventional production	[148,195]
Industrial enzymes from food/agro-industrial residual streams	Not assigned because category is heterogeneous	Laboratory to more advanced applications, depending on enzyme and process	Maturity cannot be assigned generically across enzymes because feedstock, microorganism, fermentation mode, purification, and operational scale differ substantially.	Recovery and formulation; variability; purification; production cost	[156,196]
PHA from food waste/food-waste-derived intermediates	TRL 5–6	Pilot-scale validation	Food-waste-derived PHA has been demonstrated at pilot scale; the assignment is limited to applications for which pilot-scale operation is documented and is not generalized to PHA production as a whole.	Polymer recovery; process economics; feedstock/VFA consistency; product quality	[88,160,161]
Biosurfactants from food/agro-industrial residual streams	Not assigned from available application-specific evidence	Predominantly laboratory-scale development	The cited evidence supports production from food-derived residual substrates but does not directly justify a single EC-TRL interval across heterogeneous biosurfactant processes.	Productivity; downstream recovery; purification; reproducibility; scale-up	[162,163,197,198]
Single-cell proteins from food-processing residual streams	Not assigned from available application-specific evidence	Laboratory to developing/pilot applications	Food-derived residual-stream SCP is documented, but the reviewed evidence does not directly support a generic commercial-level EC-TRL assignment.	Safety and regulation; substrate variability; nucleic-acid reduction; drying	[165,166,176,199]
Bioactive/nutraceutical compound recovery from agri-food residual streams	Not assigned because category is heterogeneous	Predominantly laboratory–pilot development	Extraction and recovery maturity is compound-, feedstock-, and process-specific; review-level evidence is insufficient for a generic numerical EC-TRL.	Extraction scale-up; solvent/energy demand; standardization; regulation	[174,200,201]
Natural-pigment recovery from food-processing residual streams	Not assigned because category is heterogeneous	Predominantly laboratory-scale development	Maturity varies with pigment, feedstock, extraction technology, stabilization, purity specification, and intended application; no generic EC-TRL is assigned.	Stability; extraction efficiency; purification; quality consistency	[169,173]
Processed former foodstuffs/eligible food-processing side streams used as animal-feed ingredients	High maturity; exact TRL not assigned from cited evidence	Established feed use/commercial processing for eligible streams	The assessment is restricted to legally eligible, processed former foodstuffs and defined side streams; it is not generalized to untreated post-consumer food waste.	Feed legislation; traceability; contaminant control; nutritional consistency; logistics	[177,201,202]

Note: TRL = technology readiness level. TRL interpretation follows the European Commission framework [184] and is applied only where the cited evidence permits maturity to be related to a specific feedstock–process–product configuration and documented operational scale. Where the available application-specific evidence does not support a defensible numerical EC-TRL, no numerical value is assigned. Approximate TRL values (≈) represent conservative mappings from directly documented experimental or pilot-scale evidence and should not be interpreted as generic maturity ratings for an entire product class. Commercial maturity of the same product produced from conventional feedstocks was not used as evidence of equivalent maturity for food-derived residual-stream production.

These developments are expected to complement established industrial technologies while facilitating the progressive scale-up and technological maturation of emerging high-value conversion pathways. Technology readiness provides a practical framework for evaluating the industrial potential of food-waste valorization technologies. The evidence reviewed throughout this chapter demonstrates that no single conversion pathway is universally preferable. Successful food-waste biorefineries are therefore likely to combine technologies operating at different TRLs, integrating commercially established processes with emerging high-value products according to feedstock characteristics, market opportunities, environmental performance, and regional priorities. Such an adaptive strategy can facilitate technology selection and investment decision-making while supporting continuous innovation and the transition from laboratory concepts to economically and environmentally sustainable industrial biorefineries [70,181].

## 7. Designing Integrated Food-Waste Biorefineries

The successful implementation of food-waste biorefineries depends not only on the availability of efficient conversion technologies but also on their integration into coherent process configurations capable of enhancing resource utilization [70,99]. As demonstrated in the previous chapters, food waste can be converted into a wide range of biofuels, platform chemicals and high-value bioproducts. However, these products cannot be considered independently because they compete for the same biomass fractions, while their technical feasibility and economic performance are strongly influenced by feedstock composition, processing sequence and interactions among individual unit operations. The design of integrated biorefineries has emerged as a key challenge in translating laboratory-scale technologies into economically viable industrial systems [70].

Recent research has focused on developing feedstock-oriented biorefinery configurations that integrate fractionation, biochemical conversion, bioenergy production, and resource recovery within a unified system. In this review, feedstock-oriented design is therefore understood more broadly than composition-driven process selection. In addition to biochemical composition, biodegradability, contamination, and physical characteristics, it should account for feedstock provenance, regulatory status, current market or societal function, competing uses, and the counterfactual consequences of redirecting the stream to a new process. These configurations allocate biomass according to component functionality and product value, while enabling the recovery of nutrients, water, and energy from residual streams. This approach improves carbon utilization, diversifies product portfolios, and enhances process resilience, thereby supporting the transition from waste management to circular resource management [70,88].

Despite this progress, no universal biorefinery configuration is suitable for all food-waste streams. Carbohydrate-rich, protein-rich, lipid-rich and heterogeneous municipal food wastes differ substantially in composition, biodegradability and contamination profiles, requiring different process sequences and integration strategies. Moreover, the appropriate configuration depends not only on feedstock composition and physicochemical characteristics, but also on technology readiness, market demand, techno-economic performance, environmental constraints, regulatory status, and the existing or counterfactual use of the feedstock. For industrial side streams with established food, feed, fertilizer, or material applications, pathway selection should explicitly consider the function that would be displaced by diversion to the biorefinery and the environmental and economic consequences of providing an alternative substitute. These considerations highlight the need for a systems engineering approach to biorefinery design, in which individual conversion technologies are selected and integrated according to their contribution to overall system performance.

Representative integrated biorefinery scenarios are proposed for the major categories of food waste to illustrate how feedstock characteristics determine process configuration, product allocation, and resource recovery strategies. Particular attention is given to the integration of pretreatment, fractionation, biological conversion, bioenergy production, and circular resource recovery, together with the opportunities and limitations associated with each configuration. The analysis provides a conceptual framework for developing flexible and adaptive food-waste biorefineries, recognizing that optimal design is context-dependent and must accommodate variations in feedstock composition, technological maturity, and industrial requirements.

### 7.1. Carbohydrate-Rich Food Waste Biorefinery

Carbohydrate-rich food wastes, including fruit and vegetable residues, bakery waste, cereal by-products and potato-processing residues, are among the most suitable feedstocks for integrated biorefineries because they contain readily biodegradable carbohydrates together with valuable secondary metabolites such as polyphenols, pigments and pectin [30,198]. Their relatively high fermentability enables the production of a broad spectrum of products, while the presence of extractable bioactive compounds creates opportunities for cascade valorization before energy recovery [25,30]. Recent research has explored selective fractionation and sequential processing strategies, allowing biomass components to be allocated according to their functional and economic value [25].

**Figure 6:**
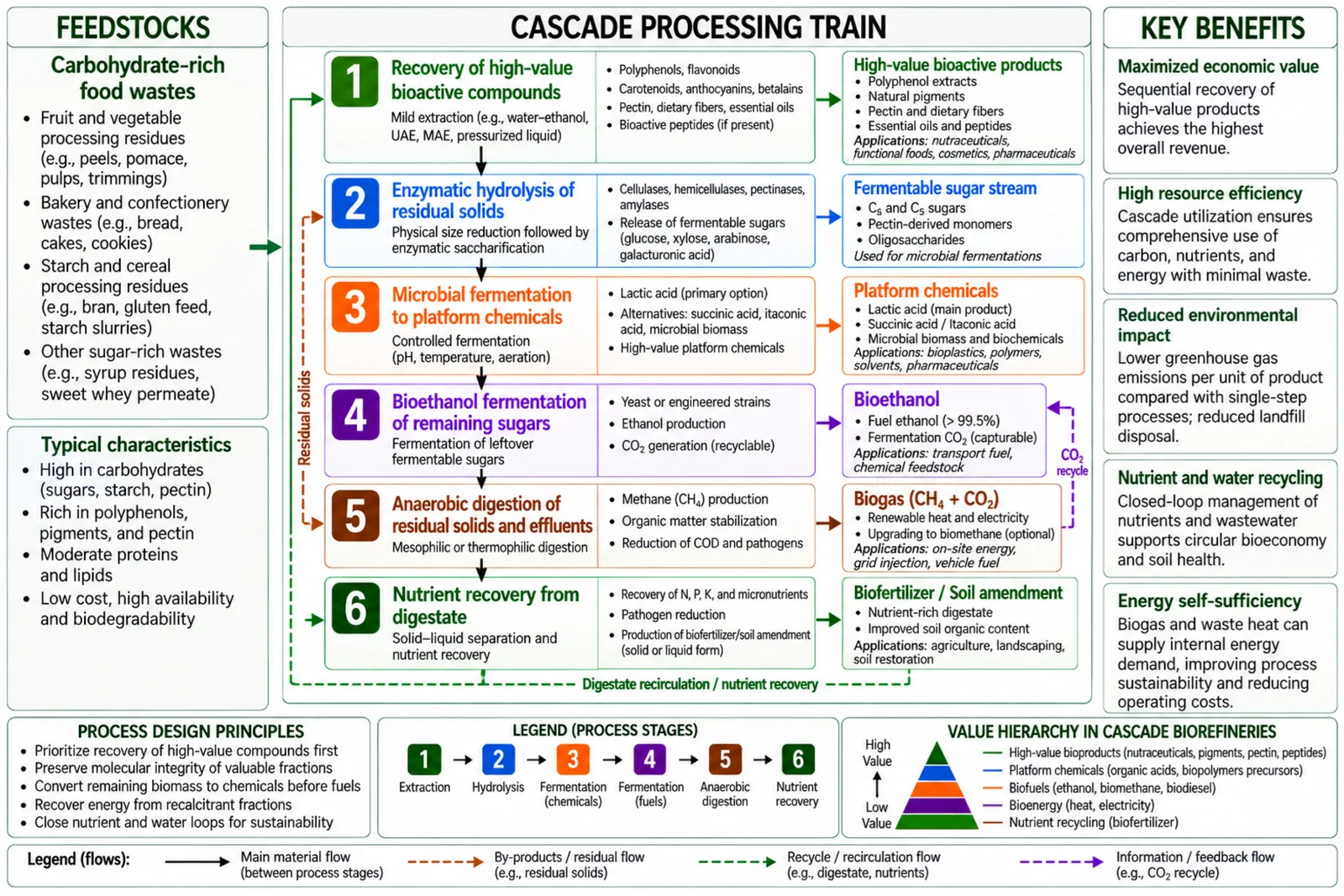
Conceptual cascade biorefinery for carbohydrate-rich food waste emphasizing sequential value recovery. High-value compounds are recovered in the initial stages, followed by the production of platform chemicals and bioethanol, while residual solids are subsequently converted into biogas and biofertilizers, thereby enabling sequential utilization of the available biomass fractions. The proposed cascade illustrates how integrated processing can improve resource efficiency, carbon allocation, and overall biorefinery sustainability.

The proposed configuration (Figure 6) follows the principles of cascade biorefining by prioritizing the recovery of high-value compounds before biochemical conversion of the remaining carbohydrate fraction. Mild pretreatment and fractionation preserve heat-sensitive molecules such as polyphenols, pigments and pectin, while the carbohydrate-rich hydrolysate is subsequently directed toward the production of platform chemicals or bioethanol, depending on feedstock composition and market demand [30,203]. Residual solids are finally converted by anaerobic digestion, enabling energy recovery and nutrient recycling through digestate utilization [25]. Compared with conventional single-product processing, this sequential allocation improves carbon utilization and diversifies product portfolios while reducing the quantity of organic material directed immediately to bioenergy production.

Recent literature consistently supports this cascade strategy but also highlights several technical challenges. The recovery of bioactive compounds often requires mild extraction conditions that preserve molecular functionality, whereas efficient fermentation generally benefits from more intensive pretreatment to increase sugar availability. Optimizing these potentially conflicting requirements remains one of the principal design challenges for carbohydrate-based biorefineries [88,200]. In addition, seasonal variability in fruit and vegetable residues affects sugar composition, moisture content and concentrations of phenolic compounds, making flexible process control and feedstock characterization essential for maintaining stable product quality.

From an industrial perspective, carbohydrate-rich food waste represents one of the most promising feedstocks because several downstream technologies, including ethanol fermentation, lactic acid production and anaerobic digestion, have already reached advanced technology readiness levels. By contrast, the selective recovery of pectin, polyphenols and natural pigments often remains economically viable only when sufficient product quality and market demand justify the additional processing steps [204,205]. The optimal configuration depends not on maximizing the recovery of every biomass component but on balancing product value, technology readiness, downstream processing costs and overall process integration [78,205]. This illustrates a recurring principle throughout integrated food-waste biorefineries: the most efficient configuration is not necessarily the one recovering the greatest number of products, but the one achieving the best balance between resource efficiency, economic performance and industrial feasibility [204,205].

### 7.2. Protein-Rich Food Waste Biorefinery

Protein-rich food wastes, including meat and fish by-products, dairy residues, brewery spent yeast, and protein-rich processing streams, require a distinct biorefinery strategy because their principal value lies in the recovery and preservation of nitrogen-containing molecules, with carbon conversion serving as a complementary valorization pathway. Unlike carbohydrate-rich feedstocks, where fermentable sugars are the primary target, protein-rich residues offer opportunities for producing protein isolates, bioactive peptides, enzymes and single-cell proteins (SCPs), while the remaining organic matter can subsequently be converted into bioenergy and biofertilizers [206,207].

**Figure 7:**
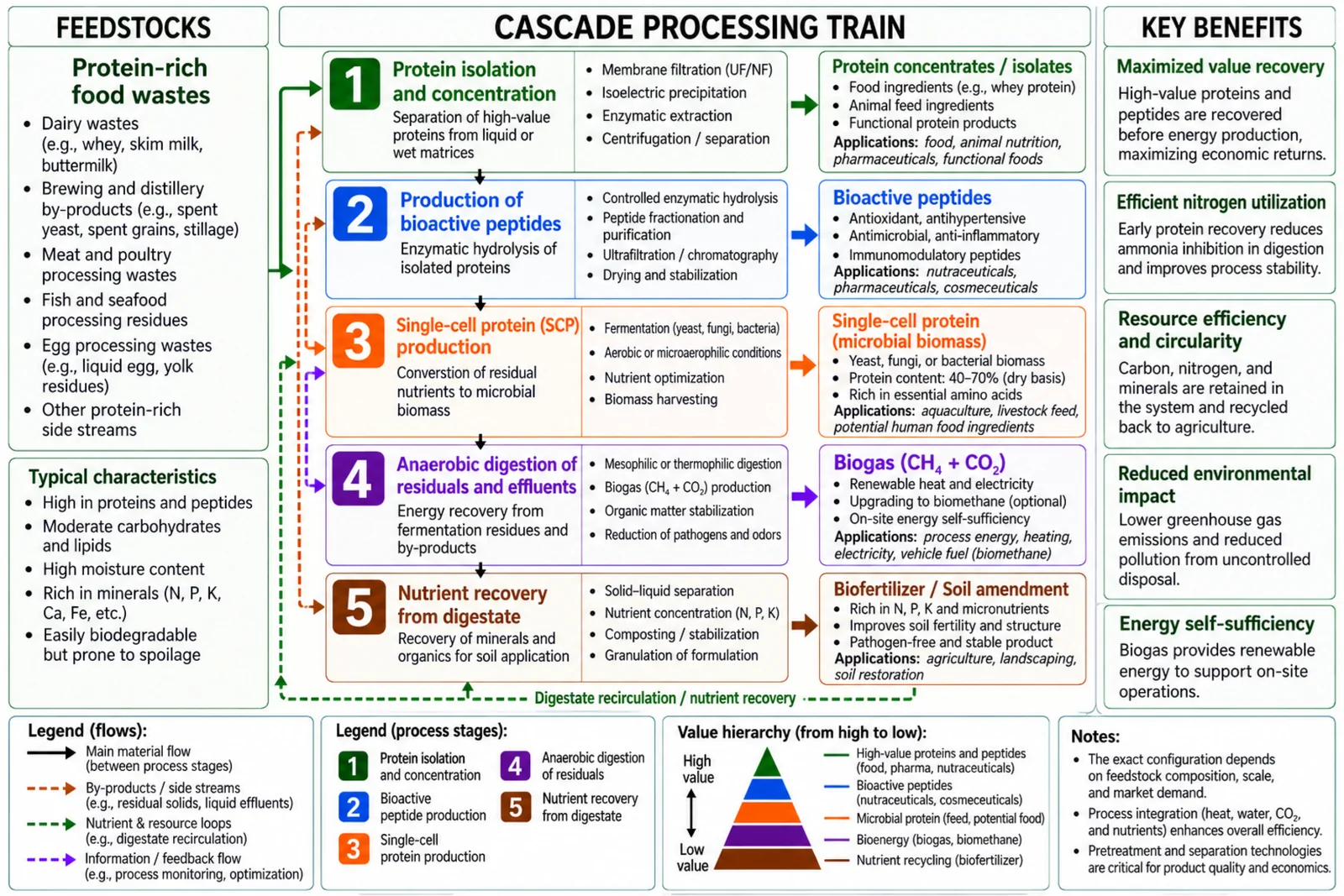
Conceptual cascade biorefinery for protein-rich food waste emphasizing sequential nutrient recovery. Protein-rich food wastes (e.g., dairy by-products, brewery residues, meat and fish processing wastes, and egg-processing side streams) are sequentially valorized through protein isolation, production of bioactive peptides, microbial conversion into single-cell proteins, anaerobic digestion of residual biomass, and nutrient recovery from digestate. By prioritizing recovery of high-value protein fractions before energy generation, the proposed cascade enhances nitrogen utilization, reduces ammonia inhibition during anaerobic digestion, improves overall carbon and nutrient efficiency, and supports circular bioeconomy principles.

Previous studies recommend prioritizing protein recovery before biological degradation to improve both resource efficiency and product value [206,208]. The proposed configuration (Figure 7) follows a sequential strategy in which proteins are first recovered or hydrolysed into peptides and amino acids for food, feed or biotechnological applications [206,207]. Depending on feedstock characteristics, the remaining liquid fraction may support microbial cultivation for enzyme or SCP production, whereas residual solids are directed to anaerobic digestion for energy recovery and nutrient recycling. This integrated approach preserves the functional value of proteins while reducing nitrogen losses that would otherwise occur during direct biological conversion. Compared with conventional anaerobic digestion, the proposed configuration improves nitrogen utilization and generates products with substantially higher added value.

Recent research highlights several challenges associated with protein-rich feedstocks. Their high nitrogen content favours ammonia formation during anaerobic digestion, which may inhibit methanogenic microorganisms and reduce process stability [51]. Early protein extraction can alleviate this limitation while simultaneously creating additional revenue streams. However, extraction efficiency must be balanced against energy consumption, hydrolysis conditions and product purity, particularly when proteins are intended for food or feed applications [206,209]. Feedstock heterogeneity, rapid microbial spoilage and the presence of fats or mineral salts further complicate process design and require rapid stabilization after collection.

Industrial implementation is influenced not only by technological feasibility but also by regulatory requirements governing the use of recovered proteins in food and feed products. Food-processing by-products generally offer greater commercialization potential than mixed post-consumer food waste because they provide higher compositional consistency, lower contamination risks and improved traceability. Studies addressing similar issues emphasize the importance of combining selective protein recovery with robust downstream purification and quality assurance systems to ensure compliance with stringent safety standards [206,210]. These considerations demonstrate that successful protein-based biorefineries depend as much on regulatory and market factors as on advances in extraction or fermentation technologies.

Protein-rich biorefineries illustrate a fundamental principle of integrated food-waste valorization: preserving the functional properties of biomass before biological degradation generally provides greater economic and environmental benefits than direct energy recovery. The optimal configuration therefore seeks to maximize nitrogen utilization through sequential recovery of proteins and peptides, microbial conversion of intermediate streams and final recovery of energy and nutrients from residual biomass, fully consistent with the principles of cascade and circular biorefineries.

### 7.3. Lipid-Rich Food Waste Biorefinery

Lipid-rich food wastes, including waste cooking oil, grease-trap residues, dairy fats and oil-rich food-processing by-products, represent attractive feedstocks because of their high energy density and broad potential for producing fuels, oleochemicals and other lipid-derived products. Unlike carbohydrate- or protein-rich residues, these substrates require process configurations that preserve lipid functionality before biological degradation, allowing oils and fatty acids to be directed toward higher-value applications while utilizing residual fractions for energy and nutrient recovery. Similar studies emphasize selective lipid recovery as the initial step in integrated lipid-based biorefineries, enabling the subsequent valorization of the remaining feedstock through anaerobic digestion [141,211].

**Figure 8:**
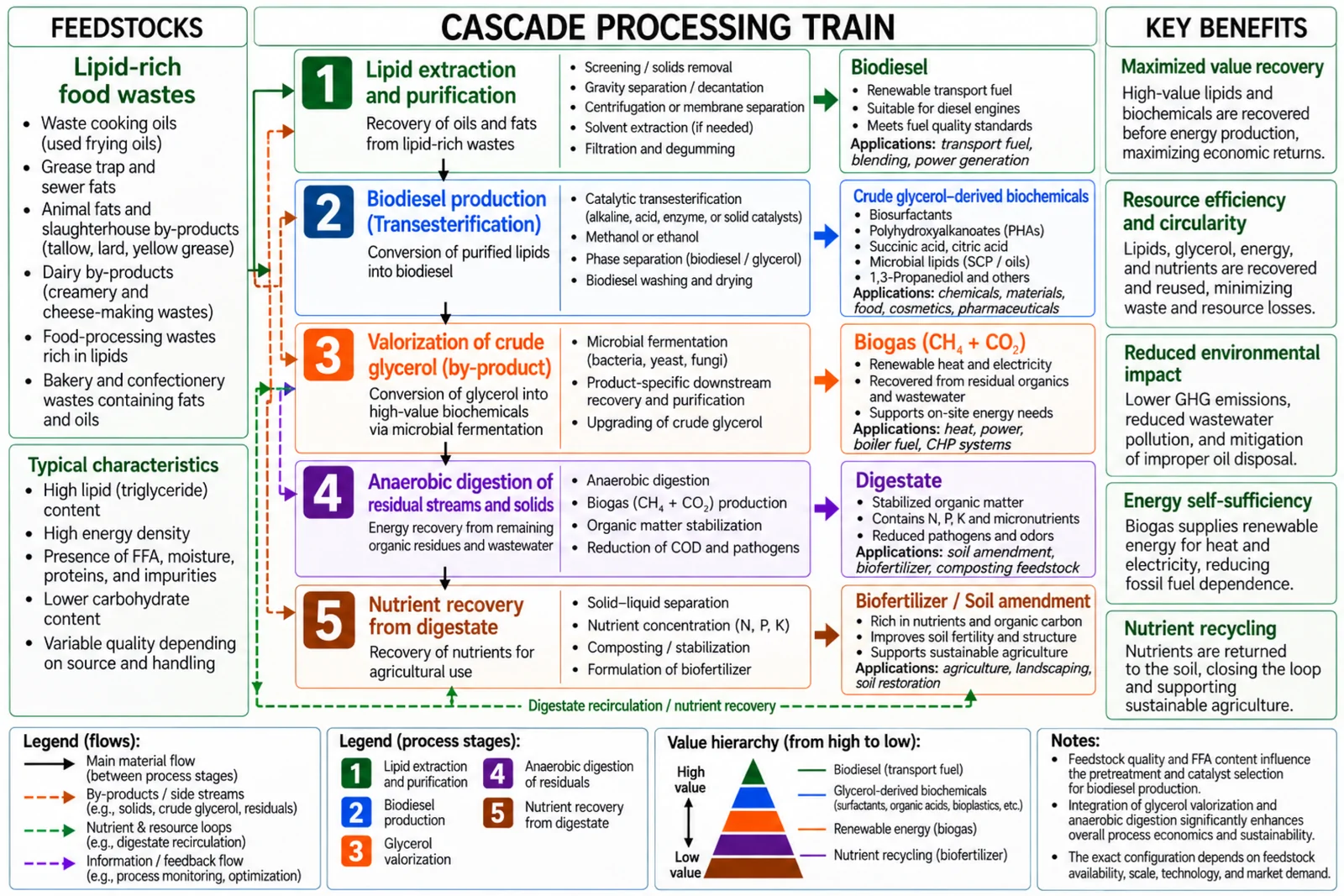
Conceptual cascade biorefinery for lipid-rich food waste emphasizing integrated lipid valorization. Lipid-rich food wastes (e.g., waste cooking oils, grease-trap residues, dairy fats, and other fat-rich food-processing by-products) are sequentially valorized through lipid extraction, biodiesel production, microbial conversion of crude glycerol into value-added biochemicals, anaerobic digestion of residual streams, and nutrient recovery from digestate. By integrating biodiesel production with glycerol valorization and renewable energy generation, the proposed cascade maximizes carbon utilization, improves process economics, minimizes waste generation, and supports circular bioeconomy principles.

The proposed configuration (Figure 8) prioritizes the separation and purification of lipid fractions before their conversion into biodiesel, oleochemicals or other bio-based products. Depending on feedstock quality, recovered lipids may be processed through transesterification to produce biodiesel or redirected toward the synthesis of biosurfactants, microbial lipids or specialty chemicals. Glycerol generated during biodiesel production should not be regarded as a low-value by-product but as an additional carbon source that can support the production of platform chemicals or microbial biomass [211]. Residual organic matter is subsequently converted through anaerobic digestion, while digestate is recycled as a source of nutrients for agricultural applications, improving overall carbon and nutrient recovery.

The literature consistently indicates that waste cooking oil represents the most mature lipid feedstock because collection systems, transesterification technologies and biodiesel markets are already well established. In contrast, mixed lipid-rich food wastes present greater technical challenges owing to variable moisture content, free fatty acid concentrations and contamination with suspended solids or cleaning agents. These characteristics influence pretreatment requirements, catalyst selection and biodiesel quality, often making feedstock conditioning a critical determinant of process performance [141]. Furthermore, long-chain fatty acids released during hydrolysis may inhibit anaerobic digestion if excessive lipid residues are directed to biological treatment, highlighting the importance of balancing lipid extraction with subsequent energy recovery [70]. Recent research has expanded the role of lipid-rich biorefineries beyond biodiesel production by exploring microbial oils, biosurfactants and other oleochemical products with higher market value [96,212,213]. However, these emerging pathways generally remain at lower technology readiness levels than conventional biodiesel because they require more sophisticated fermentation systems and costly downstream purification [96,212]. Their successful implementation therefore depends on integrating mature lipid-processing technologies with emerging microbial conversion routes, enabling progressive diversification of product portfolios without compromising industrial reliability [212,213].

Lipid-rich biorefineries demonstrate that high resource efficiency can be achieved through the sequential utilization of lipid fractions according to their functional value, ensuring that high-value applications are prioritized before their eventual use for energy recovery. Prioritizing the recovery of oils for fuels and specialty products, followed by the biological conversion of residual biomass into biogas and biofertilizers, provides a balanced strategy that combines technological maturity with opportunities for future innovation. This configuration illustrates how established industrial processes can be integrated with emerging biotechnologies to improve both economic performance and circular resource utilization.

### 7.4. Integrated Biorefinery for Mixed Municipal Food Waste

**Figure 9:**
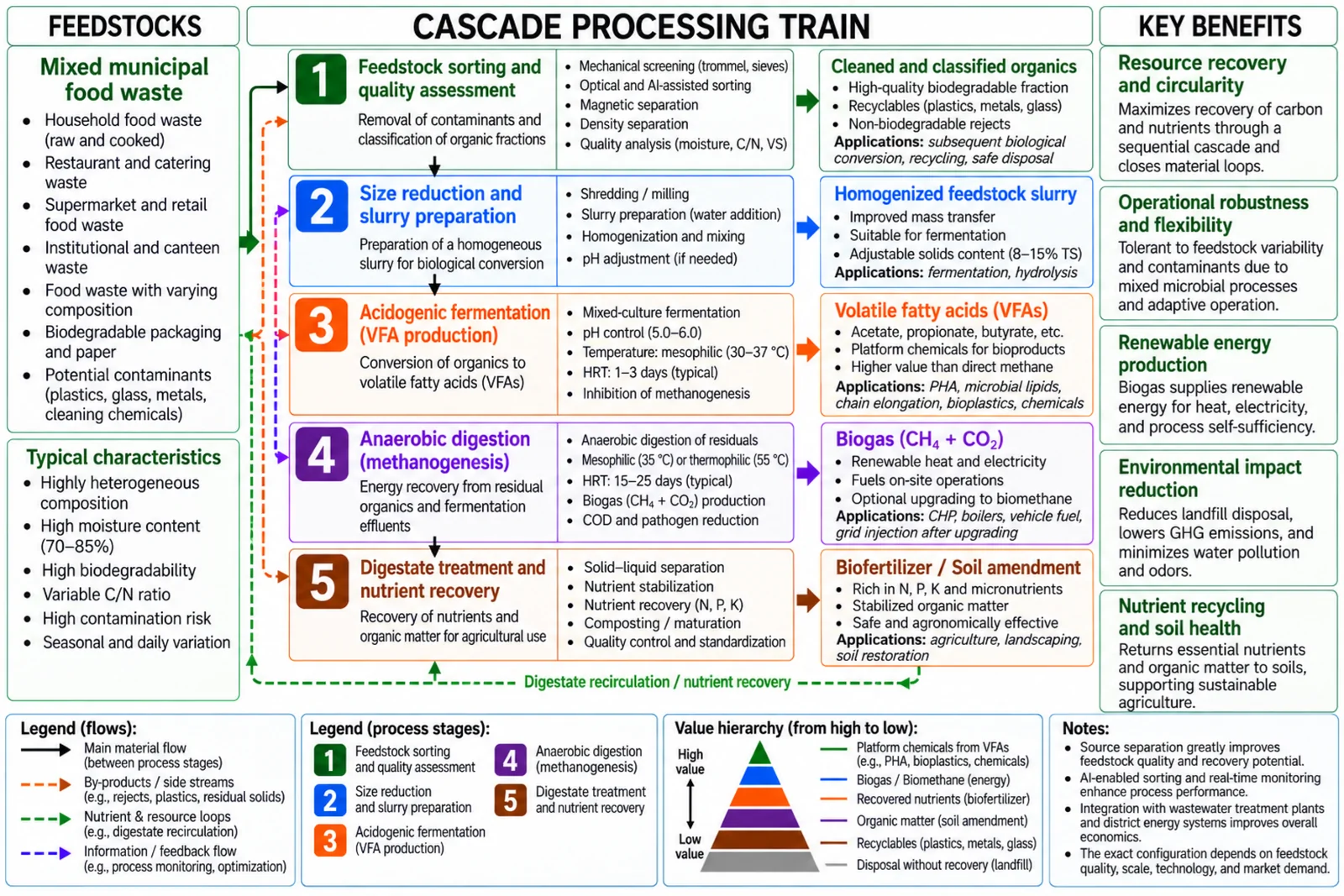
Conceptual cascade biorefinery for mixed municipal food waste emphasizing robust resource recovery and process flexibility. Mixed municipal food waste is sequentially processed through feedstock sorting and quality assessment, slurry preparation, acidogenic fermentation for volatile fatty acid (VFA) production, anaerobic digestion of residual biomass, and nutrient recovery from digestate. The proposed cascade prioritizes process robustness over selective extraction, enabling the production of platform chemicals, renewable biogas, and biofertilizers while maximizing carbon and nutrient recovery, accommodating feedstock heterogeneity, and supporting circular bioeconomy principles.

Mixed municipal food waste represents the most abundant but also the most challenging feedstock for integrated biorefineries because it combines highly variable proportions of carbohydrates, proteins, lipids, lignocellulosic materials and inorganic contaminants. Unlike relatively homogeneous industrial by-products, its composition changes continuously according to dietary habits, seasonality, collection systems and geographical location, making process optimization considerably more complex [78,214]. Recent research has shifted from designing fixed process configurations toward developing flexible biorefineries capable of adapting to feedstock variability while maintaining stable product quality and process performance [78]. The proposed configuration (Figure 9) adopts a systems-based approach in which mechanical sorting, pretreatment, and fractionation constitute the key enabling operations. Individual biomass fractions are subsequently allocated to appropriate conversion pathways according to their composition and valorization potential, enabling more effective utilization of the heterogeneous feedstock [78,212]. Carbohydrate-rich streams are preferentially converted into platform chemicals or bioethanol, lipid fractions are recovered for biodiesel or oleochemicals, protein-rich materials support the production of protein-based products or microbial biomass, while the remaining organic residues undergo anaerobic digestion for biogas production and nutrient recovery. This sequential allocation maximizes carbon utilization and reduces the irreversible loss of valuable biomass components through direct energy recovery [212].

Existing research consistently identifies feedstock heterogeneity as the principal limitation of municipal food-waste biorefineries. Variations in moisture content, particle size, contamination level and organic composition affect pretreatment efficiency, fermentation performance and downstream separation, often leading to fluctuations in product yield and quality [78,215]. For this reason, modern process design incorporates online monitoring, rapid feedstock characterization, and flexible operating conditions to accommodate continuous changes in substrate composition. These approaches improve process resilience and reduce the dependence on highly standardized feedstocks, which are rarely available in municipal waste management systems [78].

Another distinguishing feature of mixed municipal food waste is the need to integrate technological, logistical and regulatory considerations. Collection strategies, source separation efficiency, contaminant removal and local waste management infrastructure frequently determine the feasibility of resource recovery as much as the conversion technologies themselves [216]. Successful implementation depends on coordinating feedstock logistics with biorefinery operation, while simultaneously ensuring compliance with environmental and food safety regulations. These system-level interactions explain why technologies demonstrating excellent laboratory performance often encounter significant barriers during industrial deployment [215,216].

Recent advances in digital technologies offer new opportunities to address these challenges. Artificial intelligence, machine learning, digital twins, and advanced sensor systems are being investigated to support real-time feedstock characterization, predictive process control, and dynamic allocation of biomass fractions among competing conversion pathways. These tools facilitate the transition from independent optimization of individual unit operations toward adaptive management of the entire biorefinery, improving resource efficiency while responding to fluctuations in feedstock composition, market demand, and operational conditions [183]. Such developments are expected to play an important role in future integrated food-waste biorefineries, particularly those processing heterogeneous municipal waste streams.

Mixed municipal food waste illustrates the transition from process optimization to systems optimization. The objective is no longer to maximize the performance of a single conversion technology but to coordinate multiple processing routes within an integrated and adaptive biorefinery capable of responding to continuous variations in feedstock characteristics [78]. This systems-oriented strategy represents the most realistic pathway for implementing circular food-waste biorefineries at industrial scale, where operational flexibility is often more valuable than maximizing the performance of any individual process.

### 7.5. Design Principles for Integrated Food-Waste Biorefineries

The representative biorefinery scenarios presented in Section 6.1, Section 6.2, Section 6.3 and Section 6.4 demonstrate that integrated food-waste biorefineries cannot be designed using a universal process configuration. Instead, successful process design requires the simultaneous consideration of feedstock composition, product hierarchy, technology selection, resource recovery and system integration [70,78]. Although individual process layouts differ according to the dominant biomass fraction, several common engineering principles consistently emerge from recent research and provide the foundation for developing efficient and resilient food-waste biorefineries [70]. These principles extend beyond the optimization of individual unit operations and reflect a systems-oriented approach that integrates pretreatment, fractionation, biochemical conversion, bioenergy production, and resource recovery into a coordinated process network. Overall performance is determined by both the efficiency of individual operations and the interactions among material, energy, and information flows throughout the biorefinery. The principal design principles identified from the literature are summarized in Table 13.

The first principle is that process configuration should be guided by feedstock characteristics. Differences in carbohydrate, protein, and lipid contents, moisture, contaminants, and seasonal variability require tailored pretreatment strategies, separation methods, and downstream conversion pathways [6,78]. Selecting technologies without adequate feedstock characterization frequently results in inefficient resource utilization and unnecessary process complexity.

A second principle concerns cascade utilization of biomass, whereby food waste is sequentially converted into products according to their functional and economic value. High-value compounds such as polyphenols, pigments, proteins or lipids should be recovered before the remaining biomass is directed toward platform chemicals, biofuels and, ultimately, energy recovery [70,88]. This hierarchy preserves molecular functionality, increases carbon utilization and generally improves the economic potential of integrated biorefineries.

Another recurring principle is the integration of material and energy flows. Water recycling, digestate utilization, carbon dioxide capture, waste heat recovery and internal reuse of process streams reduce external resource consumption while improving environmental performance. Integrated multi-technology systems can enhance multilevel resource utilization and energy recovery, thereby potentially improving system-level sustainability depending on process configuration and system boundaries [70].

Equally important is the integration of engineering, economic and environmental criteria during process development. The previous chapters have shown that technology selection cannot rely exclusively on laboratory performance or product yield. Process configurations should therefore be evaluated using complementary indicators such as technology readiness, TEA, LCA and, where appropriate, multi-criteria decision analysis (MCDA). Such integrated evaluation enables more robust decisions by balancing technical feasibility, economic viability and environmental performance throughout the entire biorefinery [70,181].

Finally, future food-waste biorefineries may increasingly benefit from adaptive process-management approaches capable of responding to variations in feedstock composition, market demand, and operating conditions. Digitalization, online monitoring, artificial intelligence (AI), and digital-twin concepts offer potential tools for improving process-state estimation, predictive control, biomass allocation, and resource recovery. However, the maturity of these approaches remains heterogeneous. Online sensing and process monitoring are already established in several bioprocessing contexts, whereas AI-assisted optimization, digital twins, and reinforcement-learning-based control for waste-biorefineries are represented more frequently by simulation studies, modelling frameworks, laboratory-scale investigations, or proof-of-concept applications than by sustained pilot- or industrial-scale deployment [182,183]. Accordingly, their capacity to support dynamic optimization of integrated food-waste biorefineries should currently be regarded as an emerging opportunity rather than a generally demonstrated industrial capability.

These design principles demonstrate that future food-waste biorefineries will evolve from collections of individual conversion technologies into integrated production systems in which feedstock characterization, cascade valorization, process integration and adaptive control are considered simultaneously. The successful implementation of such systems will depend not only on continued advances in conversion technologies but also on their effective integration into flexible and economically viable biorefinery configurations [70,181].

## 8. Sustainability Assessment of Food-Waste Biorefineries

The sustainability of food-waste biorefineries cannot be inferred from conversion efficiency or resource recovery alone. System-level assessment should integrate environmental, economic, technological, and circularity dimensions through complementary approaches including LCA, TEA, technology-readiness evaluation, and circularity indicators [5,70,87]. Together, these assessment tools provide complementary perspectives for balancing technical feasibility, environmental performance and economic viability throughout biorefinery development.

A comprehensive sustainability assessment should therefore integrate environmental, economic and technological criteria to identify biorefinery configurations that improve resource efficiency while remaining commercially viable and environmentally responsible [70,87]. Such an integrated approach provides the basis for comparing alternative valorization pathways and supporting the development of resilient food-waste biorefineries aligned with the principles of the circular bioeconomy.

### 8.1. Why Sustainability Metrics Matter

The diversity of food-waste feedstocks and biorefinery configurations makes direct comparison of valorization pathways inherently difficult. Carbohydrate-, protein- and lipid-rich feedstocks differ substantially in composition, conversion efficiency, product portfolio and market value, while pretreatment, downstream processing and process integration further influence overall performance. Technology selection cannot rely on product yield or conversion efficiency alone [5,70].

Studies addressing similar issues demonstrate that maximizing the recovery of a single product does not necessarily lead to the most sustainable biorefinery configuration. More intensive extraction or purification may increase product recovery while simultaneously increasing energy demand, water consumption, chemical use and operating costs. Conversely, cascade and multiproduct biorefineries can improve overall resource utilization by recovering several valuable fractions and directing residual streams toward subsequent conversion or energy recovery [70,87,217]. These trade-offs highlight the need for system-level evaluation instead of optimization based solely on individual process steps.

The assessment of integrated food-waste biorefineries therefore requires complementary sustainability metrics that capture environmental performance, economic feasibility, resource circularity and technological feasibility [5,70]. TEA, circularity indicators and technology-readiness considerations provide complementary information for comparing alternative configurations and assessing their potential for industrial implementation. Their combined use can support more balanced decision-making by integrating technical performance with environmental and economic criteria throughout biorefinery development [50,70,87].

### 8.2. Environmental Sustainability Indicators

Environmental sustainability is one of the principal motivations for developing food-waste biorefineries, yet environmental benefits cannot be inferred solely from the use of renewable feedstocks. Energy-intensive pretreatment, downstream purification, chemical consumption, and transportation may offset part of the environmental benefits associated with food-waste valorization [96,168]. Environmental performance therefore requires a system-level assessment that accounts for the combined effects of these factors across the entire biorefinery. LCA provides a standardized framework for comparing alternative food-waste valorization pathways because it considers environmental burdens across defined stages of the production chain, including feedstock collection, pretreatment, conversion, product recovery and residual-stream management. Related research demonstrate that food-waste valorization can provide substantial environmental benefits compared with conventional products or waste-management alternatives; however, the magnitude and even distribution of these benefits depend strongly on methodological choices and background conditions [96,221]. System boundaries, functional units, allocation procedures, electricity supply and assumptions concerning avoided products can substantially influence the results and should therefore be transparently defined when comparing alternative pathways.

Comparison of the representative LCAs summarized in Table 14 demonstrates that methodological choices can materially affect the apparent environmental performance of food-derived residual-stream valorization pathways. A further distinction is required between attributional and consequential LCA. Attributional LCA characterizes the environmental burdens associated with a defined product system under specified allocation rules and is therefore appropriate for describing the environmental profile of a particular valorization configuration. Consequential LCA, by contrast, evaluates the environmental consequences of changes in production or waste-management decisions and is particularly relevant when food-derived residual streams are diverted from existing uses or when recovered products are assumed to displace conventional products. In such cases, assumptions concerning marginal substitution, avoided production, and displaced food, feed, fertilizer, energy, or industrial uses can substantially influence the resulting environmental balance.

In particular, conclusions depend strongly on whether the assessment adopts a waste-management or product-production perspective, on the functional unit selected, and on whether credits are assigned for avoided conventional products, energy generation, or displaced waste-treatment operations. System expansion or substitution can substantially improve the apparent performance of multiproduct biorefineries when recovered materials or energy replace conventional products, whereas narrower process-level boundaries may emphasize the burdens associated with pretreatment, electricity consumption, chemical inputs, and downstream purification. The assumed electricity mix is particularly influential for energy-intensive processing, while allocation becomes important where several products or co-products are generated. Consequently, apparently conflicting conclusions among LCAs do not necessarily indicate contradictory process performance but may instead reflect differences in system boundaries, functional units, allocation procedures, background energy systems, and reference scenarios. These methodological differences should therefore be considered explicitly when using LCA to compare food-waste biorefinery configurations.

Among the environmental indicators reported in food-waste valorization studies, greenhouse-gas emissions, energy demand and water use are particularly relevant for identifying environmental trade-offs. Process intensification and increased product recovery may improve resource utilization but can also increase electricity, heat, chemical and water requirements, particularly during pretreatment and downstream separation [96,168]. Process integration, energy recovery and internal resource reuse can reduce external inputs and improve the environmental performance of integrated systems.

Beyond climate change, comprehensive LCAs consider impact categories such as eutrophication, acidification, human toxicity, ecotoxicity, and resource depletion. These indicators are particularly relevant when chemical pretreatment, solvent extraction or intensive purification steps are involved, because improvements in one environmental category may be accompanied by increased burdens in others [89]. Maximizing product recovery alone cannot be assumed to minimize environmental impacts.

Overall, the recent literature indicates that the environmental sustainability of food-waste biorefineries is strongly influenced by process integration, energy and chemical requirements, feedstock characteristics and methodological choices in the assessment [70,168]. Future assessments should therefore prioritize transparent and harmonized LCA methodologies together with system-level comparisons capable of capturing interactions among pretreatment, conversion, downstream processing and resource recovery.

For food-processing side streams with established uses, the choice of reference scenario is particularly important. An LCA that treats whey, brewer’s spent grain, oilseed cake, or similar streams as burden-free wastes may produce different conclusions from an assessment that recognizes their existing feed, food, fertilizer, or industrial functions. Where diversion from an established use may induce changes elsewhere in the system, a consequential LCA perspective is particularly relevant because it can account for marginal substitution and the environmental consequences of displaced food, feed, fertilizer, energy, or industrial functions. Where a full consequential analysis is not performed, these potential displacement effects should at minimum be identified explicitly and examined through transparent reference-scenario and sensitivity assumptions. Consequently, environmental preference among alternative valorization pathways cannot be inferred solely from conversion yield or product value.

### 8.3. Techno-Economic Assessment

Economic viability remains a major barrier to the large-scale implementation of food-waste biorefineries. Techno-economic assessment (TEA) evaluates whether the technical performance of a proposed configuration can translate into commercial feasibility when capital and operating costs, product prices, feedstock characteristics, processing capacity, and downstream operations are considered [78,87].

Recent TEA studies demonstrate that economic performance is determined by the interaction among product portfolio, process configuration, production scale and market conditions, and cannot be inferred from the yield of a single product alone. Integrated biorefineries can improve economic performance by generating multiple products and utilizing residual streams; however, additional processing steps may also increase capital expenditure, operational complexity and separation costs [78,87]. System-level TEA can determine whether the additional revenues generated through diversification are sufficient to justify the associated processing costs.

Feedstock characteristics further influence economic performance through their effects on pretreatment requirements, conversion efficiency and logistics. The composition and availability of municipal food waste can substantially alter the economic feasibility of a biorefinery, particularly when product yields depend strongly on specific feedstock fractions [78]. Transportation distance, processing capacity and feedstock availability can also influence production economics, emphasizing the need to integrate supply-chain considerations into biorefinery design. For industrial side streams, economic assessment should additionally account for the opportunity cost associated with existing uses. A feedstock that already has a market as an ingredient, animal feed, fertilizer, or industrial raw material cannot necessarily be treated as a zero-cost input, and diversion to a new biorefinery pathway may create additional costs or market effects associated with replacing the displaced function. These factors should therefore be incorporated into feedstock-oriented TEA wherever relevant.

Sensitivity analyses reported in food-waste valorization studies identify product selling price, processing capacity, feedstock characteristics or costs, capital and operating expenditures, and energy- or process-specific inputs among the parameters that can strongly influence profitability [87,227]. Sensitivity analysis should be complemented, particularly for emerging technologies, by explicit uncertainty analysis because scale-up factors, conversion yields, product prices, equipment and capital costs, and other techno-economic assumptions may remain poorly constrained at early stages of development. Whereas sensitivity analysis identifies the parameters to which economic outcomes are most responsive, uncertainty analysis evaluates how uncertainty in these inputs propagates to indicators such as minimum selling price, net present value, internal rate of return, or payback period. Probabilistic approaches, parameter ranges, or scenario-based uncertainty analysis can therefore provide a more realistic representation of economic risk and reduce the risk of interpreting point estimates as robust predictions of industrial performance. TEA can also be combined with LCA to identify configurations that balance economic performance with environmental impacts [87]. To provide a quantitative perspective on economic feasibility, representative techno-economic assessments of food-derived residual-stream valorization systems are compared in Table 15. Because the reported economic indicators depend on plant capacity, process configuration, product portfolio, economic assumptions, and geographical context, the values are presented together with their principal system assumptions rather than treated as directly transferable benchmarks.

The quantitative evidence summarized in Table 15 confirms that economic feasibility is strongly scenario-dependent. Across the representative studies, profitability is influenced not only by conversion performance but also by processing capacity, product portfolio, feedstock-related costs or gate fees, downstream-processing requirements, capital intensity, operating expenditure, and product or energy selling prices. Consequently, indicators such as minimum selling price, NPV, IRR, ROI, production cost, and payback period should not be compared independently of the assumptions under which they were calculated. Sensitivity analyses further show that the dominant economic drivers vary among process configurations: product selling price and plant throughput can determine the viability of integrated product-oriented biorefineries, whereas gate fees, transportation requirements, energy prices, and infrastructure availability can become decisive for waste-management-oriented configurations. The reported values should therefore be interpreted as scenario-specific evidence of economic performance rather than universal benchmarks for a particular conversion technology or product. Where multiple technical, economic and environmental criteria must be considered simultaneously, multi-criteria decision analysis (MCDA) can provide an additional decision-support framework.

Overall, current evidence indicates that the economic feasibility of food-waste biorefineries depends on the coordinated optimization of feedstock utilization, process configuration, production scale, product portfolio and downstream operations. Techno-economic assessment should therefore be incorporated during process development to identify major cost drivers, evaluate scale-up scenarios and support the transition from laboratory research toward industrial implementation [78,87].

### 8.4. Circularity Assessment

Circularity has become a central objective of food-waste biorefineries, reflecting the transition from waste management toward regenerative resource utilization. However, circularity should not be inferred simply from the use of food waste as a feedstock. The extent to which a biorefinery contributes to a circular bioeconomy depends on its ability to retain and recover material and energy resources while minimizing external inputs and residual waste generation [5,70].

Recent research recognizes circularity as a multidimensional systems property involving material recovery, energy utilization, nutrient recycling, resource productivity, and the reduction in virgin-resource inputs. A range of quantitative indicators has been proposed in the broader circular-economy literature, including the Material Circularity Indicator (MCI), material-flow-based circularity indices, resource-recovery efficiencies, nutrient-recovery indicators, and measures of energy or resource self-sufficiency. However, these indicators should not be interpreted as being routinely applied to food-waste biorefineries. Within the literature reviewed here, direct empirical application of standardized circularity metrics such as the MCI to complete food-waste biorefinery systems remains limited. Most studies instead characterize circular performance indirectly through system-specific measures such as waste diversion, product and by-product recovery, carbon or nutrient recovery, energy recovery, internal reuse of process water and residual streams, or reductions in external resource requirements [5,70]. Consequently, the MCI and related standardized circularity indices are considered here primarily as methodological tools available for future system-level assessment rather than as metrics that have already been consistently applied across the reviewed food-waste biorefinery literature.

Integrated biorefineries can enhance circular resource utilization by combining cascade valorization with the recovery and reuse of residual streams. Process-water recycling, digestate utilization, nutrient recovery, energy recovery and internal reuse of process streams can reduce external resource requirements and increase utilization of the original feedstock. However, increasing resource recovery may require additional separation, purification and recycling operations, potentially increasing energy demand, capital costs and process complexity. Resource recovery should therefore be evaluated together with its environmental and economic trade-offs [5,70]. These system-specific indicators provide useful evidence of resource recovery but are not equivalent to a harmonized circularity assessment. Differences in indicator definitions, system boundaries, recovered-resource accounting, and treatment of internally recycled streams currently prevent robust quantitative comparison of circularity among many published food-waste biorefinery configurations.

Recent research also emphasizes the complementary use of circularity indicators and life-cycle-based indicators because a high degree of material circularity does not necessarily correspond to low environmental impacts [6]. Combining circularity assessment with LCA, and where appropriate TEA, can therefore provide a more comprehensive evaluation of biorefinery performance. This integrated perspective recognizes that highly circular configurations are not automatically those with the lowest environmental burdens or greatest economic returns; process configurations should instead be evaluated across resource, environmental and economic dimensions.

Therefore, circularity should be assessed as a measurable systems property using clearly defined and quantifiable indicators. The present review also identifies an important evidence gap: although circularity is frequently invoked as a central objective of food-waste valorization and integrated biorefineries, standardized circularity metrics have not yet been applied consistently across these systems. Empirical studies more commonly report individual resource-recovery or waste-reduction indicators than comprehensive circularity indices, which limits cross-study comparison and prevents the establishment of robust circularity benchmarks. Future research should therefore move beyond qualitative claims of circularity and explicitly report material, carbon, nutrient, water, and energy flows using clearly defined system boundaries and harmonized indicators. Where appropriate, standardized circularity metrics should be evaluated alongside LCA and TEA so that improvements in material recirculation can be assessed together with their environmental and economic consequences.

### 8.5. Integrated Sustainability Assessment Framework

The evaluation of food-waste biorefineries requires a holistic framework that integrates environmental, economic, technological and circularity perspectives. Individual indicators provide valuable but incomplete information, as technologies exhibiting favorable environmental performance may be economically uncompetitive, while economically attractive processes may offer limited resource efficiency or remain insufficiently mature for industrial deployment [181,210]. Sustainable process design requires the simultaneous consideration of multiple and potentially competing performance criteria.

**Figure 10:**
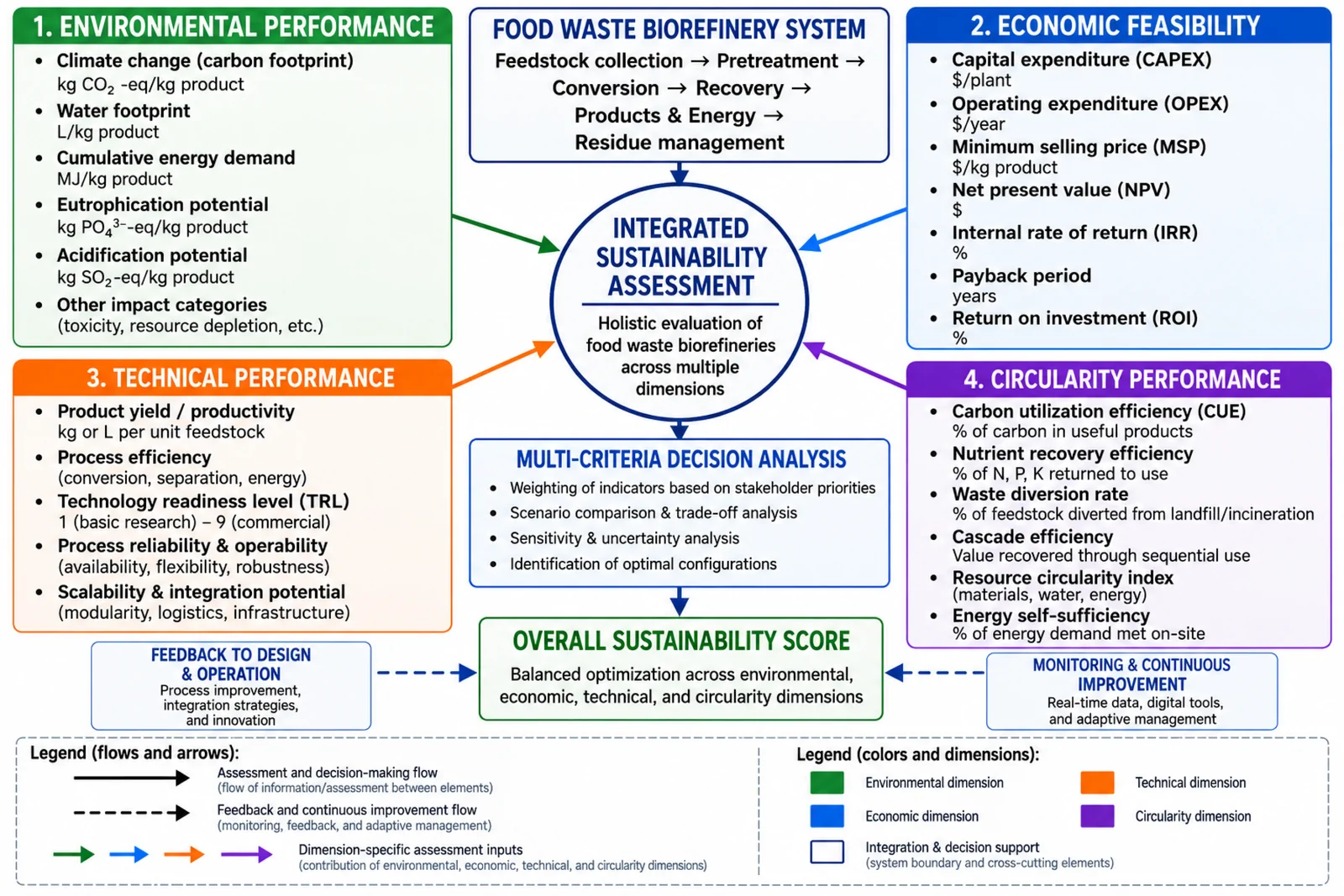
Proposed conceptual framework for integrated sustainability assessment of food-waste biorefineries. The framework organizes four complementary assessment dimensions: environmental performance (e.g., carbon footprint, water footprint, cumulative energy demand, and other life-cycle impacts), economic feasibility (e.g., CAPEX, OPEX, minimum selling price, net present value, internal rate of return, and return on investment), technical performance (e.g., process efficiency, productivity, technology readiness level, scalability, and operational reliability), and circularity performance (e.g., carbon utilization efficiency, nutrient recovery, waste diversion, cascade efficiency, resource circularity, and energy self-sufficiency). These dimensions could be integrated through MCDA or related decision-support methods in future comparative assessments. The framework is conceptual and was not applied as a weighted, scored, or validated decision model to the technologies reviewed in the present study.

As illustrated in Figure 10, the sustainability of food-waste biorefineries emerges from the interaction between feedstock characteristics, conversion technologies, process integration and system-level assessment.

Feedstock composition influences the suitability of available conversion pathways, whereas pretreatment, downstream processing and resource recovery affect environmental impacts, production costs and resource efficiency. Technology maturity further influences the potential transition of promising laboratory concepts toward industrial implementation [63,181]. These interactions demonstrate that no single indicator is sufficient to characterize overall biorefinery sustainability and support the use of an integrated assessment perspective [210]. Accordingly, Figure 10 presents a conceptual framework proposed from the synthesis developed in this review, rather than a validated decision model. The framework organizes the environmental, economic, technical, and circularity dimensions identified across the reviewed literature and illustrates how they could be considered jointly in future comparative assessments. The present review does not assign weights or scores to these dimensions, calculate a composite sustainability index, or apply a formal MCDA procedure to rank the technologies reviewed.

The complementary roles of the principal sustainability metrics are summarized in Table 16. LCA, TEA, circularity indicators, and technology readiness level (TRL) provide complementary information on environmental performance, economic feasibility, resource recovery and recirculation, and technology maturity, respectively. These assessment dimensions are considered here as complementary components of the proposed conceptual framework rather than as inputs to a validated composite sustainability model. Considered together, these metrics provide a broader basis for comparing alternative biorefinery configurations and identifying potential trade-offs relevant to industrial implementation.

Recent studies employ multi-criteria decision analysis (MCDA) and related decision-support approaches to simultaneously evaluate technical, environmental, economic, and resource-related criteria [5,210]. In the present review, however, MCDA is proposed only as a potential future mechanism for integrating these dimensions within a structured comparative assessment. No MCDA model was implemented, calibrated, or validated using the technologies reviewed, and Figure 10 should therefore be interpreted as a conceptual research framework that requires future application and validation using consistently defined indicators, explicit weighting procedures, and comparable technology-specific data.

Such methodologies recognize that the preferred configuration is not necessarily the one that maximizes a single performance indicator, but the one that provides an appropriate compromise among competing sustainability objectives under defined technical, economic and contextual conditions. MCDA is therefore particularly valuable for ranking alternative valorization pathways when improvements in one performance dimension are accompanied by trade-offs in others [210].

The growing availability of digital technologies may progressively strengthen integrated sustainability assessment, although the maturity and level of empirical validation differ substantially among individual approaches. Real-time sensing and process monitoring can provide operational data for process-state estimation and control, whereas more advanced approaches based on AI, digital twins, and reinforcement learning remain predominantly at the modelling, simulation, laboratory, or proof-of-concept stages for waste-biorefineries [182,183,210,243]. Digital-twin concepts are being investigated as potential platforms for linking process information with environmental and economic indicators, but their systematic application to integrated food-waste biorefineries has not yet been demonstrated at a level comparable with established conversion technologies. Similarly, reinforcement-learning approaches have shown potential for control under process uncertainty, but such results should not be interpreted as evidence of routine industrial implementation [182]. These emerging tools may ultimately support more adaptive process control and dynamic decision support under variations in feedstock composition and operating conditions; however, further pilot- and demonstration-scale validation is required before their benefits can be generalized to industrial food-waste biorefineries.

Sustainable food-waste biorefineries should therefore be evaluated as integrated production systems in which the interactions among conversion technologies and resource flows are considered collectively. Combining environmental assessment, TEA, circularity indicators, technological maturity, and multi-criteria decision-support methods can provide a more comprehensive basis for technology selection, process optimization, and scale-up [5,181]. Such integrated assessment can help identify configurations that balance resource efficiency, environmental performance, economic feasibility and technological implementation, thereby supporting the development of more resilient food-waste biorefineries within a circular and low-carbon bioeconomy.

## 9. Conclusions and Future Perspectives

Food-waste biorefineries are evolving from waste-treatment systems toward integrated platforms for the coordinated recovery of bioenergy, platform chemicals, biomaterials and recyclable nutrients. This transition reflects the broader development of the circular bioeconomy, in which unavoidable food-derived residual streams are considered potential sources of renewable carbon, energy and functional compounds. However, their valorization should remain subordinate to higher-priority uses within the food-use and waste hierarchy. Prevention should remain the first priority, followed, where applicable, by redistribution and reuse as food, and by feed or other established higher-value uses for eligible streams. Biorefinery processing should therefore be preferentially directed toward unavoidable, inedible, non-redistributable, or otherwise non-reusable residual streams and should not be assumed to provide an environmental advantage when it displaces an established food, feed, ingredient, or material application. The evidence examined in this review indicates that the sustainability of such systems depends not only on the performance of individual conversion technologies but also on their effective integration within coherent biorefinery configurations.

A central conclusion of this review is that feedstock characteristics provide an important decision basis for biorefinery design. Food-derived residual streams differ substantially in composition, moisture content, contamination, traceability, temporal variability, regulatory status, and existing uses, and these characteristics influence pretreatment requirements, feasible conversion pathways, downstream processing, product quality, and permissible valorization routes. The feedstock-oriented framework proposed in this review should therefore be interpreted as a decision principle for structuring pathway selection rather than as a quantitatively validated prescription for an optimal biorefinery configuration. Homogeneous food-processing side streams may provide favorable conditions for selective recovery of specific compounds, whereas heterogeneous post-consumer food waste may require more robust sorting, pretreatment, and conversion strategies. However, the suitability of any proposed configuration must ultimately be established for the specific application and location through site-specific mass and energy balances, environmental assessment, techno-economic analysis, market conditions, regulatory constraints, feedstock availability and variability, and consideration of existing or counterfactual feedstock uses.

Industrial streams such as whey, pomace, brewer’s spent grain, cereal residues, and other processing side streams may be classified as by-products, co-products, former foodstuffs, or wastes depending on the applicable jurisdiction, production context, and intended use. Their inclusion in the present technological assessment should therefore not be interpreted as assigning waste status to materials that may retain product or by-product status under applicable legislation.

The comparison of conventional, integrated, cascade, circular, and near-zero-waste configurations demonstrates that these concepts represent alternative and potentially complementary design strategies characterized by different degrees of process integration and resource recovery. Greater integration can improve carbon utilization, nutrient recovery, and internal recycling of water and energy, but may also increase capital requirements, energy and water demand, operational complexity, and downstream-processing requirements. Consequently, increasing the number of conversion steps, products, or internal recycling loops does not inherently improve sustainability performance. Cascade, circular, and near-zero-waste configurations should therefore not be regarded as intrinsically superior to simpler systems; their environmental and economic advantages must be demonstrated through system-level LCA and TEA under appropriate system boundaries and reference scenarios. Future development should focus on identifying the degree of integration that provides demonstrable technical, environmental, and economic benefits for the specific feedstock, process context, and intended products.

Cascade valorization provides a potentially useful design strategy for sequentially recovering biomass components according to their functionality, recoverability, and potential value. Selective recovery of bioactive compounds, proteins, lipids, or structural polysaccharides before biochemical or energy conversion may increase the value recovered from food-derived residual streams and improve resource utilization; however, these benefits cannot be assumed a priori. Additional fractionation, extraction, purification, and integration steps may increase energy and water demand, chemical consumption, capital expenditure, and operational complexity. The net benefit of cascade processing therefore requires application-specific validation using mass and energy balances, LCA, TEA, market conditions, regulatory constraints, and appropriate reference scenarios. Bioenergy technologies, particularly anaerobic digestion, remain important components of this hierarchy because of their technological maturity and their capacity to recover energy from heterogeneous or depleted residual streams. The future role of bioenergy is therefore likely to complement higher-value valorization pathways within multiproduct biorefinery configurations. Industrial implementation cannot, however, be assessed from laboratory yields or conversion efficiencies alone. The complementary use of LCA, TEA, circularity indicators and technology-readiness evaluation, supported where appropriate by multi-criteria decision analysis, provides a more comprehensive basis for comparing alternative process configurations. Such assessments are particularly important because improvements in product recovery or resource circularity may be accompanied by greater energy consumption, water demand, capital expenditure or downstream purification requirements. Establishing harmonized system boundaries, functional units and performance indicators would therefore improve comparability among studies and provide a stronger basis for technology selection and investment decisions.

Despite substantial scientific progress, several barriers continue to restrict large-scale implementation. Feedstock heterogeneity and seasonality, contamination, collection and logistics, downstream recovery costs, variable product quality and the limited technological maturity of many emerging conversion routes remain important constraints. Moreover, many promising processes continue to be evaluated predominantly under controlled laboratory conditions, limiting understanding of their robustness under realistic feedstock variability and continuous operation. Greater emphasis should therefore be placed on pilot- and demonstration-scale validation, integrated mass and energy balances, realistic TEA and LCA, and long-term process performance under industrially relevant conditions.

Digitalization may further support this transition by improving the ability of biorefineries to respond to variable feedstocks and operating conditions. Advanced sensing, process modelling, artificial intelligence and digital twins can support real-time monitoring, predictive control and adaptive allocation of biomass and resources among alternative conversion pathways. Their principal value lies in complementing established engineering approaches with data-driven decision support, particularly in complex multiproduct systems. Future research should therefore focus not only on developing digital tools but also on validating their contribution to process robustness, resource efficiency and economic performance at pilot and industrial scales.

The design principles and integrated configurations synthesized in this review should therefore not be interpreted as universally optimal process architectures. The available evidence is heterogeneous with respect to feedstock definition, process scale, system boundaries, technology maturity, economic assumptions, and environmental assessment methods, and the present review did not apply a standardized quantitative optimization or ranking procedure across all technologies and configurations. Rather, the proposed feedstock-oriented framework provides a structured basis for identifying context-appropriate options that require subsequent validation at the application and site level. Such validation should integrate mass and energy balances, LCA, TEA, technology readiness, market demand, regulatory requirements, infrastructure and logistics, and the existing uses of food-processing side streams.

The development of sustainable food-waste biorefineries requires a transition from technology-centered optimization toward system-oriented design. No single conversion technology, product or sustainability indicator can determine the context-appropriate configuration across the diversity of food-waste streams and regional conditions. Future progress will instead depend on the effective integration of feedstock characterization, selective fractionation, complementary biological, chemical and thermochemical conversion pathways, resource recovery and system-level sustainability assessment. By combining these elements within flexible and adaptive processing configurations, food-waste biorefineries can provide a technically realistic route for recovering greater value from unavoidable food-derived residual streams while improving resource efficiency, environmental performance and economic viability within a circular and low-carbon bioeconomy.

## Figures and Tables

**Table 1:** Terminology used for food-derived residual streams considered in this review.

Term	Definition Used in This Review	Key Distinction
Food loss	Food removed from the human food supply chain during production, post-harvest handling, storage, or processing, before the retail and consumer stages.	Upstream concept; distinguished from food waste generated predominantly at retail, food-service, and household stages.
Food waste	Food-derived material that has become waste under the applicable regulatory framework, particularly from retail, food-service, and household activities.	Waste status is explicit; heterogeneous post-consumer streams may require sorting, stabilization, and pretreatment.
Former foodstuffs	Products originally produced for human consumption that are no longer intended for that purpose.	Their subsequent legal status and permitted uses depend on the applicable regulatory framework and intended use.
Food-processing residues	Residual material flows generated during food production or processing that remain after the principal processing operation.	Broad technical term; does not by itself imply legal waste or by-product status.
By-products	Food-processing streams that meet the applicable legal criteria for by-product status and are therefore not classified as waste.	Legally distinct from waste; classification depends on the applicable regulatory framework.
Side streams	A broad technical term for secondary material flows generated during food production or processing that may retain potential for further use or valorization.	Does not automatically indicate waste status; may include streams with existing food, feed, ingredient, fertilizer, or industrial uses.
Mixed municipal biowaste	A heterogeneous biodegradable municipal waste stream that may contain food-derived material together with other biodegradable fractions and contaminants, depending on the collection system.	Should not be treated as equivalent to a defined food-processing stream or homogeneous food-waste feedstock.

**Table 2:** Comparative characteristics and biorefinery implications of major food-derived residual-stream categories.

Source	Typical Material Characteristics	Principal Advantages	Critical Limitations	Most Defensible Initial Valorization Options	References
Households	Highly heterogeneous mixtures of cooked food, fruit and vegetable residues, bread, meat, dairy, oils, bones, shells, and non-food contaminants; generally high moisture.	Large and continuous aggregate supply; broad carbohydrate, protein, and lipid content.	Poor traceability; variable composition; contamination; dispersed collection; rapid degradation.	Mixed-culture fermentation, anaerobic digestion, volatile fatty acid production, and energy/nutrient recovery after effective source separation.	[41,42,43,44]
Restaurants and food services	Cooked starches, fats, meat, sauces, preparation residues, and plate waste; often high soluble organic content and salinity.	Concentrated generation points; frequent collection; potentially high biodegradability.	Salt, fats, detergents, physical contaminants, pathogens, ammonia and long-chain fatty acid inhibition.	Separate recovery of waste oils; fermentation of preparation residues; controlled co-digestion or anaerobic digestion of mixed residues.	[45,46,47,48]
Supermarkets and retail	Unsold fruit, vegetables, bakery, dairy, meat, ready meals, and packaged products.	Product identity may be known; relatively intact constituents; concentrated collection.	Ethical priority for redistribution; extensive packaging; expiry-related safety issues; highly variable product mix.	Redistribution first; category-specific extraction or fermentation; anaerobic digestion of mixed de-packaged fractions.	[49,50,51,52]
Food industries	Process-specific residues, side streams, and by-products, where legally applicable, such as pomace, whey, bran, spent grain, oilseed cake, wastewater, and animal-processing residual streams.	Higher homogeneity, traceability, concentration, and potential for targeted recovery.	Existing competing uses; seasonal production; process-specific inhibitors; regulatory constraints; dilute wastewaters.	Compound recovery, targeted fermentation, enzyme or biopolymer production, followed by energy and nutrient recovery from residues.	[53,54,55,56]

**Table 3:** Comparative characteristics, potential benefits, and trade-offs of major biorefinery configurations applied to food-derived residual streams.

Biorefinery Concept	Primary Objective	Degree of Integration	Resource Recovery Strategy	Potential Benefits	Principal Limitations	References
Conventional	Maximize one product	Low	Single conversion pathway	Mature technology; simple operation	Poor utilization of biomass complexity; market dependence	[81,83]
Integrated	Coordinated production of several products	Moderate–High	Material, energy and utility integration	Improved overall efficiency; diversified revenue	Higher capital cost and operational complexity	[73,75,84]
Cascade	Sequential recovery according to product value	High	Hierarchical fractionation and conversion	Preserves molecular functionality; higher added value	Multiple separation steps; increasing processing costs	[80,85]
Circular	Close carbon, nutrient, water and energy loops	Very High	Internal recycling and industrial symbiosis	Potential for improved resource efficiency and reduced external resource demand through internal recycling	Difficult to quantify; dependent on system boundaries	[17,27,31]
Near-zero-waste/zero-waste-oriented	Minimize residual material and energy streams	Theoretical maximum	Extensive recovery, reuse, and valorization of process streams	Potential for extensive resource recovery and residual-stream minimization	Thermodynamic limitations; potentially high energy, water, capital, and processing requirements; economic and market constraints	[82,86]

Note: The degree of integration shown in this table describes process configuration and resource-flow coordination and should not be interpreted as a sustainability ranking. Greater integration or resource recovery does not necessarily result in lower environmental impacts or higher economic performance; these outcomes require case-specific system-level assessment using LCA and TEA.

**Table 4:** Availability of system-level performance indicators reported for representative food-waste and food-derived residual-stream biorefinery studies.

Representative Configuration/Study	Feedstock	Main Products/Recovery Routes	Scale/Assessment Basis	Carbon Recovery	Resource/Energy Efficiency	GHG Performance	Water Use/ Recovery	Techno-Economic Performance	References
Cascade sugar-recovery biorefinery	Wet food waste	Soluble sugars; starch-derived sugars; structural-carbohydrate-derived sugars	Process design + optimization; TEA + system-level LCA; basis: 1 MT wet FW	NR	Extraction efficiencies: 81.4% soluble sugars; 88.5% starch-derived sugars; 91.5% structural-carbohydrate-derived sugars	−68 kg CO_2_/MT wet FW	NR	MSP: US$358/MT sugar (most cost-effective soluble + starch-sugar scenario)	[87]
Integrated PHA–bioenergy biorefinery	Food waste	PHA from hydrolysate; biogas from residual solids	Laboratory integrated process; ultrasonic-assisted alkaline pretreatment (UAP)	NR	UAP: 21.13% solubilization at 834.8 kJ/kg TS; cumulative biogas yield: 530 mL/g COD; PHA yield: 0.4811 g/g	NR	NR	Positive net profit: US$6.81/t FW for UAP-based configuration	[88]
Modular multi-feedstock PHA bioprocess	Food waste + fats/oils/grease + glycerol waste	PHA via three integrated microbial routes	Modeled modular system; 450 MT/day feedstock; 60 MT/day PHA; TEA + cradle-to-grave LCA	NR	NR	GWP: −2.71 kg CO_2_-eq/kg PHA	NR	FCI: US$110 million; MSP: US$3.64/kg PHA	[89]
Food-waste-digestate-to-biochar integrated HTC–post-carbonization system	Food-waste anaerobic digestate	Hydrochar/biochar; long-term carbon storage; agricultural application	LCA–TEA; integrated HTC + post-carbonization; commercial-scale model informed by demonstration facility	~80% of initial digestate carbon retained in hydrochar after HTC; 88% stable carbon fraction assumed in biochar	NR	Biochar stores 1.7 t CO_2_-eq/t biochar; net GHG removal: 1.15–1.20 t CO_2_-eq/t biochar	NR	CAPEX: £9.5 M; base-case biochar production cost: £88/t biochar; £183.9/t with digestate transport	[90]
Multiproduct food-waste biorefinery	Food waste	Hydrogen; ethanol; organic fertilizer and other by-products	Designed plant: 100 t FW/day (33,000 t/year); techno-economic assessment	NR	Annual outputs: 2,465,100 m^3^ H_2_; 6111.6 t ethanol; 5362.5 t organic fertilizer	NR	NR	TCI: US$7,625,549; AOC: US$2,432,290.7/year; net profit: US$3,141,867.6/year; PBP: 3.5 y; IRR: 45.54%; ROI: 43.88%	[91]
Restaurant-food-waste-to-energy systems	Restaurant food waste	Biogas/electricity; comparison of AD, pyrolysis and gasification	Comparative TEA + LCA; Malaysia/Singapore; medium-scale AD scenario	NR	Biogas-production efficiency: 85%; waste-volume reduction: 60%; renewable electricity: up to 5 MW	AD: −0.8 kg CO_2_-eq/kg FW vs. landfill; reported ~60% carbon-emission reduction	NR	Electricity generation cost: US$0.08/kWh; ROI: 12–15%; payback: 5–7 y	[92]
Industrial-scale n-caproate cascade biorefinery	Source-collected food waste	Mixed MCCAs/n-caproate; residual broth as alternative carbon source for wastewater N removal	Industrial plant; 150 t FW/day pretreatment + CCE capacity; one-year stable operation	NR	n-Caproate volumetric production rate: 3.8 kg/(m^3^·d); process operated without external heat, NaOH or ethanol addition	NR (study reports reduction, but no single harmonized system-level GHG value in the reported summary)	NR (tap water used for cleaning; no system-level recovery metric reported)	Direct operating cost: US$0.32–0.36/kg n-caproate; avoided chemicals/heat save US$1.1–2.6/kg; estimated ED-PS purification: US$0.23–0.58/kg	[93]

Note: NR = not reported as a quantitative system-level indicator in the cited primary study. Values are retained in the original study-specific units and methodological bases and should not be interpreted as directly comparable across rows. Process-level yields, extraction efficiencies, energy inputs, or production rates are shown only when explicitly reported and are not reinterpreted as whole-system carbon, nutrient, water, or resource-recovery efficiencies. The table is intended to demonstrate both the availability and the current lack of harmonization of system-level indicators, not to rank the configurations.

**Table 5:** Critical comparison of physical pretreatment technologies for food-waste biorefineries.

Technology	Primary Mechanism	Main Advantages	Major Limitations	Energy Demand	Environmental Impact	Typical Applications	Technology Readiness	References
Milling	Particle-size reduction; increased surface area	Simple, scalable; improves mixing and hydrolysis	High electricity demand; diminishing returns	High	Depends on electricity source	Anaerobic digestion, fermentation, enzymatic hydrolysis	Commercial	[95,97,99]
Drying	Moisture removal	Improves storage and transport; suitable for thermochemical routes	High thermal demand; may degrade bioactives	Very high	Potentially high; strongly dependent on heat/energy source	Extraction, pyrolysis, storage	Commercial	[97,100,101]
Steam explosion	High-pressure steam followed by rapid depressurization	Excellent disruption of lignocellulosic structure	Potential inhibitor formation; high capital cost	Moderate–High	Relatively low; further improved by heat integration	Food-processing residues rich in fiber	Pilot–Commercial	[102,103,104]
Ultrasonication	Cavitation and cell disruption	Enhances solubilization and extraction	Scale-up and energy challenges	Moderate–High	Low chemical burden but electricity intensive	Bioactive extraction; digestion enhancement	Pilot–early commercial	[105,106,107]

**Table 6:** Comparative assessment of chemical pretreatment technologies for food-waste biorefineries.

Technology	Typical Reagents/Solvents	Primary Action	Key Advantages	Major Limitations	Inhibitors/Downstream Risks	Recovery and Recycling Needs	Environmental Implications	Technology Readiness (TRL)	Most Suitable Food-Waste Streams	References
Acid pretreatment	Dilute or concentrated H_2_SO_4_, HCl, H_3_PO_4_; organic acids	Hydrolyses hemicellulose and opens the lignocellulosic matrix	Rapid; inexpensive reagents; high pentose release; mature equipment base	Corrosion; neutralization demand; salt-rich effluents; possible sugar degradation	Furfural, HMF, phenolics and weak acids can inhibit fermentation and anaerobic digestion	Acid recovery is desirable but often difficult; neutralization and detoxification may be required	Potentially high due to reagent manufacture, corrosion-resistant equipment, water use and wastewater treatment	Pilot-commercial for lignocellulosic biomass; feedstock-specific for food waste TRL = 6–8	Fruit peels, cereal residues, brewer’s spent grain and other hemicellulose-rich residues	[95,111,112]
Alkali pretreatment	NaOH, KOH, Ca(OH)_2_, ammonia	Removes or redistributes lignin, breaks ester bonds and swells cellulose	Selective delignification; generally fewer furans; improves enzymatic accessibility	High alkali consumption; long residence times in some systems; intensive washing and neutralization	Residual salts, elevated pH and dissolved lignin can interfere with downstream processing	Chemical recovery and water recycling are important to control cost and effluent generation	Moderate to high; burden dominated by reagent use, washing water and alkaline wastewater	Pilot–commercial for lignocellulosic biomass; feedstock-specific demonstration for food-processing residues, TRL = 6–8	Vegetable stems, cereal by-products, fibrous peels and lignin-rich processing residues	[95,113,114,115]
Ionic liquids	Imidazolium-, ammonium- or phosphonium-based ionic liquids	Dissolves cellulose, reduces crystallinity and enables selective fractionation	High deconstruction efficiency; tunable properties; strong cellulose accessibility	High solvent cost; viscosity; sensitivity to water; complex product separation	Residual ionic liquid can inhibit enzymes and microorganisms; toxicity varies by structure	Very high recovery efficiency is essential; solvent purification and reuse dominate economics	Uncertain and highly formulation-dependent; solvent synthesis and losses can outweigh low volatility benefits	Laboratory-pilot; limited industrial deployment TRL = 4–5	Highly recalcitrant lignocellulosic food-processing residues where high-value fractionation can justify cost	[97,116,117]
Deep eutectic solvents	Choline chloride with organic acids, polyols, sugars or other hydrogen-bond donors	Selective lignin solubilization, matrix disruption and mild fractionation	Simpler preparation; potentially lower cost and toxicity; broad tunability; compatibility with bioactive recovery	High viscosity; mass-transfer limitations; incomplete scale-up evidence; variable water sensitivity	Residual solvent may affect enzymes or fermentation; thermal degradation and impurity effects are insufficiently characterized	Recycling, component stability and low-energy separation remain unresolved at scale	Potentially lower than ionic liquids, but claims of greenness require full life-cycle verification	Laboratory-early pilot TRL = 4–5	Bioactive-rich peels, fruit and vegetable residues, cereal by-products and other fractionation-oriented feedstocks	[97,105,118]

Abbreviations: HMF, 5-hydroxymethylfurfural. Readiness levels are indicative and vary with feedstock, solvent-recovery configuration and target product. Environmental performance should be verified through process-specific life-cycle and techno-economic assessment.

**Table 7:** Characteristics and applications of major biological pretreatment technologies for food-waste biorefineries.

Biological Pretreatment	Main Biological Agent	Preferred Food-Waste Substrates	Principal Advantages	Major Limitations	TRL	Most Suitable Downstream Processes	Current Research Priorities	References
Fungal pretreatment	White-rot fungi (*Phanerochaete*, *Pleurotus*, *Trametes*, *Ceriporiopsis*)	Lignocellulosic residues, cereal by-products, fruit and vegetable processing wastes	Selective lignin degradation; improved enzymatic accessibility; low inhibitor formation	Long residence time; carbohydrate losses; sensitive process control	4–6	Enzymatic hydrolysis, bioethanol, anaerobic digestion	Strain improvement, co-cultivation, metabolic engineering, hybrid pretreatment	[123,124]
Enzymatic pretreatment	Cellulases, hemicellulases, pectinases, amylases, proteases, lipases	Feedstocks with well-characterized composition; food-processing side streams	High substrate specificity; mild operating conditions; preservation of valuable compounds	High enzyme cost; enzyme inhibition; recycling challenges	5–7	Fermentation, organic acids, bioethanol, SCP, nutraceuticals	Enzyme cocktails, immobilization, enzyme recycling, on-site enzyme production	[95,125,126]
Microbial pretreatment	Bacterial cultures and mixed microbial consortia	Heterogeneous municipal food waste; wet organic residues	Simultaneous hydrolysis and production of VFAs or soluble intermediates; low chemical input	Variable microbial communities; long retention times; process instability	5–6	Anaerobic digestion, VFA production, chain elongation, hydrogen fermentation	Synthetic consortia, microbiome engineering, real-time monitoring, AI-assisted process control	[127,128,129]

**Table 8:** Comparison of major biofuel production pathways from food waste.

Product	Main Feedstock	Conversion Process	Microorganisms	Typical Yield	Major Advantages	Major Limitations	TRL	References
Bioethanol	Bread and bakery waste; discarded rice, pasta, potato and confectionery residues; fruit-processing and beverage wastes; carbohydrate-rich food-waste hydrolysates	Sorting and size reduction; substrate-specific pretreatment; enzymatic hydrolysis of starch or structural carbohydrates; SHF, SSF or consolidated bioprocessing; distillation and dehydration	*Saccharomyces cerevisiae*; *Kluyveromyces marxianus*; *Zymomonas mobilis*; engineered amylolytic or pentose-fermenting strains	Typically 0.35–0.50 g ethanol/g fermentable sugar (about 70–98% of theoretical); commonly 20–100 g/L, with higher titers for concentrated bakery wastes	Mature fermentation platform; high conversion of homogeneous sugar- and starch-rich residues; transportable liquid fuel; compatible with existing blending infrastructure	Feedstock heterogeneity; enzyme and pretreatment costs; inhibitor formation; contamination; poor pentose utilization by conventional yeast; distillation penalty at low ethanol titers	6–8, Pilot–demonstration; commercial fermentation technology, but food-waste-specific deployment remains feedstock-dependent	[26,64,133]
Biogas	Source-separated household and catering food waste; supermarket residues; food-processing slurries; mixed carbohydrate-, protein- and lipid-rich organic wastes	Anaerobic hydrolysis, acidogenesis, acetogenesis and methanogenesis; single- or two-stage digestion; gas cleaning or upgrading; digestate separation and nutrient management	Hydrolytic and acidogenic bacteria (e.g., Clostridium, Bacteroides); syntrophs (*Syntrophomonas*, *Syntrophobacter*); methanogenic archaea (*Methanothrix*, *Methanosarcina*, *Methanobacterium*, *Methanoculleus*)	Commonly 300–550 mL CH4/g VS added in favorable batch tests; full-scale yields are generally lower and depend strongly on loading, retention time and feedstock variability	Commercially established; handles wet and heterogeneous substrates; converts carbohydrates, proteins and lipids; dispatchable energy; suitable as a terminal step for residual carbon recovery	Acidification, ammonia and long-chain-fatty-acid inhibition; slow methanogenic recovery; methane leakage; gas-upgrading costs; large digestate volumes and uncertain fertilizer markets	8–9	[65,69,134]
Biohydrogen	Carbohydrate-rich bakery, rice, potato, fruit, confectionery and beverage wastes; hydrolyzed source-separated food waste; acidogenic fermentation effluents for secondary conversion	Dark fermentation, optionally followed by photofermentation, microbial electrolysis or anaerobic digestion; inoculum conditioning; gas separation; downstream volatile-fatty-acid valorization	*Clostridium butyricum*, *C. beijerinckii*, *Enterobacter* spp., *Thermoanaerobacterium* spp.; photofermentative *Rhodobacter* and *Rhodopseudomonas* spp.; mixed acidogenic consortia	Dark fermentation commonly about 1–3 mol H2/mol hexose or roughly 50–200 mL H_2_/g VS; integrated systems can increase cumulative H2 recovery but often at lower volumetric productivity	Rapid conversion under mild conditions; no light required for dark fermentation; produces hydrogen plus platform volatile fatty acids; suitable as an upstream acidogenic stage	Low standalone hydrogen yield; hydrogen consumption by methanogens and homoacetogens; gas purification and storage challenges; unstable product spectrum; photofermentation requires large illuminated area and low-turbidity effluent	3–5	[135,136,137]
Biodiesel (waste cooking oil)	Waste cooking oils and recovered native lipids from restaurants, institutional kitchens, food processors and lipid-rich catering residues	Filtration, dewatering and acidity reduction; acid esterification when free fatty acids are high; base-, acid-, heterogeneous- or enzyme-catalyzed transesterification; glycerol separation and ester purification	No production microorganism required for chemical conversion; lipases from *Candida antarctica*, *Rhizomucor miehei* and related organisms may be used in enzymatic routes	Frequently > 90–95% ester conversion under optimized conditions; practical yield depends on oil quality, pretreatment losses, alcohol recovery and purification efficiency	Mature reaction technology; concentrated, energy-dense feedstock; reduces dependence on virgin edible oils; compatible with diesel blending; high near-term deployment potential	Finite and dispersed feedstock supply; variable water and free-fatty-acid contents; soap formation; methanol and catalyst requirements; wastewater generation; crude-glycerol management; cold-flow/oxidative-stability trade-offs	8–9	[138,139,140,141]
Biodiesel (microbial lipids)	Food-waste hydrolysates, crude glycerol, volatile-fatty-acid streams, digestate nutrients and other low-cost carbon-rich process residues	Pretreatment and hydrolysis; aerobic cultivation; biomass harvesting and cell disruption; lipid extraction or in situ transesterification; biodiesel purification and residual-biomass valorization	Oleaginous yeasts such as *Yarrowia lipolytica*, *Rhodotorula*/*Rhodosporidium toruloides* and *Cutaneotrichosporon oleaginosum*; microalgae such as *Chlorella*, *Scenedesmus* and *Nannochloropsis* spp.	Oleaginous yeasts commonly accumulate 20–60% lipid of dry biomass; microalgae often 15–50%; reported lipid titers range from a few g/L to >20 g/L in optimized fed-batch cultures	Converts non-lipid food-waste carbon into oil; avoids agricultural land; tunable fatty-acid composition; opportunities for co-production of carotenoids, proteins, PUFAs and oleochemicals	Aeration and mixing demand; variable C:N ratio; low biomass concentration; costly harvesting, drying, disruption and extraction; solvent recovery; limited pilot-scale evidence; fuel value often insufficient without high-value co-products	3–5	[138,139,140]

Note: Reported yields are presented using the basis and units provided in the cited studies. Yield values expressed on different feedstock bases, particularly dry-matter and wet-matter bases, should not be compared directly without appropriate conversion. Where the reporting basis is not explicitly available, no conversion has been introduced.

**Table 9:** Comparison of major organic acids produced from food waste.

Organic Acid	Main Feedstock	Conversion Process	Major Microorganisms	Typical Yield *	Major Applications	Major Advantages	Major Limitations	TRL	References
Lactic acid	Bakery waste, potato waste, dairy waste, fruit & vegetable waste, confectionery waste, mixed carbohydrate-rich food waste	Enzymatic hydrolysis followed by anaerobic/microaerophilic fermentation	*Lacticaseibacillus casei*; *Lactiplantibacillus plantarum*; *Bacillus coagulans*; *Pediococcus acidilactici*; engineered *E. coli*	0.85–0.98 g/g fermentable sugar; typically 40–120 g/L using food waste	PLA bioplastics, food acidulant, pharmaceuticals, cosmetics, green solvents	High yield, mature technology, expanding PLA market, mild pretreatment	Costly downstream purification, neutralization chemicals, gypsum generation, product inhibition	6–8 (pilot–demonstration; fermentation technology itself is commercial)	[24,142,143]
Succinic acid	Bakery waste, fruit waste, cassava residues, potato waste, dairy waste, food waste hydrolysates	Anaerobic fermentation with CO_2_ fixation through reductive TCA pathway	*Actinobacillus succinogenes*; *Basfia succiniciproducens*; engineered *Escherichia coli*; *Corynebacterium glutamicum*	~0.5–0.9 g/g fermentable sugar; typically ~25–80 g/L for waste-derived substrates, although >100 g/L can be achieved under optimized fermentation conditions	PBS, 1,4-butanediol, tetrahydrofuran, solvents, pharmaceuticals	High-value platform chemical, CO_2_ utilization, versatile precursor	High downstream recovery cost, by-products, CO_2_ requirement, acid inhibition	6–8 (TRL refers to the maturity of bio-based succinic acid fermentation; maturity specifically for food-waste-derived feedstocks is generally lower and feedstock-dependent).	[144,145,146]
Citric acid	Citrus waste, fruit-processing residues, apple pomace, bakery waste, potato waste, confectionery waste, sugar-rich food residues	Aerobic fungal fermentation under nitrogen-limited conditions	*Aspergillus niger*; *Aspergillus awamori*; *Yarrowia lipolytica*; *Candida* spp.	Highly feedstock- and process-dependent; waste-based systems commonly report lower titers than optimized sugar-based fermentation, although high conversion efficiencies can be achieved	Food additives, beverages, pharmaceuticals, detergents, cosmetics, chelating agents	Highly mature technology, broad market, excellent productivity	High aeration demand, gypsum generation, oxygen-transfer limitations, trace-metal sensitivity	9 (TRL reflects the maturity of the core fermentation technology; maturity of food-waste-based processes remains feedstock- and configuration-dependent).	[147,148]

Note: * Typical yields are representative ranges reported in the literature and vary with food-waste composition, pretreatment, fermentation conditions, microbial strain, and the basis used for yield calculation. Actual performance depends on feedstock composition, pretreatment efficiency, microorganism, fermentation strategy, and downstream processing. TRL = Technology Readiness Level (1–9), where 9 represents fully commercialized technology. Reported yields are presented using the basis and units provided in the cited studies. Yield values expressed on different feedstock bases, particularly dry-matter and wet-matter bases, should not be compared directly without appropriate conversion. Where the reporting basis is not explicitly available, no conversion has been introduced.

**Table 10:** Comparison of high-value products derived from food waste.

Product	Feedstock	Main Microorganisms/Production Route	Commercial Application	Relative Market Value	TRL	Main Commercialization Challenges	References
Industrial enzymes	Starch-rich bakery waste; fruit and vegetable residues; cereal bran; oilseed cakes; dairy and lipid-rich wastes	*Aspergillus niger*; *A. oryzae*; *Trichoderma reesei*; *Bacillus subtilis*; *B. licheniformis*; *Yarrowia lipolytica*	Food processing; detergents; textiles; pulp and paper; feed; on-site hydrolysis in biorefineries	High	6–8 (TRL refers to enzyme production using food/agri-food waste as fermentation substrate; conventional industrial enzyme production itself is commercially mature (TRL 9))	Variable enzyme activity; contamination control; aeration and drying costs; recovery and formulation; competition with established enzyme platforms	[156,157,158]
Polyhydroxyalkanoates (PHA)	Sugar- and starch-rich hydrolysates; waste oils; volatile fatty acids from acidogenic fermentation; dairy and mixed food wastes	*Cupriavidus necator*; *Pseudomonas putida*; *Bacillus megaterium*; *Halomonas bluephagenesis*; mixed microbial cultures	Biodegradable packaging; agricultural films; coatings; medical materials; drug delivery; 3D-printing polymers	High	5–8	High downstream-recovery cost; polymer-quality variability; sterilization and aeration demand; inconsistent feedstocks; competition with low-cost petroplastics	[159,160,161]
Biosurfactants	Waste cooking oil; dairy fats and whey; molasses; fruit-processing residues; bakery waste; crude glycerol	*Starmerella bombicola*; *Bacillus subtilis*; *B. licheniformis*; *Pseudomonas putida*; *Yarrowia lipolytica*	Detergents; cosmetics; food emulsifiers; pharmaceuticals; bioremediation; enhanced oil recovery; agricultural formulations	High to very high	5–8 (TRL reflects the maturity of biosurfactant production using food-waste-derived substrates; maturity varies considerably among biosurfactant classes, producer strains and feedstocks, with selected products approaching commercial deployment while many waste-based routes remain at laboratory or pilot scale).	Low volumetric productivity for some products; costly purification; foam control; pathogenicity of some producers; product-composition variability	[96,162,163]
Single-cell proteins/microbial biomass	Food-waste hydrolysates; whey; bakery and cereal residues; fruit and vegetable wastes; brewery by-products; volatile fatty acids	*Saccharomyces cerevisiae*; *Cyberlindnera jadinii*; *Kluyveromyces marxianus*; *Yarrowia lipolytica*; *Fusarium venenatum*; *microalgae*	Aquaculture, poultry, livestock and pet feed; protein concentrates; meat analogues; functional-food ingredients	Medium to high	6–9 (TRL refers specifically to SCP production using food-waste-derived substrates; conventional SCP production from standardized substrates includes commercially established processes, whereas waste-based routes remain feedstock- and application-dependent)	Feedstock safety and traceability; nucleic acid reduction; harvesting and drying cost; digestibility and palatability; regulatory and consumer acceptance	[164,165,166]
Nutraceuticals	Fruit peels, seeds and pomace; cereal bran; oilseed cakes; whey and fish-processing residues; vegetable trimmings	Primarily extraction-based; proteolytic *Bacillus*, *Lactobacillaceae*, *Aspergillus* and *yeast* cultures used for peptide release or biotransformation	Functional foods; dietary supplements; natural antioxidants and preservatives; cosmetics; pharmaceutical ingredients	Very high	4–8 (TRL varies substantially according to compound class, extraction or bioconversion route and intended application; established food-waste-derived extracts may approach commercial deployment, whereas novel bioactive compounds and peptides often remain at laboratory or pilot stage)	Low and variable target-compound concentrations; stabilization and purification costs; limited bioavailability and clinical validation; strict safety and health-claim requirements	[7,94,167]
Natural pigments	Tomato, carrot, pumpkin and pepper residues; grape and berry pomace; beetroot and pitaya wastes; food-waste hydrolysates for fermentation	*Rhodotorula* spp.; *Xanthophyllomyces dendrorhous*; *Monascus* spp.; *Talaromyces* spp.; engineered *Yarrowia lipolytica*; *microalgae* and *cyanobacteria*	Food and feed colorants; nutraceuticals; cosmetics; active and intelligent packaging; textile and pharmaceutical applications	High to very high	4–8	Sensitivity to light, oxygen, heat and pH; inconsistent hue and concentration; extraction and encapsulation costs; toxin or pathogenicity risks for selected microbial producers	[94,168,169]
Animal feed	Source-separated fruit and vegetable residues; bakery waste; spent grains; dairy and cereal by-products; oilseed cakes; legally permitted food-processing residues	*Lactiplantibacillus plantarum*; *Pediococcus* spp.; *Bacillus subtilis*; *Saccharomyces cerevisiae*; *Aspergillus oryzae*; black soldier fly larvae	Silage; fermented feed; insect meal and oil; microbial protein; aquaculture, poultry, livestock and companion-animal diets	Low to medium	6–9 (TRL varies substantially with feedstock and conversion route; established food-processing by-products are commercially used as feed ingredients, whereas insect- and fermentation-based conversion of heterogeneous food waste exhibits lower and more variable technological maturity)	Regulatory restrictions; pathogens, mycotoxins and chemical contaminants; nutritional variability; transport and drying costs; need for validated animal-performance data	[169,170,171]

Note Relative market value is qualitative and reflects the typical unit-value potential of the products, without representing their total market size. TRL values are approximate ranges because readiness varies with feedstock, product purity, organism, process configuration, and application. TRL = technology readiness level.

**Table 11:** European Commission technology readiness level (TRL) framework and its application to food-derived residual-stream biorefineries [180,181,184].

Technology Readiness Level	Development Stage	Interpretation for Food-Waste Biorefineries
TRL 2–3	Proof of concept	Scientific feasibility demonstrated under laboratory conditions. Research focuses on substrate suitability, microbial or catalytic performance, and preliminary process development.
TRL 4–5	Laboratory validation and early pilot testing	Integrated processes validated under controlled conditions. Process optimization, downstream recovery and operational stability become major research priorities.
TRL 6–7	Pilot and demonstration scale	Technologies demonstrated under industrially relevant conditions with increasing process integration. TEA and LCA become essential for further development.
TRL 8–9	Commercial implementation	Technologies successfully deployed at industrial scale with established supply chains, process control and commercial markets. Future improvements focus primarily on process optimization and cost reduction, building on the technological feasibility already demonstrated.

Note: TRL is assigned to a specific technology demonstrated for a defined feedstock, process configuration, product, scale, and operational environment. The categories shown here provide the framework used to interpret the application-specific evidence reviewed in this study and should not be used to assign maturity generically to an entire product class.

**Table 13:** Scientific rationale, technological approaches, and implementation considerations for integrated food-waste biorefineries.

Design Principle	Scientific Rationale	Industrial Implication	Representative Technologies/Approaches	Recommended Stage of Application	Expected Benefits	References
Feedstock characterization and fractionation	Food waste is heterogeneous; early characterization enables selective processing.	Feedstock-specific design and improved product quality.	AI sorting; optical sensors; membrane separation; mechanical fractionation; solvent extraction	All development stages, from initial process design to commercial operation	Improved recovery; reduced contamination; better quality	[70,154,217]
Sequential cascade valorization	Recover high-value compounds before lower-value products.	Multiple revenue streams.	Bioactive extraction → protein recovery → fermentation → anaerobic digestion	Process development, pilot, demonstration, and commercial operation	Higher economic return	[79,88]
Feedstock-specific process selection	Different wastes require different pathways.	Avoids one-size-fits-all processing.	Feedstock-specific cascades	All development stages, from initial process design to commercial operation	Higher efficiency	[6,78]
Product-oriented design	Selection based on product value, market demand, process feasibility, and technological maturity.	Flexible product portfolio.	Nutraceuticals; platform chemicals; biopolymers; biofuels	Process development, pilot, demonstration, and commercial implementation	Improved profitability	[204,205]
Integration of processes	Complementary technologies maximize utilization.	Residual streams become feedstocks for subsequent processing.	Fermentation; anaerobic digestion; pyrolysis; hydrothermal carbonization; gasification	Process development, pilot, demonstration, and commercial operation	Higher biomass utilization	[78,88]
TEA-guided optimization	Economic performance must be assessed beyond laboratory yields.	Supports process optimization and investment decisions.	Process simulation; sensitivity analysis; scenario analysis	Early process development through commercial implementation	Lower costs; improved economic feasibility	[181,204]
LCA-guided design	Assess full life-cycle impacts and identify environmental hotspots.	Supports environmentally informed technology and process selection.	Life-cycle assessment; carbon footprint; water footprint	Process development through commercial implementation	Reduced environmental burdens; identification of impact hotspots	[87,181]
Circular resource integration	Recycle heat, water, nutrients, and CO_2_ within the biorefinery.	Reduces external resource demand and improves internal resource recovery.	Heat integration; water recycling; nutrient recovery; CO_2_ reuse	Pilot, demonstration, and commercial operation	Improved circularity and resource efficiency	[70,218]
AI-assisted control	Potential adaptation to variable feedstocks and operating conditions.	Emerging support for predictive monitoring and adaptive process control; industrial validation remains limited.	Machine learning; digital twins; online sensors; reinforcement-learning control	Process development and pilot validation, with future potential for demonstration-scale integration	Potential improvements in process robustness, monitoring, and adaptive control	[182,183]
Scalability and modularity	Biorefinery configurations should accommodate regional, seasonal, and operational variability.	Facilitates scale-up, capacity adjustment, and decentralized or regional implementation.	Modular biorefineries; scalable process units	Pilot, demonstration, scale-up, and commercial deployment	Improved industrial scalability and flexibility	[219,220]

**Table 14:** Methodological characteristics and principal conclusions of representative life-cycle assessments of food-derived residual-stream valorization and biorefinery systems.

Study	Feedstock and Valorization Pathway	Functional Unit	System Boundary	Allocation/Substitution Approach	Reference Scenario/Comparison	Main Impact Categories	Principal Conclusion and Methodological Influence
[168]	Food waste or sewage sludge → commercial-grade volatile fatty acids (VFAs), with downstream purification and residual-stream digestion	1 t waste valorized; 1 t VFAs produced	Cradle-to-gate; foreground facility plus background chemicals, energy, and transport	Attributional, bottom-up LCI; no upstream burden assigned to the waste feedstock; multifunctionality examined through both waste- and product-based functional units	Food-waste versus sewage-sludge VFA production; sensitivity to feedstock transportation	Climate change, ozone depletion, acidification, freshwater and marine eutrophication, water consumption, fossil and mineral resource scarcity	The preferred scenario changed with the functional unit: sewage sludge performed better per tonne of waste, whereas food waste performed substantially better per tonne of VFAs because of its higher VFA yield. Transportation and chemical demand were major hotspots, demonstrating that FU and boundary assumptions can change the comparative conclusion.
[222]	Sewage sludge co-digestion with food waste or fat–oil–grease in a wastewater-treatment plant	Plant-wide wastewater-treatment basis used for comparison of digestion scenarios	Plant-wide LCA including wastewater treatment, anaerobic digestion, energy recovery, residual management, and alternative handling of co-substrates	Avoided-management effects were included in scenario definition; food waste or FOG not co-digested in a given scenario was assigned an alternative management route	Sludge mono-digestion versus co-digestion with food waste or FOG; food-waste landfilling represented an important counterfactual	ReCiPe 2016 midpoint categories, including global warming, eutrophication, acidification, toxicity/ecotoxicity, resource scarcity, and water consumption	Mono-digestion showed the least favorable environmental performance. Food-waste co-digestion benefited from avoided landfilling, while FOG co-digestion benefited from higher energy recovery. The result illustrates the strong influence of the reference waste-management scenario and avoided burdens.
[223]	Bread waste → fermentative lactic acid; neutral-pH and low-pH fermentation with/without pinch-based heat integration	1 kg lactic acid	Cradle-to-gate	Product-based comparison of alternative process configurations; environmental effects evaluated for fermentation and downstream-processing alternatives	Neutral-pH versus low-pH fermentation; with versus without pinch-based heat integration	ReCiPe 2016 midpoint indicators; global warming potential used prominently in comparative interpretation	Low-pH fermentation was environmentally preferable to neutral-pH fermentation, and pinch-based heat integration further reduced impacts. Wastewater from anaerobic digestion and cooling-water demand during esterification were important hotspots, showing that process configuration and energy integration materially affect the LCA conclusion.
[224]	Apple pomace → second-generation bioethanol; vinasse managed through anaerobic digestion/biogas, lagooning, or composting	1 kg bioethanol; results also expressed per 1 L and 1 MJ	Cradle-to-biorefinery-gate, including apple cultivation and processing, bioethanol production, and vinasse treatment	Economic allocation between concentrated apple juice and apple pomace (99.7%/0.3%); burdens from vinasse treatment allocated to bioethanol	Bioethanol + biogas versus bioethanol + lagooning versus bioethanol + composting	ReCiPe 2016, 18 midpoint categories; cumulative energy demand also evaluated	Integrating anaerobic digestion of vinasse and biogas production reduced the carbon footprint markedly relative to lagooning or composting. The study also shows how economic allocation assigns only a small upstream burden to apple pomace, while the chosen vinasse-management boundary strongly affects the environmental profile.
[225]	Food waste → lactic acid using alternative neutralizing agents (seashell-derived material, NaOH, or Ca(OH)_2_)	1 kg lactic acid	Cradle-to-gate; transport, product use, and end-of-life excluded	Scenario comparison on an equivalent product basis; background inventories from Ecoinvent, Agri-footprint, and USLCI	Alternative lactic-acid neutralization routes; comparison with commercial lactic acid	IPCC 2021 GWP100 and cumulative energy demand	The environmental ranking depended on the neutralizing agent and associated upstream burdens rather than on fermentation yield alone. Comparison with commercial lactic acid highlights the importance of defining an equivalent product reference and consistent background inventories.
[226]	Source-separated household food waste → bio-oil, bioethanol, and biogas in a demonstration-scale integrated biorefinery	1 t wet source-separated food waste	Gate-to-gate, from reception of wet food waste to production of three energy co-products	System expansion/substitution credits: bio-oil substitutes rapeseed oil; bioethanol substitutes maize ethanol; biogas-CHP electricity substitutes marginal biogas electricity	Gross process impacts versus net impacts after avoided-product credits	ReCiPe 2016 Midpoint (H), 18 impact categories	System expansion materially changes the net environmental profile because credits for displaced products offset part of the processing burden. The study demonstrates that avoided-product assumptions and background electricity/energy choices are decisive when interpreting multiproduct food-waste biorefineries.

**Table 15:** Representative techno-economic assessments of food-derived residual-stream valorization and biorefinery systems.

Study	Feedstock and Conversion Pathway	Scale/Processing Capacity	Main Product(s)	Economic Basis and Currency Year	Key Quantitative Economic Indicators	Sensitivity/Major Cost Drivers	Principal Economic Conclusion
[89]	Heterogeneous food waste, fats/oils/grease, and waste glycerol → modular fermentation and PHA recovery	450 metric t feedstock/day; approximately 60 metric t PHA/day	Polyhydroxyalkanoates (PHA)	USD; currency base year not explicitly reported in the accessible study information	Fixed capital investment: approximately US$110 million; minimum selling price: US$3.64/kg PHA	Feedstock cost, solvent use, and PHA yield were identified as important performance drivers	The modular multi-feedstock configuration was reported as technically and economically promising, but the MSP remained strongly dependent on feedstock, extraction/solvent requirements, and PHA yield.
[90]	Food-waste anaerobic-digestion digestate → hydrothermal carbonisation followed by high-temperature post-carbonisation	20 kt/year biochar-production facility considered; CAPEX informed by a 10 kt/year demonstration facility	Biochar/carbon-dioxide-removal service	GBP; cost inputs drawn from multiple source years; 5% discount rate; 20-year plant lifetime	CAPEX: £9.5 million; base-case biochar production cost: £88/t biochar; with digestate transport: £183.9/t biochar; break-even gate fee > £74/t digestate for on-site production; GHG-removal cost < £100/t CO_2_-eq under favorable gate-fee conditions	Gate fee, CAPEX, fixed OPEX, digestate and biochar transport, and biochar stability; transport is particularly important because of high digestate moisture	Economic viability depended strongly on receiving a digestate gate fee and minimizing transport. Co-location with anaerobic-digestion facilities substantially improved the economics.
[228]	Digestate from full-scale continuous dry anaerobic digestion of the organic fraction of municipal solid waste → alternative digestate-management configurations	400 t/day digestate-management system	Managed digestate/recovered value associated with the AD system	USD; currency base year not explicitly reported in the accessible study information	CAPEX: US$4.2–9.8 million across scenarios; direct thermal drying OPEX: approximately US$2.0 million/year; income: US$22.6/t OFMSW for the baseline versus US$16.6/t OFMSW for direct thermal drying; electricity selling price of 15.7 US cents/kWh required for a 6-year payback in the baseline scenario	Digestate-treatment configuration, capital intensity, operating cost, electricity selling price, and availability of spare wastewater-treatment capacity	The least capital-intensive dewatering configuration gave the strongest economic result under the stated infrastructure assumptions, whereas more intensive treatment increased CAPEX and OPEX.
[91]	Food waste → integrated biorefinery producing hydrogen, ethanol, and fertilizer	100 t food waste/day; shutdown threshold below 7.84 t/day (2587.2 t/year)	Hydrogen, ethanol, and fertilizer	USD; currency base year not explicitly reported in the accessible study information; 7% discount rate	Total capital investment: US$7.626 million; annual operating cost: US$2.432 million/year; after-tax net profit: US$3.142 million/year; payback period: 3.5 years; IRR: 45.54%; ROI: 43.88%	Hydrogen selling price was reported as the strongest economic driver; feed rate/capacity was also critical	The modeled 100 t/day integrated plant was economically favorable under the study assumptions, but profitability was highly sensitive to hydrogen price and plant throughput.
[92]	Restaurant food waste → waste-to-electricity, with anaerobic digestion compared with pyrolysis and gasification	Medium-scale installations; anaerobic digestion assessed for electricity generation, with reported output up to 5 MW under the study scenario	Electricity/renewable energy	USD; currency base year not explicitly reported in the accessible study information	Electricity generation cost: approximately US$0.08/kWh; ROI: 12–15% for anaerobic digestion; payback period: 5–7 years for medium-scale installations	Capital investment, operating cost, energy output, electricity economics, technology choice, and policy/regulatory support	Anaerobic digestion showed favorable modeled economics for restaurant-food-waste-to-electricity under the study assumptions; the reported results remain location- and policy-dependent.

Note: Monetary values are reported as stated in the cited studies and have not been converted to a common currency or price year. Where a single currency base year was not explicitly identifiable from the accessible study information, this is stated rather than inferred. The reported economic indicators are scenario-specific and should be interpreted together with plant scale, discount rate, feedstock assumptions, product prices, gate fees, process configuration, and other study-specific assumptions.

**Table 16:** Key sustainability indicators for evaluating integrated food waste biorefineries.

Dimension	Indicator	Typical Unit	Decision-Making Relevance	References
Environmental	Carbon footprint/Global Warming Potential (GWP)	kg CO_2_-eq/kg product	Quantifies climate-change impacts and enables comparison of alternative valorization pathways and identification of major GHG-emission hotspots.	[96,212]
Environmental	Water footprint	L/kg product	Quantifies freshwater consumption and/or potential water-scarcity impacts and identifies water-intensive process stages.	[229,230]
Environmental	Cumulative energy demand	MJ/kg product	Quantifies total life-cycle energy demand and helps identify energy-intensive process stages and opportunities for energy optimization.	[5,209]
Environmental	Eutrophication potential (EP) & acidification potential (AP)	kg N-eq, kg P-eq or kg PO_4_^3−^-eq/FU (EP); kg SO_2_-eq/FU (AP)	Quantifies nutrient-enrichment and acidification impacts and helps identify emission hotspots associated with nutrient losses, chemical inputs and residual-stream management.	[222,231]
Economic	Capital expenditure (CAPEX)	USD or EUR/plant; alternatively currency/unit of processing capacity	Quantifies initial capital requirements and supports comparison of investment intensity, scale-up feasibility and alternative process configurations.	[89,90]
Economic	Operating expenditure (OPEX)	Expenditure (OPEX) USD or EUR/year; alternatively currency/FU	Quantifies recurring operating costs and identifies major cost drivers affecting process profitability and optimization.	[227,228]
Economic	Minimum selling price (MSP)	USD or EUR/kg product	Determines the minimum product selling price required under defined economic assumptions and supports comparison with market prices and competing products.	[87,89]
Economic	Net present value (NPV), internal rate of return (IRR), return on investment (ROI)	Currency (NPV); % (IRR, ROI)	Evaluates long-term profitability, financial return and investment attractiveness of alternative biorefinery configurations.	[91,92]
Technical	Product yield/productivity	g or kg product/kg feedstock (yield); g L^−1^ h^−1^ or kg m^−3^ d^−1^ (productivity)	Quantifies conversion performance and production rate, supporting comparison of process efficiency, reactor performance and scale-up potential.	[88,89]
Technical	Technology readiness level (TRL)	1–9	Assesses technological maturity and proximity to industrial deployment, supporting scale-up and technology-selection decisions.	[232,233]
Technical	Scalability & process robustness	Qualitative/quantitative; scale-dependent performance and stability metrics	Evaluates the ability to maintain stable process performance during scale-up and under variable operating and feedstock conditions, supporting industrial implementation.	[93,234]
Circularity	Carbon utilization efficiency (CUE)	%	Quantifies the fraction of feedstock carbon retained in useful products and recovered streams, supporting evaluation of carbon losses and overall biomass utilization.	[78,93]
Circularity	Nutrient recovery efficiency	% of initial N, P and/or K recovered	Quantifies the fraction of feedstock nutrients recovered in reusable products or streams and identifies nutrient losses within the biorefinery.	[218,235]
Circularity	Waste diversion rate	%	Quantifies the proportion of food waste diverted from landfill or other disposal routes toward recovery, recycling or valorization pathways.	[236,237]
Circularity	Cascading-use efficiency/Biomass Utilisation Factor (BUF)	Index or dimensionless metric	Evaluates the extent to which biomass is sequentially retained in useful applications before final energy recovery or disposal, supporting efficient allocation of biomass across multiple valorization stages.	[238,239]
Circularity	Resource Circularity Index (RCI)/Circularity Index (CI)	Dimensionless index (typically 0–1)	Quantifies the degree to which resource flows are recovered and recirculated within the system and helps identify opportunities for closing resource loops.	[5,240]
Circularity	Energy self-sufficiency	% of total energy demand supplied internally	Quantifies the proportion of biorefinery energy demand met through internally recovered or generated energy, indicating reduced dependence on external energy inputs.	[241,242]

## Data Availability

No new data were created or analyzed in this study. Data sharing is not applicable to this article.

## Supplementary Materials

The following supporting information can be downloaded at: https://www.mdpi.com/article/10.3390/foods15203692/s1, Figure S1: Study-selection workflow used for the structured critical literature review, showing literature identification, duplicate removal, title/abstract screening, full-text eligibility assessment, and final inclusion in the qualitative narrative synthesis. This workflow is provided for methodological transparency and does not represent a PRISMA flow diagram. Table S1. Database-specific literature-search strategies used in this review *.
