# Supplementary material for: Integrated Biorefineries for Food Waste and Food-Processing Side Streams Through Feedstock-Oriented Design and Sustainable Process Integration
Source: Foods. 2026 Oct 16;15(20):3692. doi: 10.3390/foods15203692 (PMC13704576; doi:10.3390/foods15203692)
Supplement: Supplementary file 1 [file foods-15-03692-s001.zip › foods-4567462-supplementary.pdf]

Supplementary Materials

Integrated Biorefineries for Food Waste and Food-Processing Side Streams through Feedstock-Oriented Design and Sustainable Process Integration

Ersilia Daniela Lazăr Coșbuc, Paula Sânziana Oprea Blănaru, Elena-Diana Ungureanu-Comăniță, Carmen Teodosiu and Maria Gavrilescu

Methodological clarification.
The present study was designed as a structured critical literature review with a structured narrative comparative synthesis, rather than as a systematic review or meta-analysis. Accordingly, it was not conducted or registered under a systematic-review protocol. The study-selection workflow presented in Supplementary Figure S1 is provided to enhance the transparency and reproducibility of the literature identification, screening, eligibility assessment, and selection procedures and should not be interpreted as a PRISMA flow diagram.

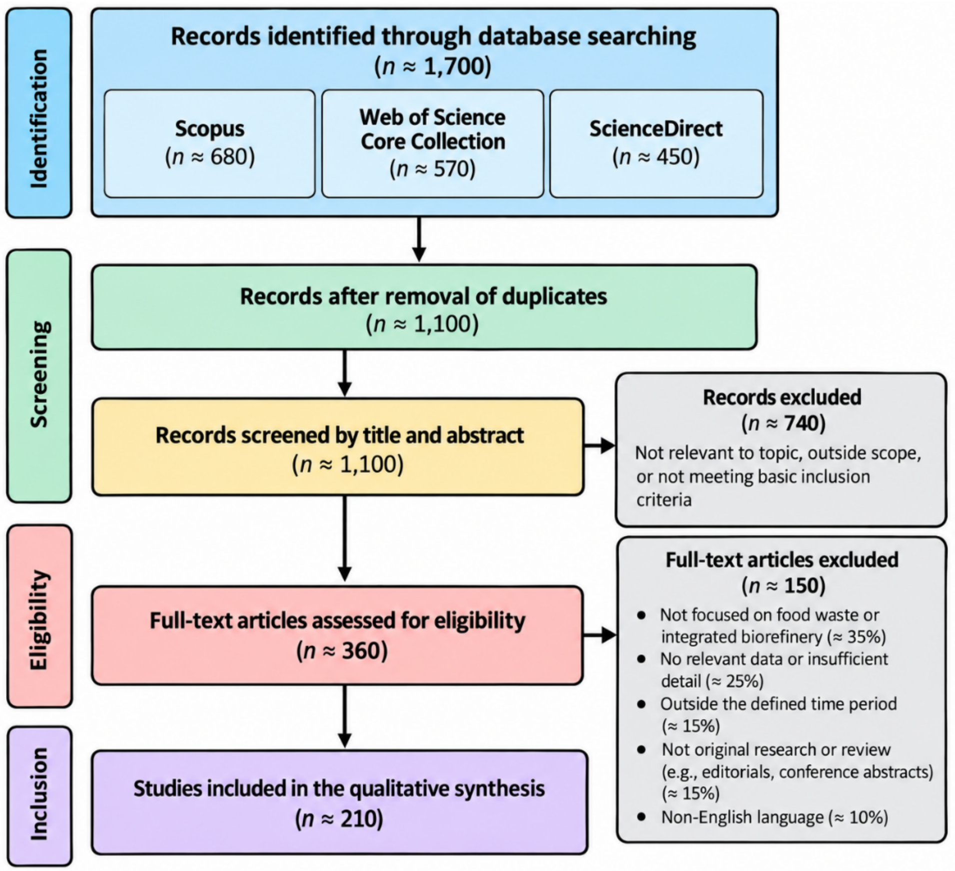

Supplementary Figure S1. Study-selection workflow used for the structured critical literature review, showing literature identification, duplicate removal, title/abstract screening, full-text eligibility assessment, and final inclusion in the qualitative narrative synthesis. This workflow is provided for methodological transparency and does not represent a PRISMA flow diagram.

Supplementary Table S1. Database-specific literature-search strategies used in this review*

Database	Search field(s)	Complete search strategy	Restrictions / filters	Search date / update period
Scopus	Title, Abstract, Keywords (TITLE-ABS-KEY)	TITLE-ABS-KEY(("food waste" OR "food-processing waste" OR "agri-food waste" OR "food residues" OR "municipal food waste") AND ("pretreatment" OR "fractionation" OR "fermentation" OR "anaerobic digestion" OR "thermochemical conversion") AND ("biorefinery" OR "biofuels" OR "biochemicals" OR "bioproducts" OR "resource recovery" OR "cascade valorization") AND ("process integration" OR "circular biorefinery" OR "life-cycle assessment" OR "techno-economic assessment" OR "technology readiness level"))	Publication period: 2015–September 2026; English language; research articles and review articles prioritized.	May–September 2026
Web of Science Core Collection	Topic (TS: title, abstract, author keywords, Keywords Plus)	TS=(("food waste" OR "food-processing waste" OR "agri-food waste" OR "food residues" OR "municipal food waste") AND ("pretreatment" OR "fractionation" OR "fermentation" OR "anaerobic digestion" OR "thermochemical conversion") AND ("biorefinery" OR "biofuels" OR "biochemicals" OR "bioproducts" OR "resource recovery" OR "cascade valorization") AND ("process integration" OR "circular biorefinery" OR "life-cycle assessment" OR "techno-economic assessment" OR "technology readiness level"))	Publication period: 2015–September 2026; English language; research articles and review articles prioritized.	May–September 2026
ScienceDirect	Title, abstract, and author-specified keywords / platform search fields	(("food waste" OR "food-processing waste" OR "agri-food waste" OR "food residues" OR "municipal food waste")) AND (("pretreatment" OR "fractionation" OR "fermentation" OR "anaerobic digestion" OR "thermochemical conversion")) AND (("biorefinery" OR "biofuels" OR "biochemicals" OR "bioproducts" OR "resource recovery" OR "cascade valorization")) AND (("process integration" OR "circular biorefinery" OR "life-cycle assessment" OR "techno-economic assessment" OR "technology readiness level"))	Publication period: 2015–September 2026; English language; research articles and review articles prioritized.	May–September 2026

*Search period: January 2015–September 2026. Database searches and updates were conducted between May and September 2026. Language restriction: English.

Notes. Reference lists of key review articles and highly relevant primary studies were additionally screened to identify pertinent publications. Relevant institutional reports and policy documents from recognized international and governmental organizations were consulted where needed for definitions, statistics, regulatory context, and policy frameworks. Earlier foundational references identified through backward reference screening could be retained when necessary to document benchmark processes, established technologies, or historical concepts.
